# High Abundance of Unusual High Mannose *N*-Glycans Found in Beans

**DOI:** 10.1021/acsomega.4c04114

**Published:** 2024-11-06

**Authors:** Chia Yen Liew, Hong-Sheng Luo, Jien-Lian Chen, Chi-Kung Ni

**Affiliations:** † Institute of Atomic and Molecular Sciences, Academia Sinica, Taipei 10617, Taiwan; § Department of Chemistry, National Taiwan Normal University, Taipei 11677, Taiwan; ∥ Department of Chemistry, National Tsing Hua University, Hsinchu, 30013, Taiwan

## Abstract

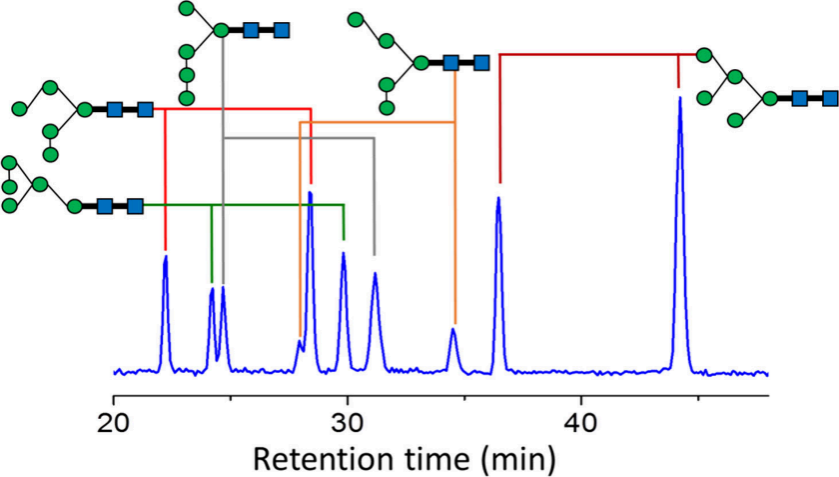

High mannose *N*-glycans extracted from eight different beans (black bean, soybean, pea, white kidney bean, pinto bean, mung bean, white hyacinth bean, and red bean) were studied using the state-of-the-art mass spectrometry method logically derived sequence tandem mass spectrometry (LODES/MS^n^). These beans show very similar *N*-glycan isomer profiles: one isomer of Man_9_GlcNAc_2_ and Man_8_GlcNAc_2_, two isomers of Man_7_GlcNAc_2_, three isomers of Man_6_GlcNAc_2_, and five isomers of Man_5_GlcNAc_2_ were found. Isomers not predicted by current *N*-glycan biosynthetic pathways were found in all beans, indicating the possibility of alternative biosynthetic pathways in these plants. The high abundance of unusual high mannose Man_5_GlcNAc_2_*N*-glycans in beans is particularly useful for the large-scale preparation of high mannose *N*-glycans that are not easily found in the other biological systems.

## Introduction

*N*-Linked glycosylation of proteins is one of the most important post-translational modifications of proteins and plays important role in many biological functions.^1^ Studying these relevant biological functions necessitates a significant amount of *N*-glycans. Substantial quantities of glycans can be obtained by chemical and chemoenzymatic synthesis^2−8^ or by extraction from natural products.^9−11^ Food with a high protein content, such as beans, is widely available and can be used as the natural source for *N*-glycans. For example, soybean has been proposed as a source for the large-scale preparation of high mannose *N*-glycans.^12−14^ Furthermore, recent advances in glycoengineering have proposed the use of rice cell culture systems for the production of biopharmaceuticals. Choi et al. used mannosidase inhibitors, i.e., kifunensine and swainsonine, in plant systems to produce high mannose *N*-glycans that are needed for therapeutic enzymes, such as rhGAA in the treatment of Pompe disease.^15^ This underscores the need to determine the structure of high mannose *N*-glycans in plant-based systems to ensure that the produced therapeutic enzymes have compatible glycan profiles.

On the other hand, soybean is also known as an allergenic food. Glycosylation of proteins has been reported to be closely related with food allergies.^15−21^ The *N*-glycans of plants are known to be different from those of mammals in two aspects. One is that the fucose at the core of *N*-glycans in plants is α-1,3-linked, whereas that in mammals is α-1,6-linked. The other is the β-1,2 linkage of xylose in plant *N*-glycans, which does not exist in the *N*-glycans of mammals. It has been found that *N*-glycans with α-1,3-fucose and β-1,2-xylose are important epitopes in glycoproteins of glycoallergens.^22−25^ Recently, high abundances of unusual structures of Man_5_GlcNAc_2_*N*-glycans were found in red bean.^26^ Whether the unusual structures of Man_5_GlcNAc_2_*N*-glycans different from mammal’s *N*-glycans play roles in glycoallergens is not clear.

Determination of the glycan structures is essential for both the study of glycoallergens and the large-scale preparation of *N*-glycans. However, structural determination of glycans is difficult due to the large number of isomers and the small difference in structures between these isomers.^27,28^ Traditionally, the structures of *N*-glycans were analyzed using nuclear magnetic resonance spectroscopy or a combination of enzyme digestion and mass spectrometry.^29−36^ Recently, a new mass spectrometry method, namely, logically derived sequence tandem mass spectrometry (LODES/MS^n^), was developed for the detailed structural analysis of glycans,^37−42^ and this method has been applied to determine the structures of high mannose *N*-glycans extracted from biological samples.^26^ A database including HPLC retention time and MS^2^, MS^3^, and MS^4^ CID spectra for the high mannose *N*-glycans was constructed by using LODES/MS^n^ for rapid isomer identification.^43^ In this study, we applied this database to investigate the structures of high mannose *N*-glycans extracted from various beans. The results show that a high abundance of unusually high mannose *N*-glycans is commonly found in beans. Comparison to other seeds that are used as food shows that beans are the best natural source for unusually high mannose *N*-glycans Man_5_GlcNAc_2_

## Experimental Method

All beans and seeds were purchased from local markets. These include black bean (*Glycine max*, black soybean Tainan 3), soybean (*Glycine max*), pea (*Pisum sativum*), white kidney bean (*Phaseolus vulgaris L*.), pinto bean (*Phaseolus vulgaris*), mung bean (*Vigna radiate*), white hyacinth bean (*Lablab purpureus*), red bean (*Vigna angulariz*), pine nut (*Pinus Koraiensis Siebold et Zuccarini*), corn (*Zea mays*), rice (*Oryza sativa L*.), seed of pumpkin (*Cucurbita spp. A*.), and walnut (*Juglans*). These materials were lyophilized and ground into powders separately. The *N*-glycans were released from proteins using an ammonia-catalyzed reaction^14^ or enzyme PNGase F. In brief, the sample was dissolved in a 25% ammonia aqueous solution for a 16 h reaction at 60 °C. After the reaction, the ammonia in the solution was removed using a rotary evaporator. For the *N*-glycans released using PNGase F (New England Biolabs, Ipswich, MA, USA), the sample and PNGase F in a solution consisting of 50 mM sodium phosphate (pH 7.5) reacted during 24 h of incubation at 37 °C. The released *N*-glycans were purified using ethanol precipitation to remove proteins, solid-phase extraction with a C18 cartridge (Sep-Pak C18, Waters, Milford, MA, USA) for the removal of residual proteins, and size exclusion chromatography for the removal of salt and small oligosaccharides.^26^ The extracted *N*-glycans were first separated into fractions according to their sizes by high-performance liquid chromatography (HPLC) with a TSKgel Amide-80 column (150 mm × 2.0 mm, particle size of 5 μm) using the following conditions: the flow rate was 0.2 mL/min, and the gradient changed linearly from A (H_2_O) = 35% and B (acetonitrile) = 65% at *t* = 0 to A = 45% and B = 55% at *t* = 50 min. The separation of *N*-glycans by their different sizes eliminates the potential interference by ESI in-source decay of large *N*-glycans.^44^ The eluents collected from HPLC were then sent into another HPLC with a PGC Hypercarb column (2.1 mm × 100 mm, particle size of 3 μm) separately for isomer separation. The HPLC conditions for the PGC Hypercarb column were as follows: the flow rate was 0.2 mL/min, and the gradient was changed linearly from A = 100% and B = 0% at *t* = 0 to A = 82%, B = 18% at *t* = 30 min. The PGC column was coupled to a linear ion trap mass spectrometer, and the eluents from the PGC column were sent into the mass spectrometer directly to record the retention time and mass spectra.

## Results

**Figure 1:**
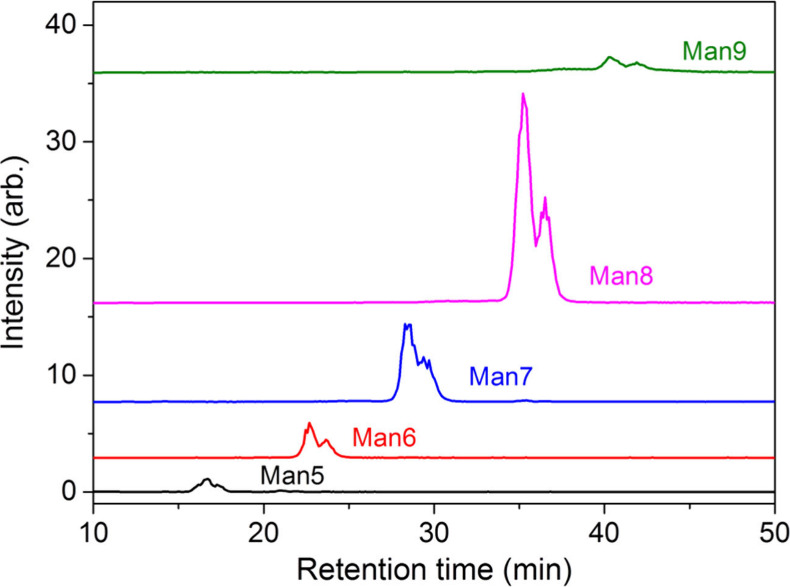
Chromatogram of the high mannose *N*-glycans extracted from red beans. HPLC with a TSKgel Amide-80 column was used to separate *N*-glycans into different sizes.

Figure 1 shows the chromatograms of the high mannose *N*-glycans extracted from red bean and separated by HPLC using a TSKgel Amide-80 column. These *N*-glycans were separated according to their sizes. The separation of the *N*-glycans by size prior to the separation of isomers by the PGC column is necessary to remove the interference of small *N*-glycans by large *N*-glycans in structural determination due to ESI in-source decay.^44^ The relative intensities show high abundances of Man_6_GlcNAc_2_ (Man6), Man_7_GlcNAc_2_ (Man7), and Man_8_GlcNAc_2_ (Man8) and low abundances of Man_5_GlcNAc_2_ (Man5) and Man_9_GlcNAc_2_ (Man9). Among the eight different beans we studied, all beans showed similar relative abundances among these *N*-glycans, although the abundances of Man_9_GlcNAc_2_ in mung bean, *Lablab purpureus*, and pea were too small to be detected.

The eluents from the TSKgel Amide-80 column were collected fractionally according to the size of the *N*-glycans and then subjected to secondary HPLC using a PGC column for isomer separation and identification. The high mannose *N*-glycan database in our previous report^43^ was applied to determine the structures of *N*-glycans extracted from beans. Notably, we used intact *N*-glycans, i.e., no reduction, permethylation, or derivatization. The GlcNAc at the reducing end of intact *N*-glycans can be either the α-anomeric configuration or the β-anomeric configuration, which coexist in solution and reach equilibrium. Consequently, if separated by HPLC, these two anomers will manifest as two distinct peaks in the chromatogram. The *N*-glycan structures were identified by comparing the measured data to the database using the following criteria: (1) retention times of the ions with the selected *m*/*z* values compared with those in the database, (2) relative intensities of α- and β-anomers of each isomer, and (3) MS^2^ and MS^3^ (and MS^4^ for some peaks) mass spectra at the corresponding retention time.

**Figure 2:**
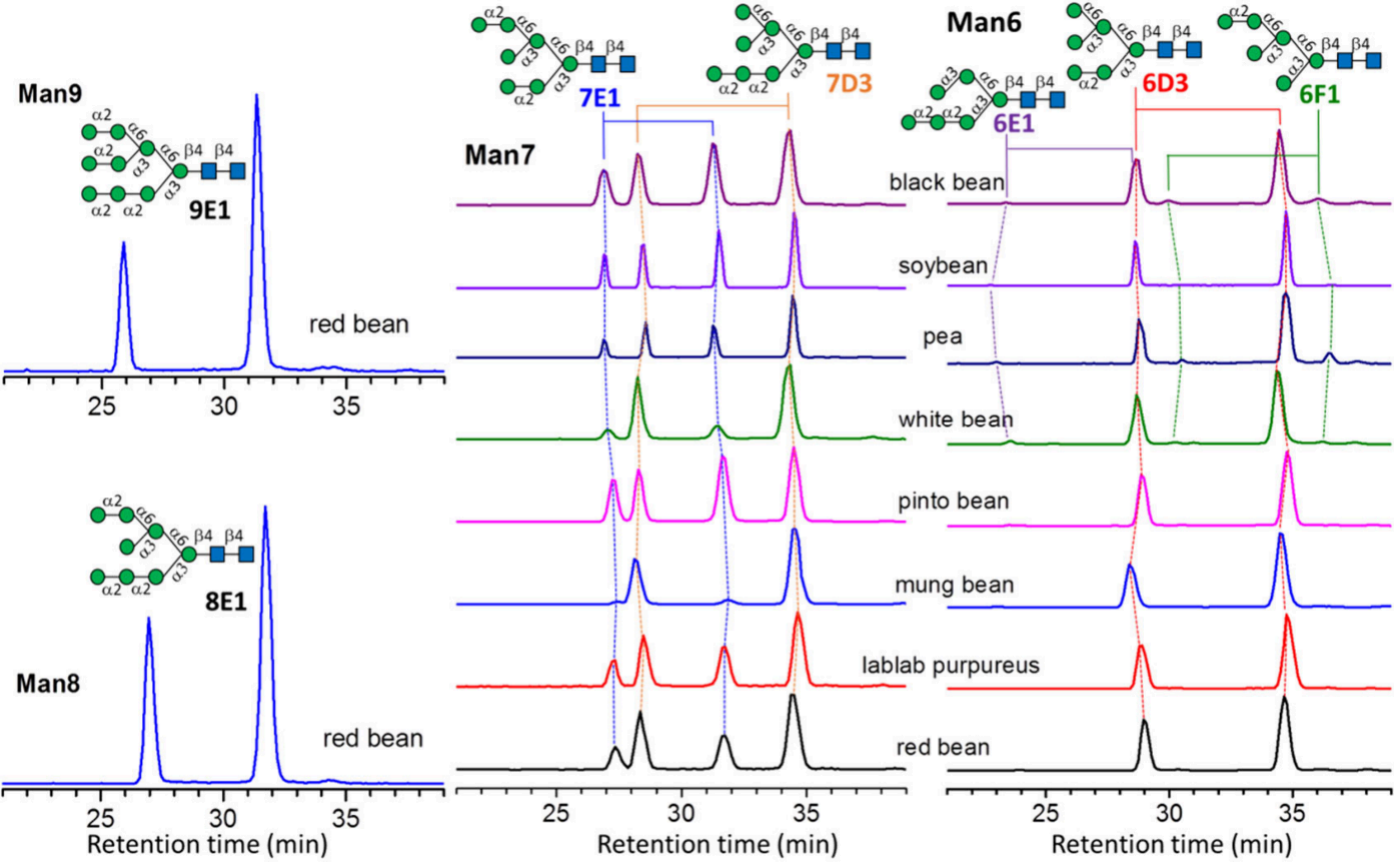
Chromatograms and structure assignments of high mannose *N*-glycans Man_*n*_GlcNAc_2_, *n* = 6–9. HPLC with a PGC column was used to separate *N*-glycan isomers.

**Figure 3:**
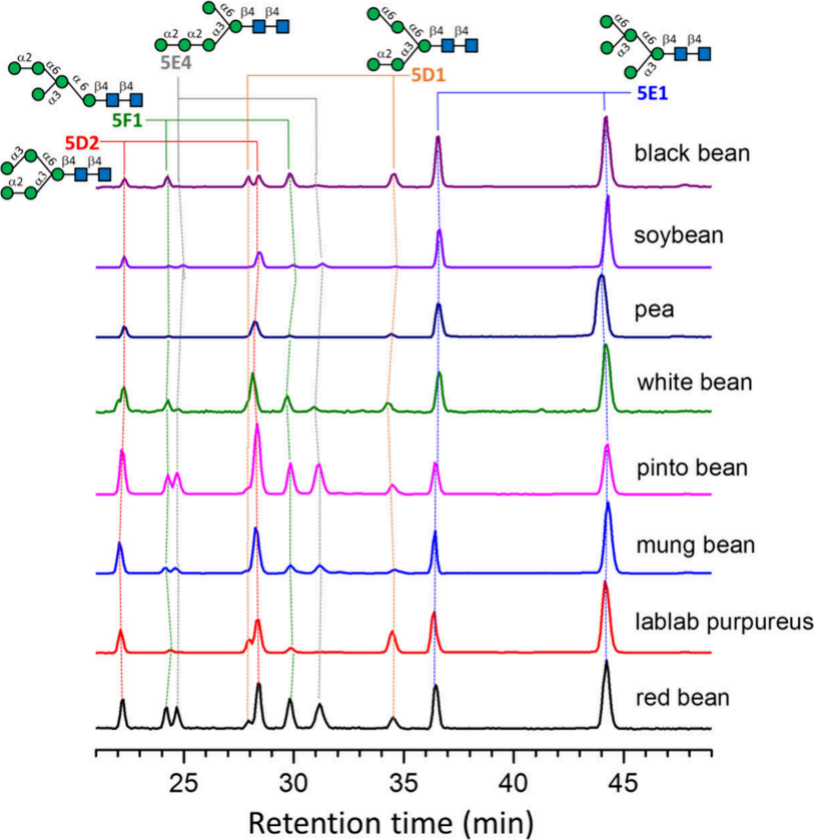
Chromatograms and structure assignments of high mannose *N*-glycans Man_5_GlcNAc_2_. HPLC with PGC column was used to separate *N*-glycan isomers.

The eight different beans we studied show very similar distributions of high mannose *N*-glycans isomers. Only one isomer of Man_9_GlcNAc_2_ (9E1) and one isomer of Man_8_GlcNAc_2_ (8E1) were found in the beans. Here, 9E1 and 8E1 are abbreviations of the nomenclature (see a detailed definition of the nomenclature in ref (26)). The chromatogram and the structure assignments of the red bean, which is used as an example, are illustrated in Figure 2. There are two isomers of Man_7_GlcNAc_2_ (7E1 and 7D3). The relative abundances of these two isomers are about the same except in mung bean and white bean, where the abundance of isomer 7E1 is low. The Man_6_GlcNAc_2_ is dominated by isomer 6D3, but small amounts of isomer 6E1 and 6F1 were found in black bean, soybean, pea, and white bean. The most diverse isomers are found in Man_5_GlcNAc_2_. Five isomers (5D1, 5D2, 5E1, 5E4, and 5F1) were found. The relative abundances of these isomers change with the beans, as illustrated in Figure 3. To estimated the absolute abundance of the high mannose *N*-glycans in red beans, we used 1 × 10^–6^ M pentasaccharide GalNAcα-(1 → 3)-[Fucα-(1 → 2)]-Galβ-(1 → 3)-GlcNAcβ-(1 → 3)-Gal as an internal standard for calibration. The amounts of Man_8_GlcNAc_2_ and Man_5_GlcNAc_2_ were estimated to be 7.8 and 0.2 μg for every gram of dried red bean, respectively. Details of other high mannose *N*-glycans and other beans are listed in Table S1 of the Supporting Information. The trend that the amount of high mannose *N*-glycans decreases as the number of mannoses in *N*-glycans decreases is consistent with previous study, but the absolute amounts of Man_8_GlcNAc_2_ and Man_5_GlcNAc_2_ are different from those for the *N*-glycans extracted from soybean in a previous study,^13^ which are 137.9 μg of Man_8_GlcNAc_2_ and 0.0167 μg of Man_5_GlcNAc_2_. The differences might be due to the differences between soybean and red bean, the different sample losses between different extraction methods, the different *N*-glycan release efficiencies between oxidative release and enzyme releases, and the different ionization efficiencies of *N*-glycans and the internal standard GalNAcα-(1 → 3)-[Fucα-(1 → 2)]-Galβ-(1 → 3)-GlcNAcβ-(1 → 3)-Gal we used in this study.

The current biosynthetic pathways of multicellular eukaryote *N*-glycans are divided into three distinct stages.^45−50^ The first stage is the assembly of lipid a dolichol-phosphate-linked oligosaccharide, Glc_3_Man_9_GlcNAc_2_, followed by the en bloc transfer of this oligosaccharide to proteins. The second stage involves trimming Glc_3_Man_9_GlcNAc_2_ to Man_5_GlcNAc_2_, and finally Man_5_GlcNAc_2_ is converted to hybrid and complex *N*-glycans in the third stage.

**Figure 4:**
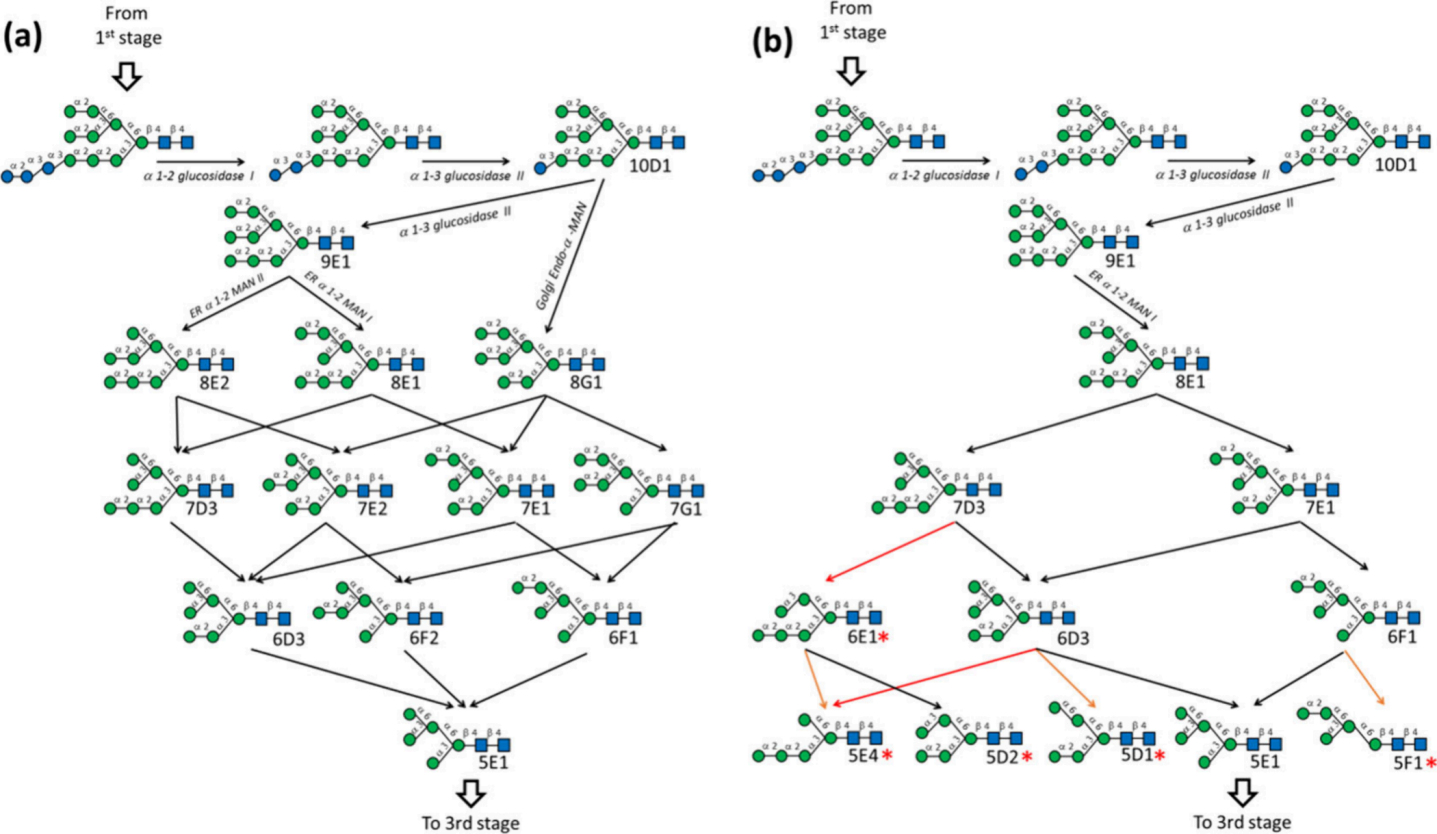
(a) Current second-stage biosynthetic pathways of multicellular eukaryote *N*-glycans and (b) proposed second-stage biosynthetic pathways of bean *N*-glycans based on the observed high mannose *N*-glycans. The *N*-glycans labeled by red stars represent the *N*-glycans not predicted by the current multicellular eukaryote biosynthetic pathways that were found in this study. Arrows in orange and red represent degradation by α-1,3-mannosidases and α-1,6-mannosidases, respectively.

During the second stage, the canonical biosynthetic pathways suggest that the sequential action of glucosidase I and II, which remove the glucose (Glc) residues, results in the formation of Man_9_GlcNAc_2_, denoted as 9E1 [Figure 4(a)]. Subsequent removal of terminal mannose residues from Man_9_GlcNAc_2_ by α-1,2-mannosidases results in the creation of Man_8_GlcNAc_2_ isomers labeled 8E1 and 8E2. A third isomer of Man_8_GlcNAc_2_, named 8G1, is produced through the action of endo-α-mannosidase on Glc1Man_9_GlcNAc_2_. Following this, various α-1,2-mannosidases in the endoplasmic reticulum (ER) and Golgi apparatus collaborate to excise all mannose residues linked by α-1,2 glycosidic bonds, thereby converting Man_8_GlcNAc_2_ into a single isomer of Man_5_GlcNAc_2_, referred to as 5E1. According to these biosynthetic pathways, there are three possible isomers for Man_8_GlcNAc_2_, four isomers for Man_7_GlcNAc_2_, three isomers for Man_6_GlcNAc_2_, and a single isomer for Man_5_GlcNAc_2._

**Figure 5:**
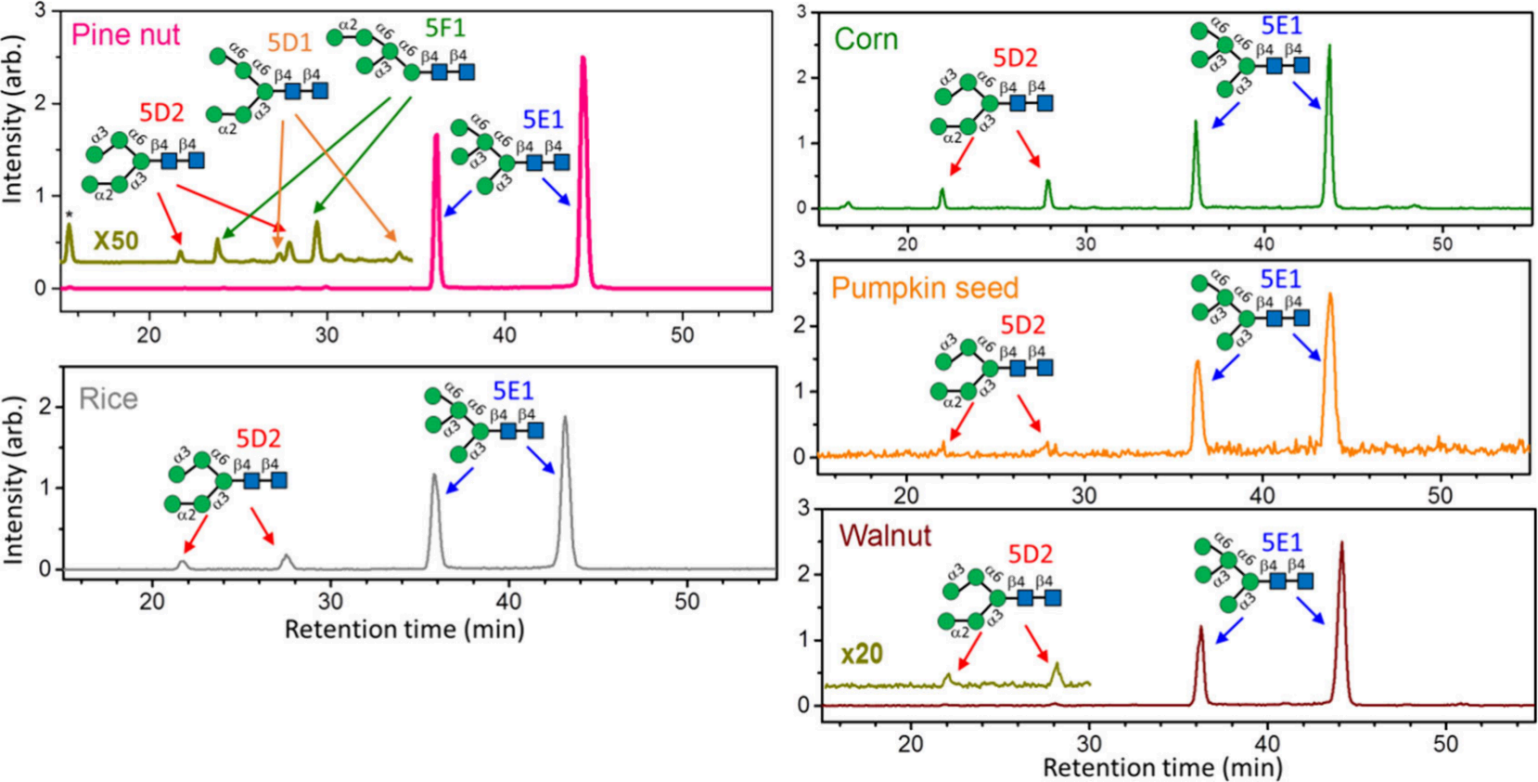
Chromatograms and isomer distribution of high mannose *N*-glycans extracted from pine nut, rice, corn, pumpkin seed, and walnut.

To find out how special the unusual high mannose Man_5_GlcNAc_2_*N*-glycans in beans are, we compared the high mannose Man_5_GlcNAc_2_*N*-glycans extracted from the other seeds that are commonly used as food. The selection of seeds is based on the easy availability and diversity distribution in the phylogenetic tree. Pine nut was used to represent *Gymnospermae*. Corn and rice were used to represent *Monocotyledons* of Angiospermae, while seeds of pumpkin and walnut were used to represent *Dicotyledoneae* of Angiospermae. The high mannose Man_5_GlcNAc_2_*N*-glycan profiles obtained from these seeds are illustrated in Figure 5. They are dominated by isomer 5E1, and only a small amount of 5D2 was found. Many isomers similar to those in beans were found in pine nuts, but the abundances are much lower than those of beans. Although we did not investigate many species, the current finding suggests beans have high abundances of unusual Man_5_GlcNAc_2_*N*-glycans and are the best natural source for extracting these unusual Man_5_GlcNAc_2_*N*-glycans.

High abundances of the high mannose *N*-glycans not described in the current *N*-glycan biosynthetic pathways were found in all eight different beans we studied, indicating that these unusual high mannose *N*-glycans commonly exist in beans. These unusual *N*-glycans are generated by removing the α-1,3- or α-1,6-linked terminal mannose before the complete removal of the α-1,2-linked terminal mannose. One possibility is that some α-1,3-mannosidases and α-1,6-mannosidases compete with the α-1,2-mannosidases in the ER or Golgi apparatus, revealing an interesting enzyme to be discovered. The high abundances of unusual high mannose Man_5_GlcNAc_2_*N*-glycans in beans are particular useful for the large-scale preparation of the high mannose *N*-glycans not easily found in the other biological systems.

## Supporting Information Available

Absolute amounts of high mannose *N*-glycans in dried beans, the relative intensities of various high mannose *N*-glycans in beans, and chromatograms and CID mass spectra of various high mannose *N*-glycans in beans (PDF)
