# Supplementary material for: High Abundance of Unusual High Mannose N-Glycans Found in Beans
Source: ACS Omega. 2024 Nov 6;9(46):45822–7. doi: 10.1021/acsomega.4c04114 (PMC11579719; doi:10.1021/acsomega.4c04114)
Supplement: Supplementary file 1 — ao4c04114_si_001.pdf [file ao4c04114_si_001.pdf]

Supplementary Information

High Abundance of Unusual High Mannose *N*-glycans Found in Beans

Chia Yen Liew[1,2], Hong-Sheng Luo[1,3], Jien-Lian Chen[1], and Chi-Kung Ni*[1,4]

[1] Institute of Atomic and Molecular Sciences, Academia Sinica, Taipei 10617, Taiwan

[2] Current address: Biology and Biological Engineering and Proteome Exploration Laboratory Beckman Institute California Institute of Technology, 1200 East California Boulevard, Pasadena, 91125, CA, USA.

[3] Department of Chemistry, National Taiwan Normal University, Taipei, Taiwan

[4] Department of Chemistry, National Tsing Hua University, Hsinchu, 30013, Taiwan

*Corresponding authors, e-mail addresses: ckni@po.iams.sinica.edu.tw

Table S1. N-glycans (in the unit of g) in 1g of dried bean

	black bean	soybean	pea	white bean	pinto bean	mung bean	purpreus	red bean
Man5	5.0E-07	9.9E-07	3.3E-07	1.6E-07	3.5E-07	1.2E-07	5.0E-07	2.2E-07
Man6	2.2E-06	4.3E-06	1.2E-06	7.1E-07	1.7E-06	1.5E-06	4.5E-06	2.2E-06
Man7	2.2E-06	5.2E-06	8.0E-07	1.3E-06	1.7E-06	3.3E-06	1.5E-06	5.2E-06
Man8	2.8E-06	5.4E-06	7.8E-07	7.3E-07	7.8E-07	2.3E-06	5.2E-07	7.8E-06

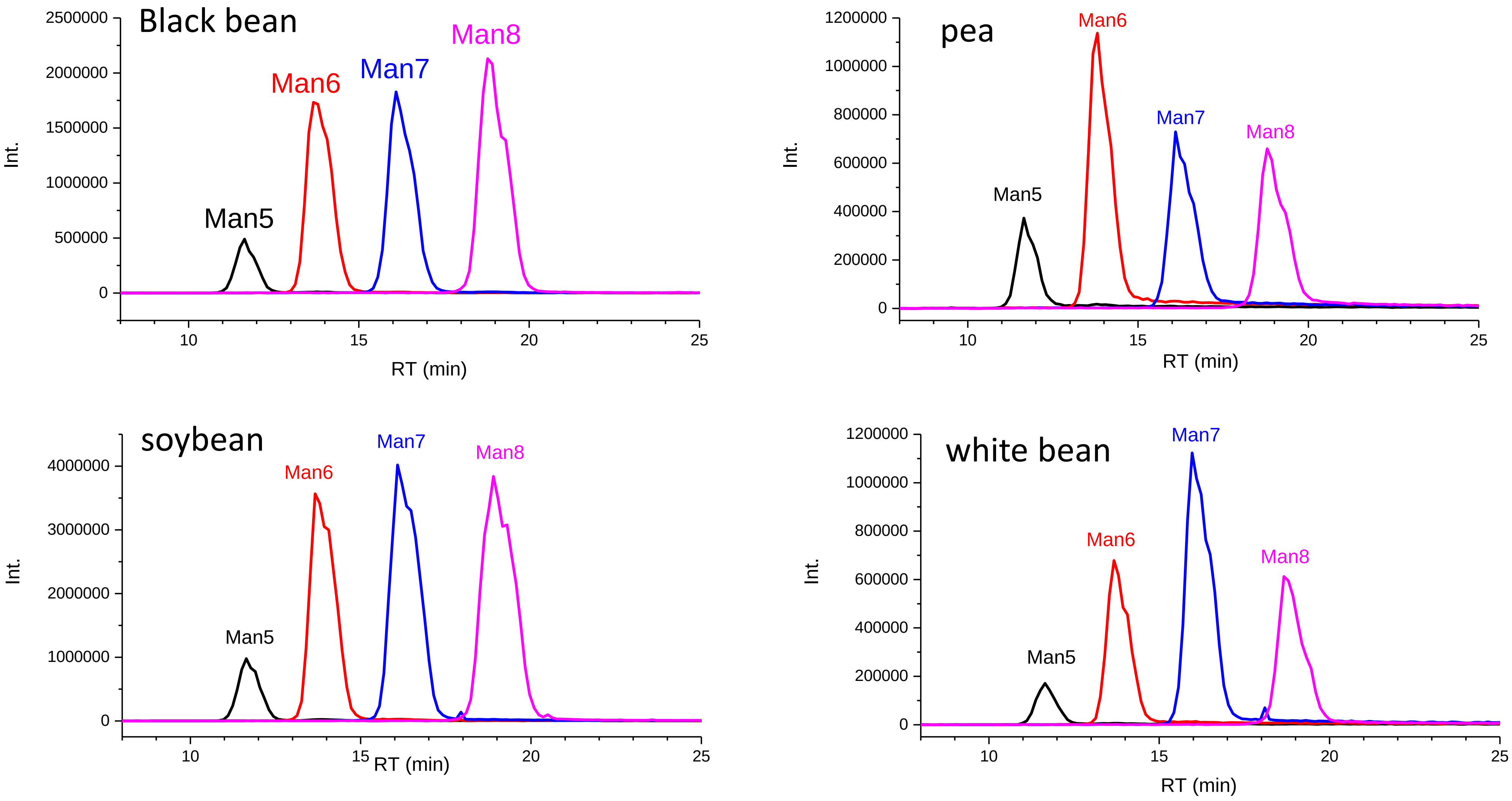

Figure S1. Relative intensities of high mannose N-glycans for various beans.

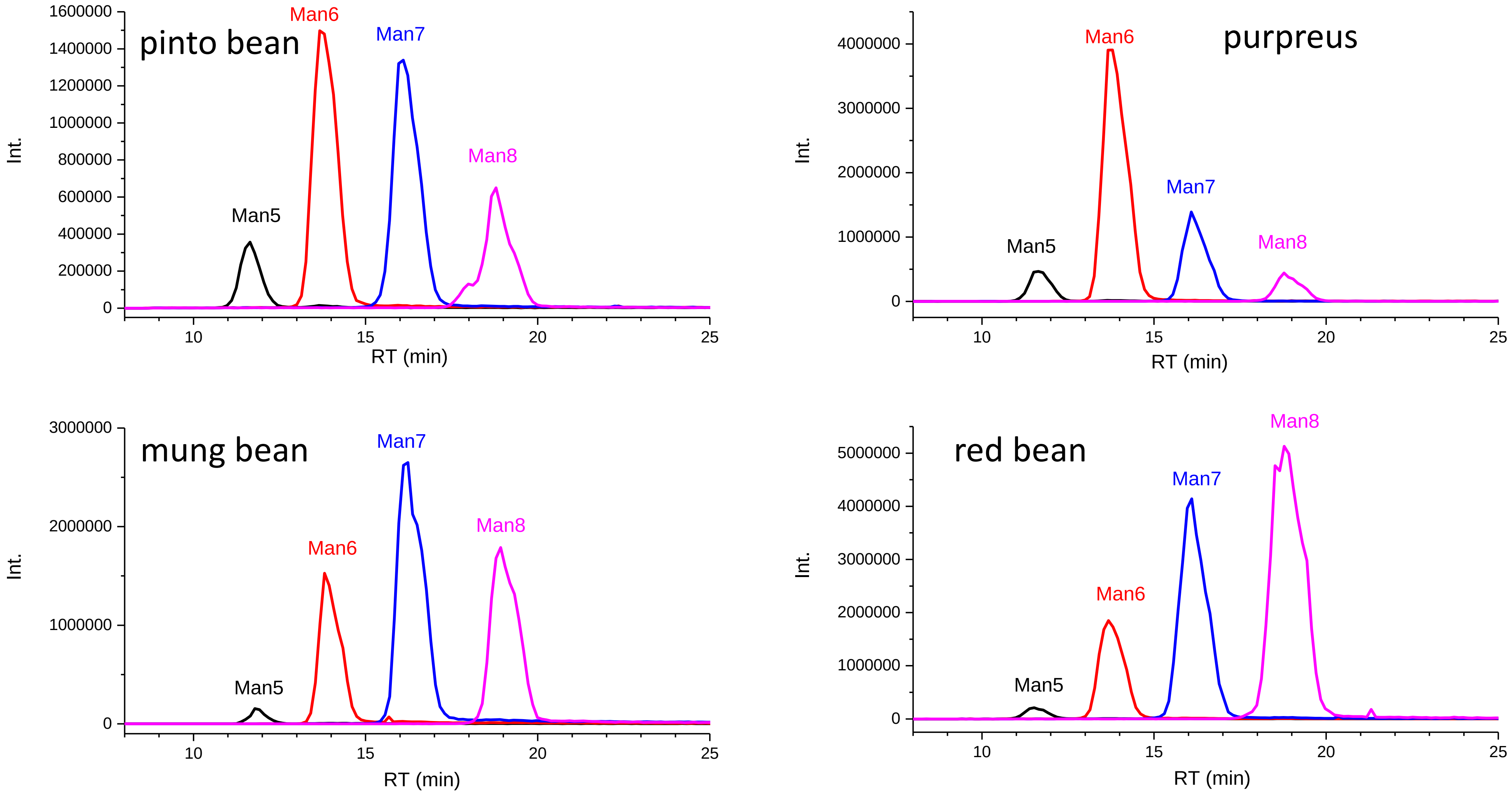

Figure S2. Relative intensities of high mannose N-glycans for various beans.

$Man_5GlcNAC_2$

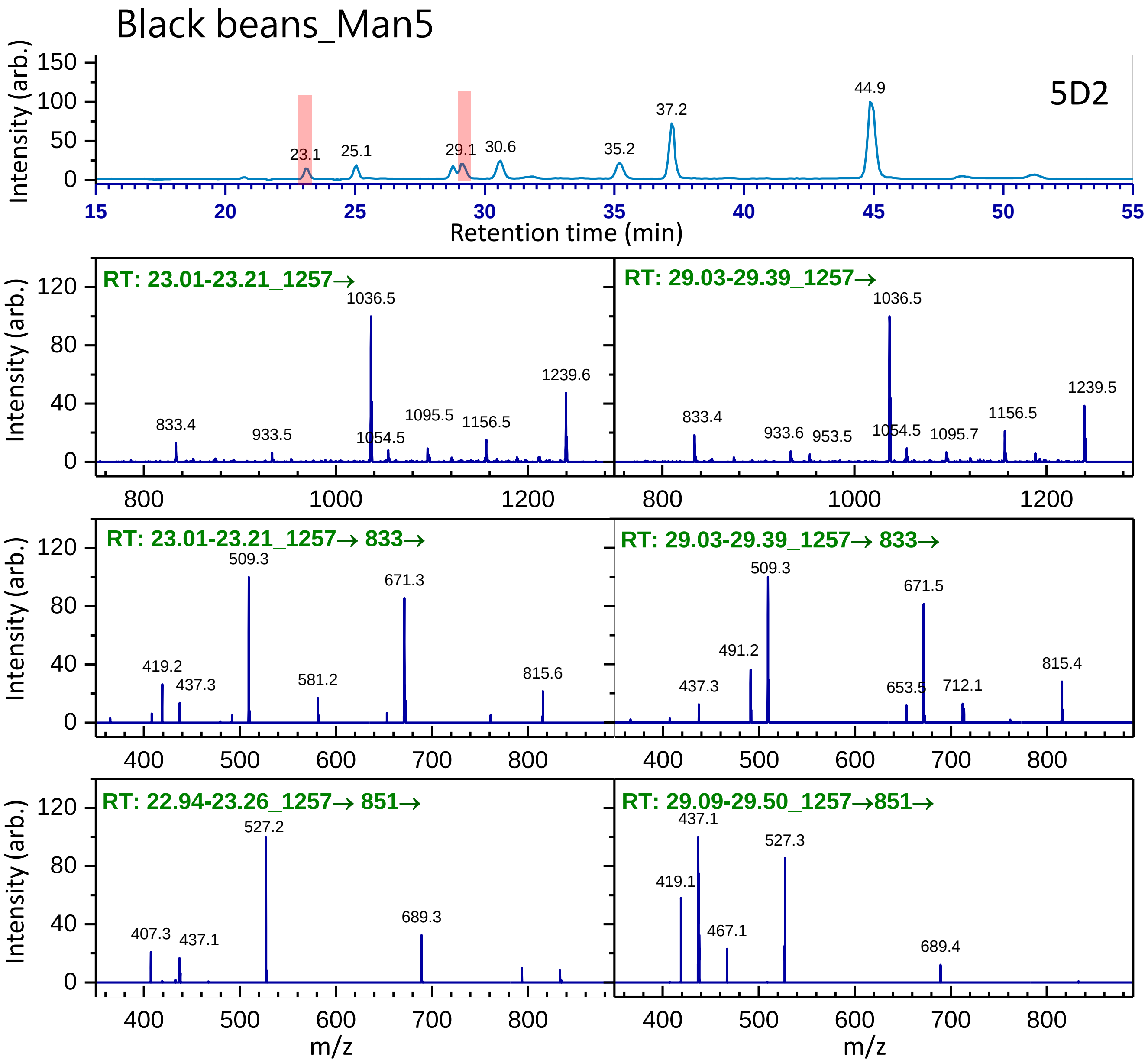

Figure S3. Chromatogram and MS^2 and MS^3 mass spectra of black bean $Man_5GlcNAC_2$.

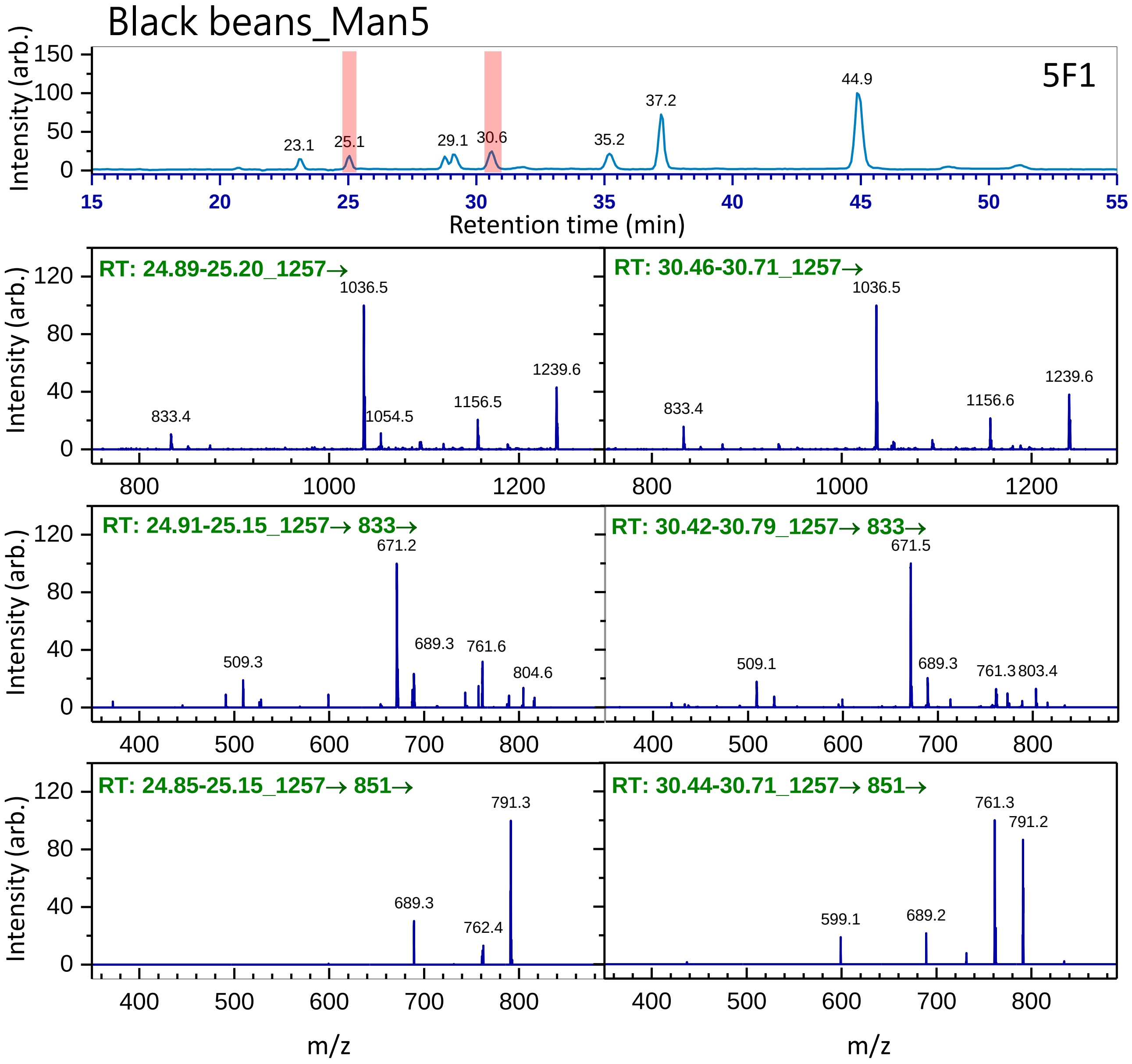

Figure S4. Chromatogram and MS^2 and MS^3 mass spectra of black bean $Man_5GlcNAC_2$.

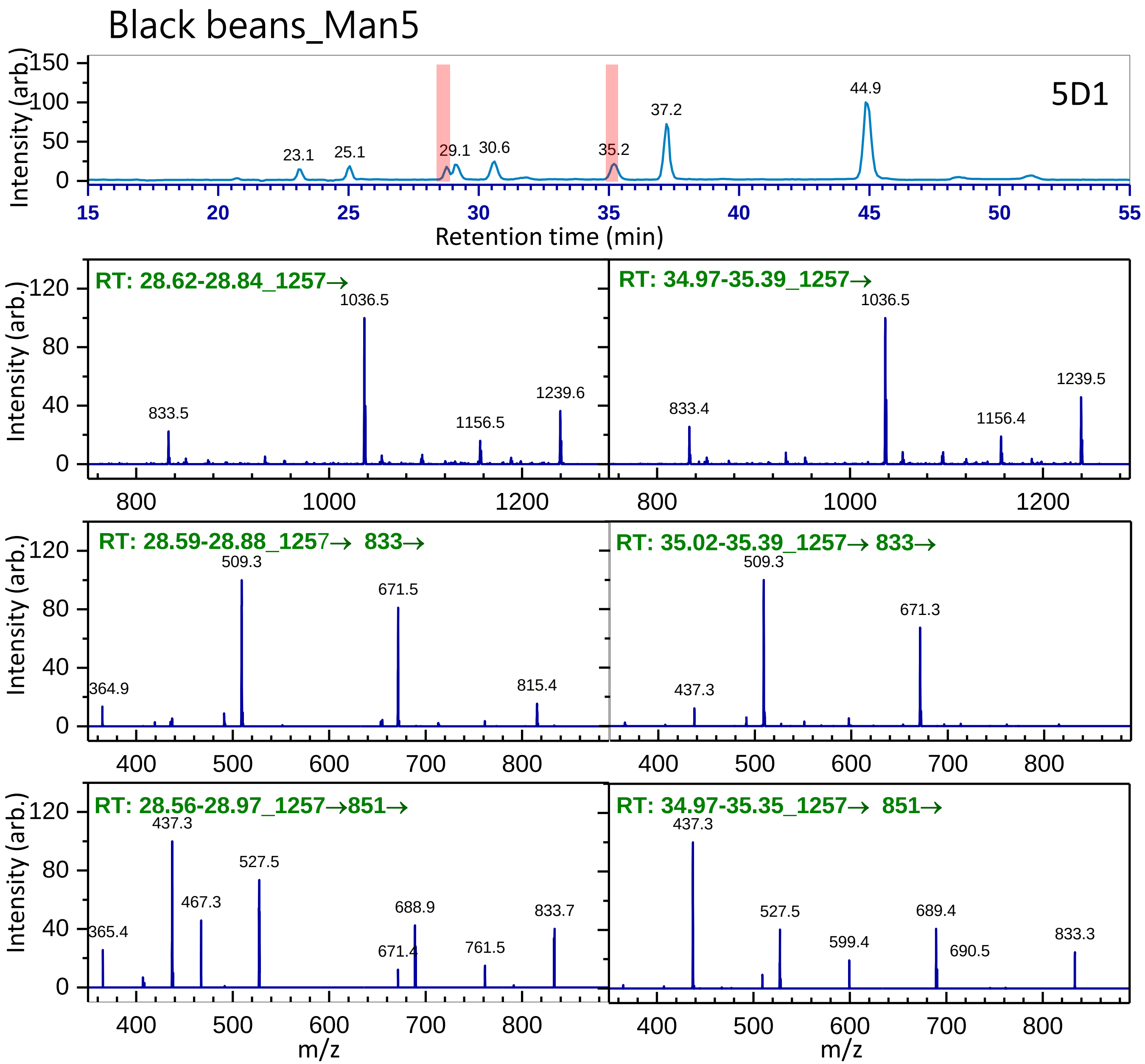

Figure S5. Chromatogram and MS^2 and MS^3 mass spectra of black bean $Man_5GlcNAC_2$.

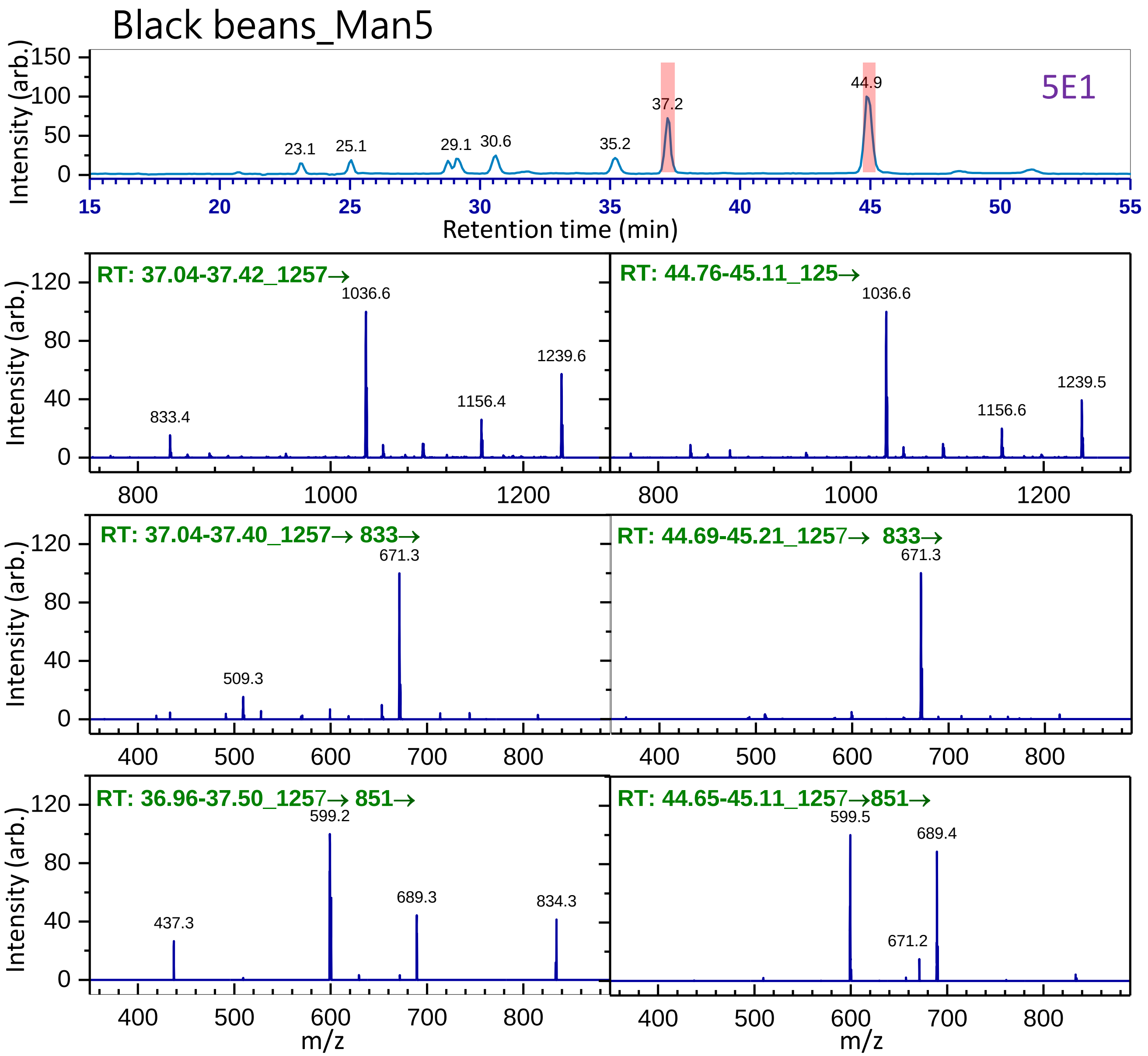

Figure S6. Chromatogram and MS^2 and MS^3 mass spectra of black bean $Man_5GlcNAC_2$.

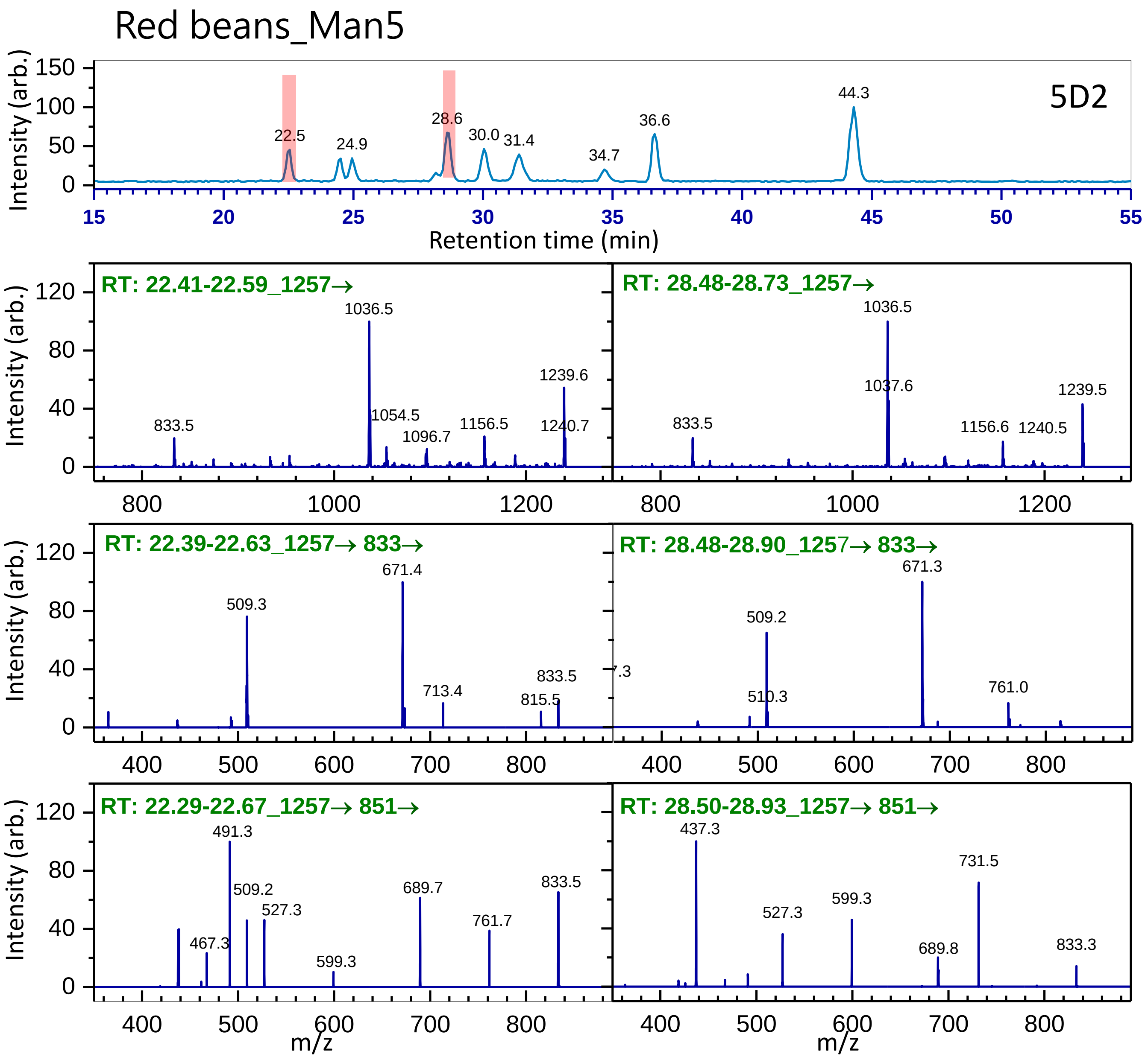

Figure S7. Chromatogram and MS^2 and MS^3 mass spectra of red bean $Man_5GlcNAC_2$.

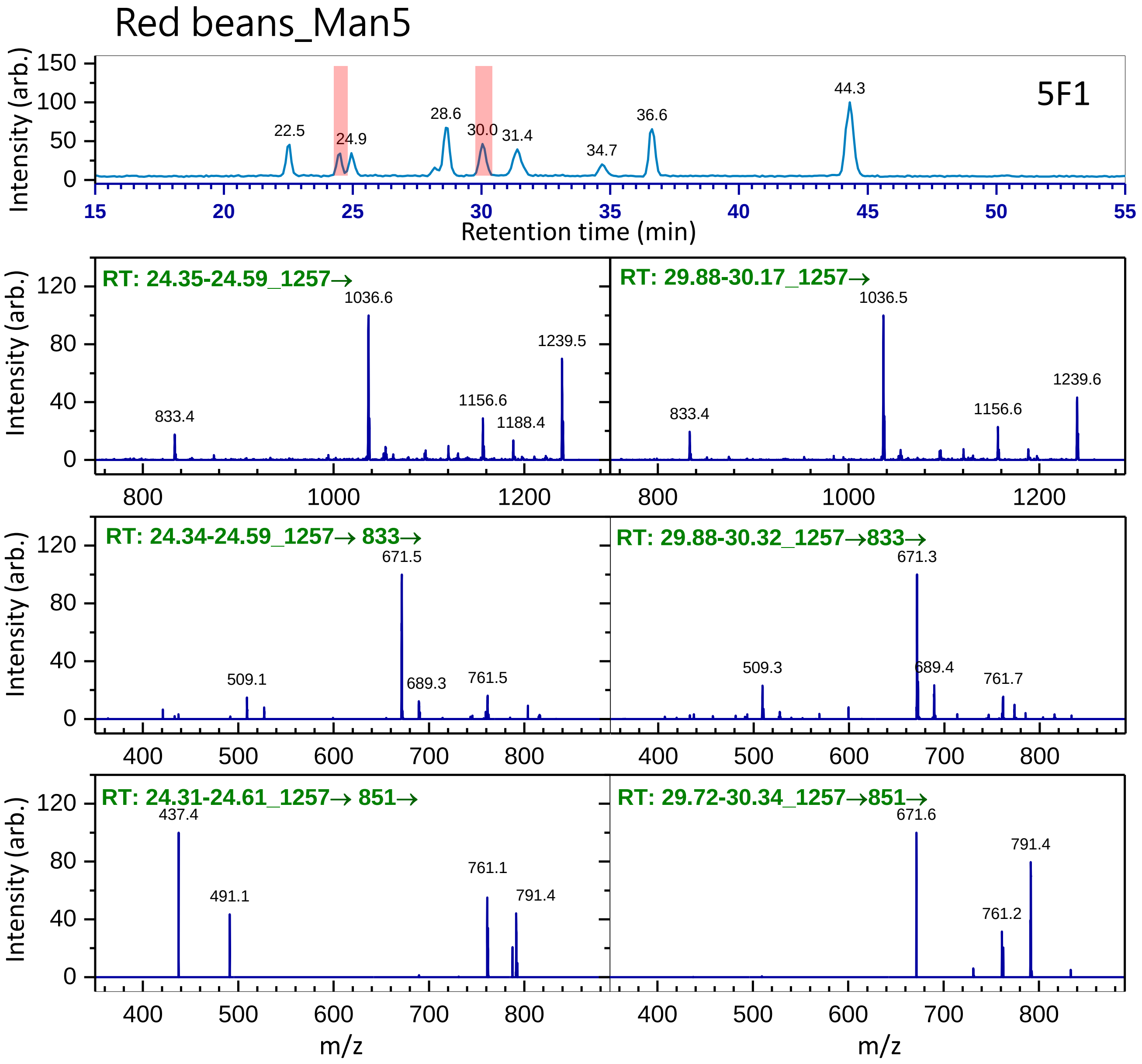

Figure S8. Chromatogram and MS^2 and MS^3 mass spectra of red bean $Man_5GlcNAC_2$.

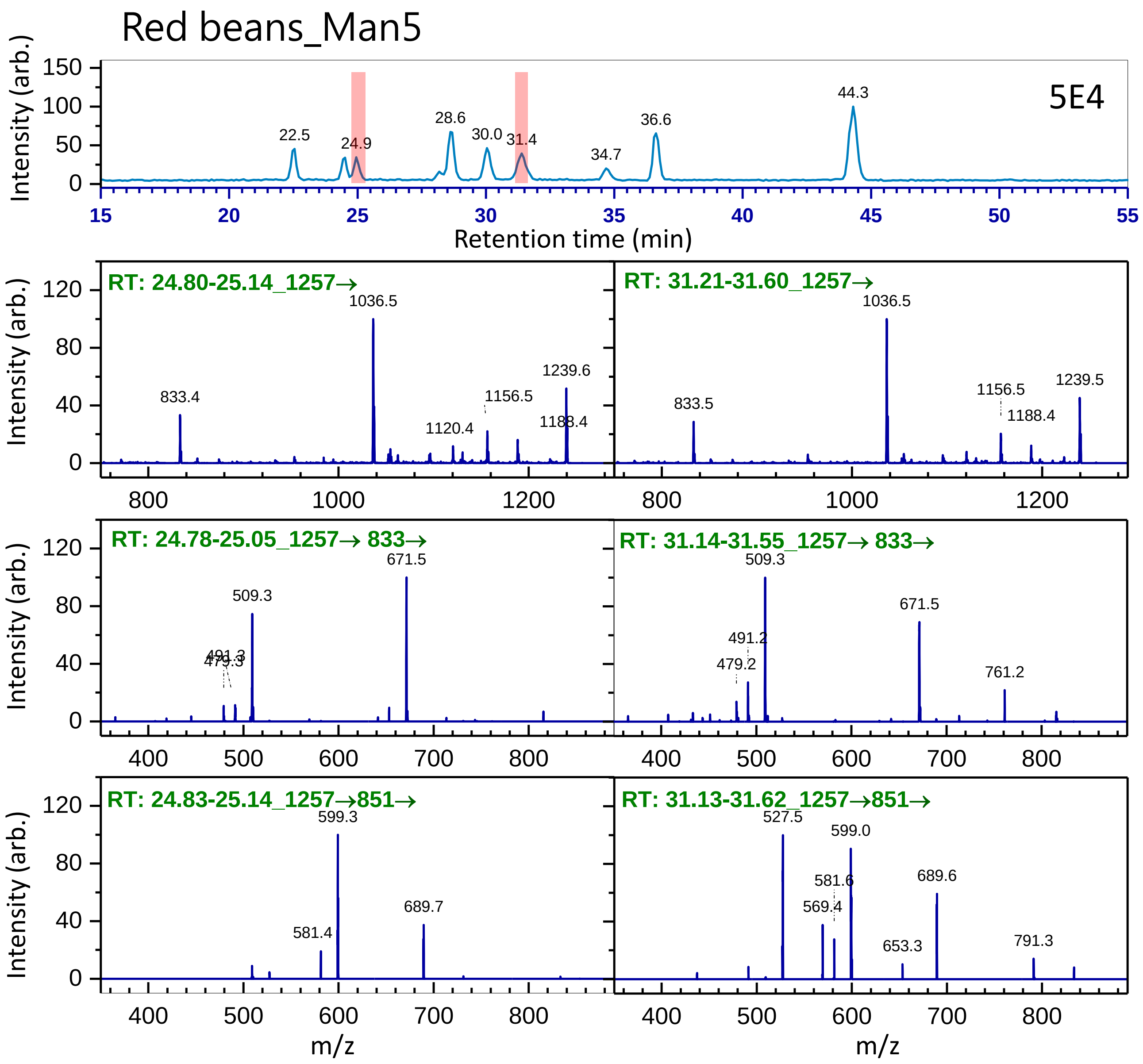

Figure S9. Chromatogram and MS^2 and MS^3 mass spectra of red bean $Man_5GlcNAC_2$.

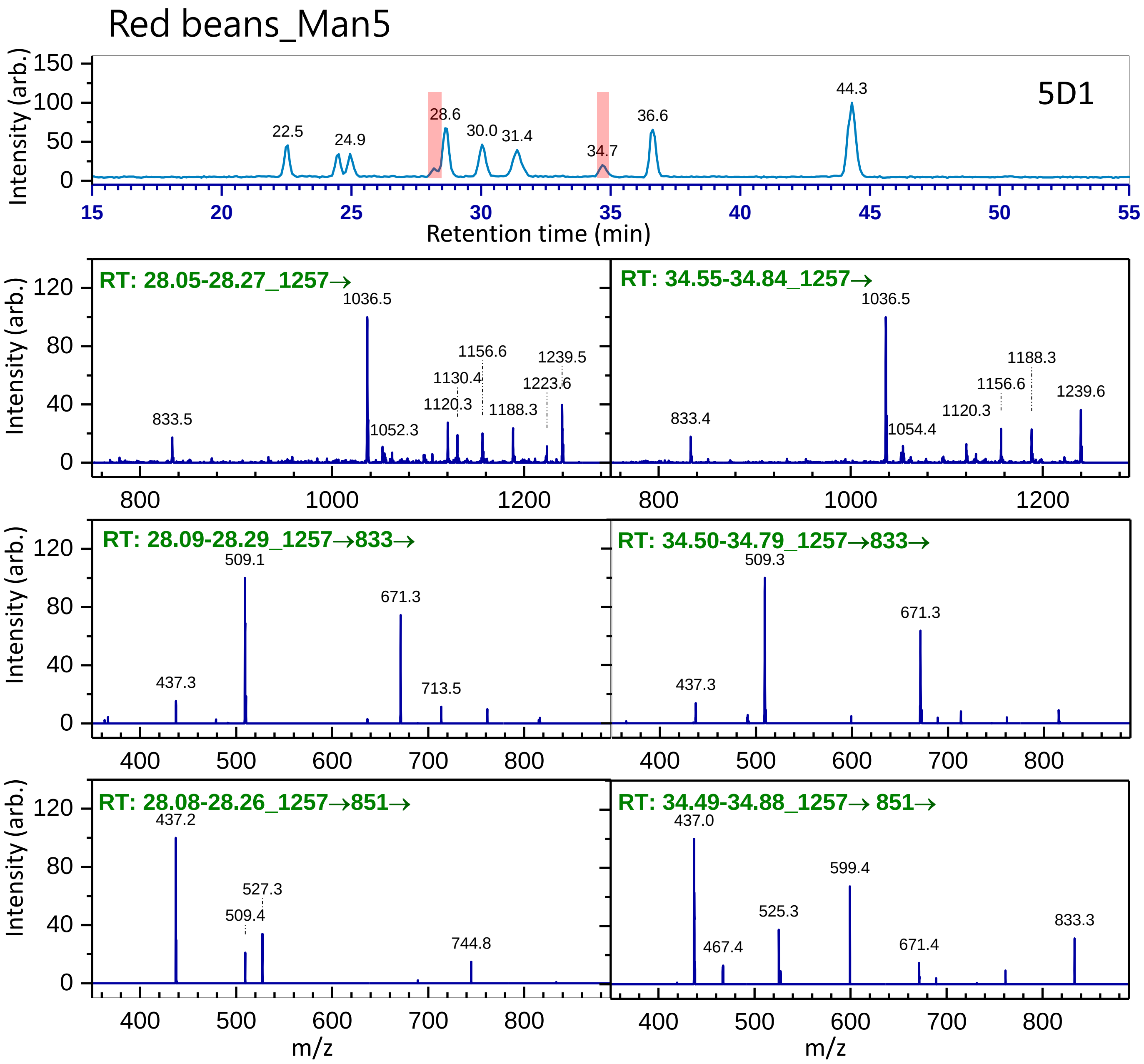

Figure S10. Chromatogram and MS^2 and MS^3 mass spectra of red bean $Man_5GlcNAC_2$.

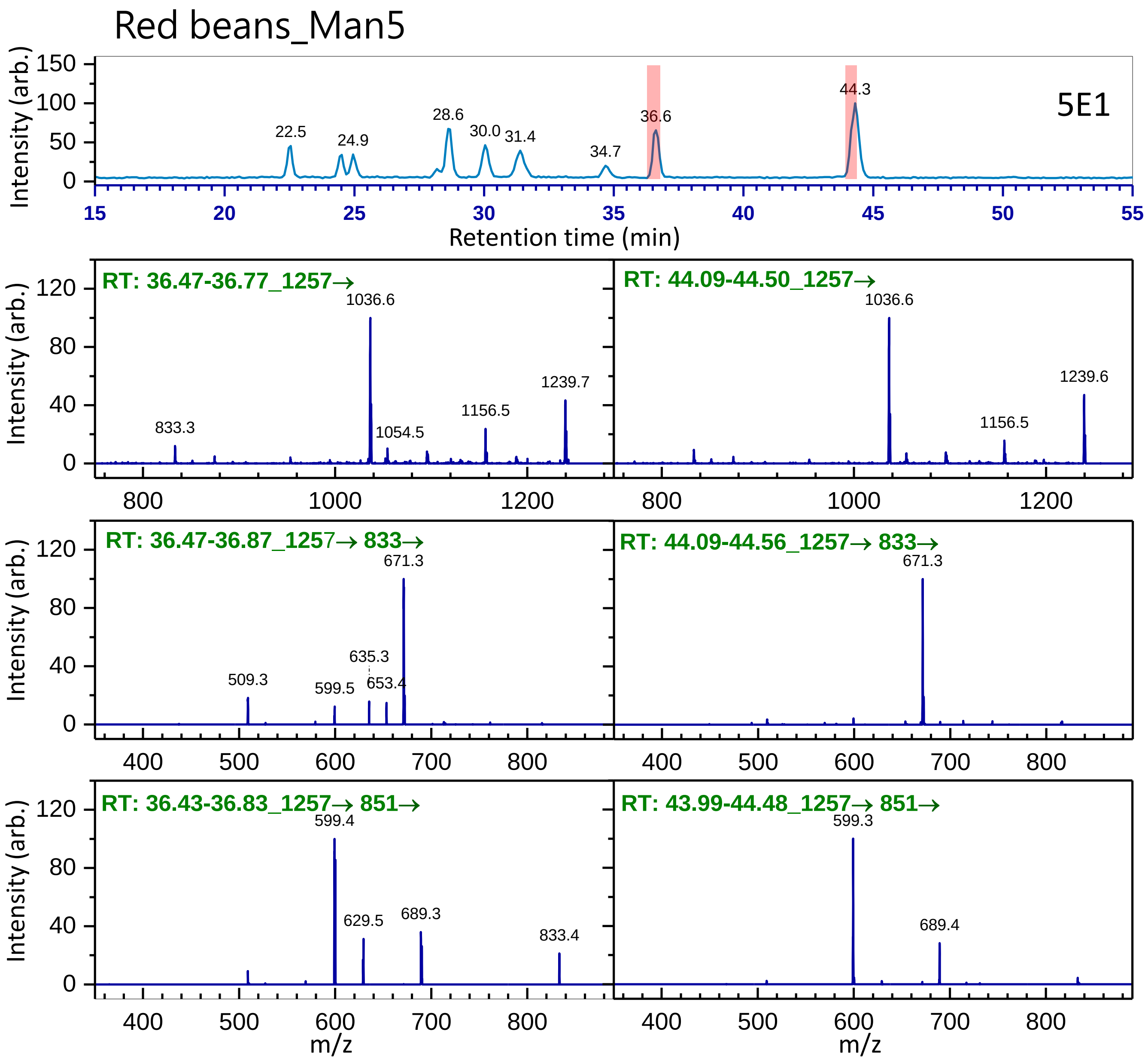

Figure S11. Chromatogram and MS^2 and MS^3 mass spectra of red bean $Man_5GlcNAC_2$.

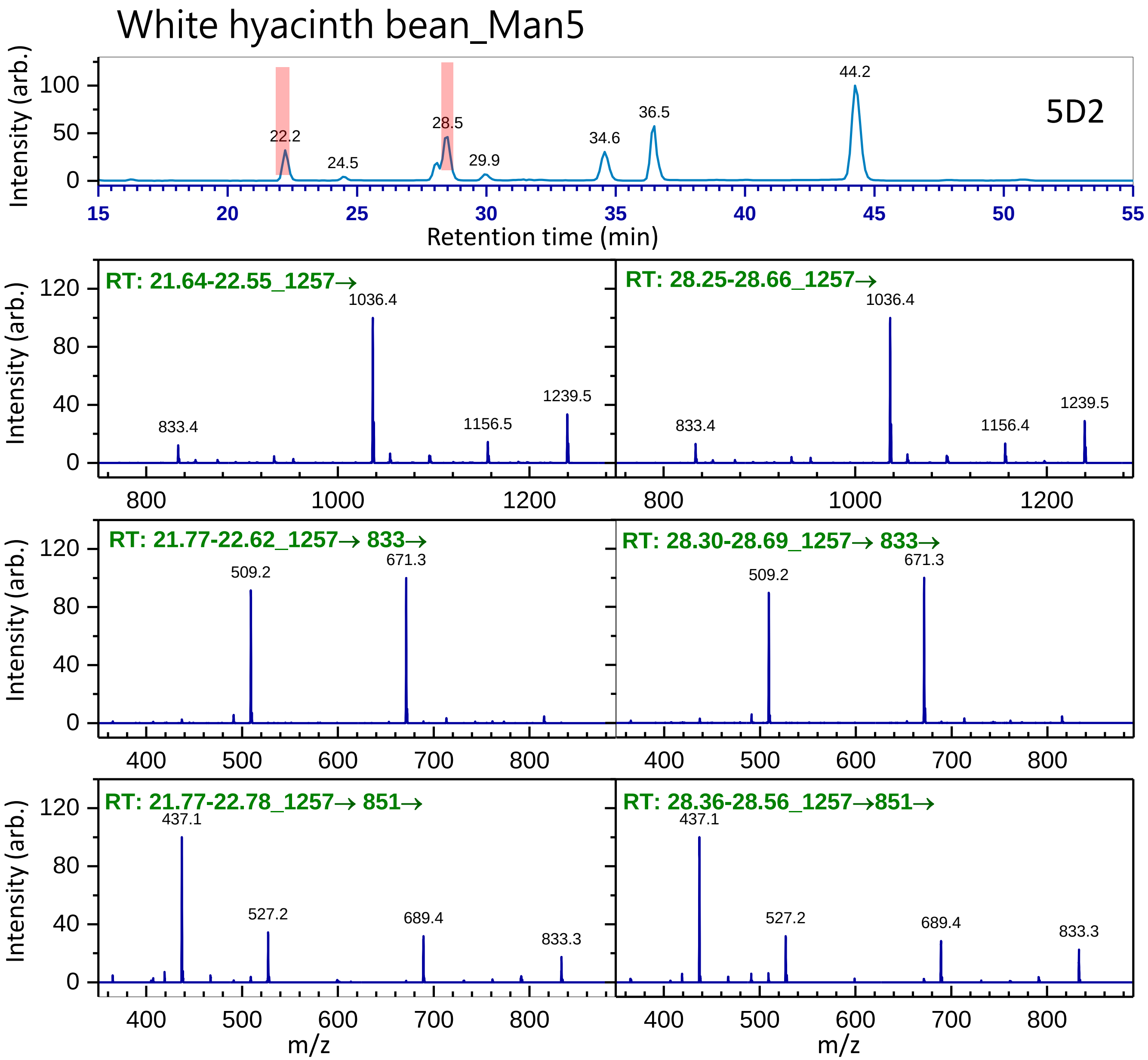

Figure S12. Chromatogram and MS^2 and MS^3 mass spectra of white hyacinth bean $Man_5GlcNAC_2$.

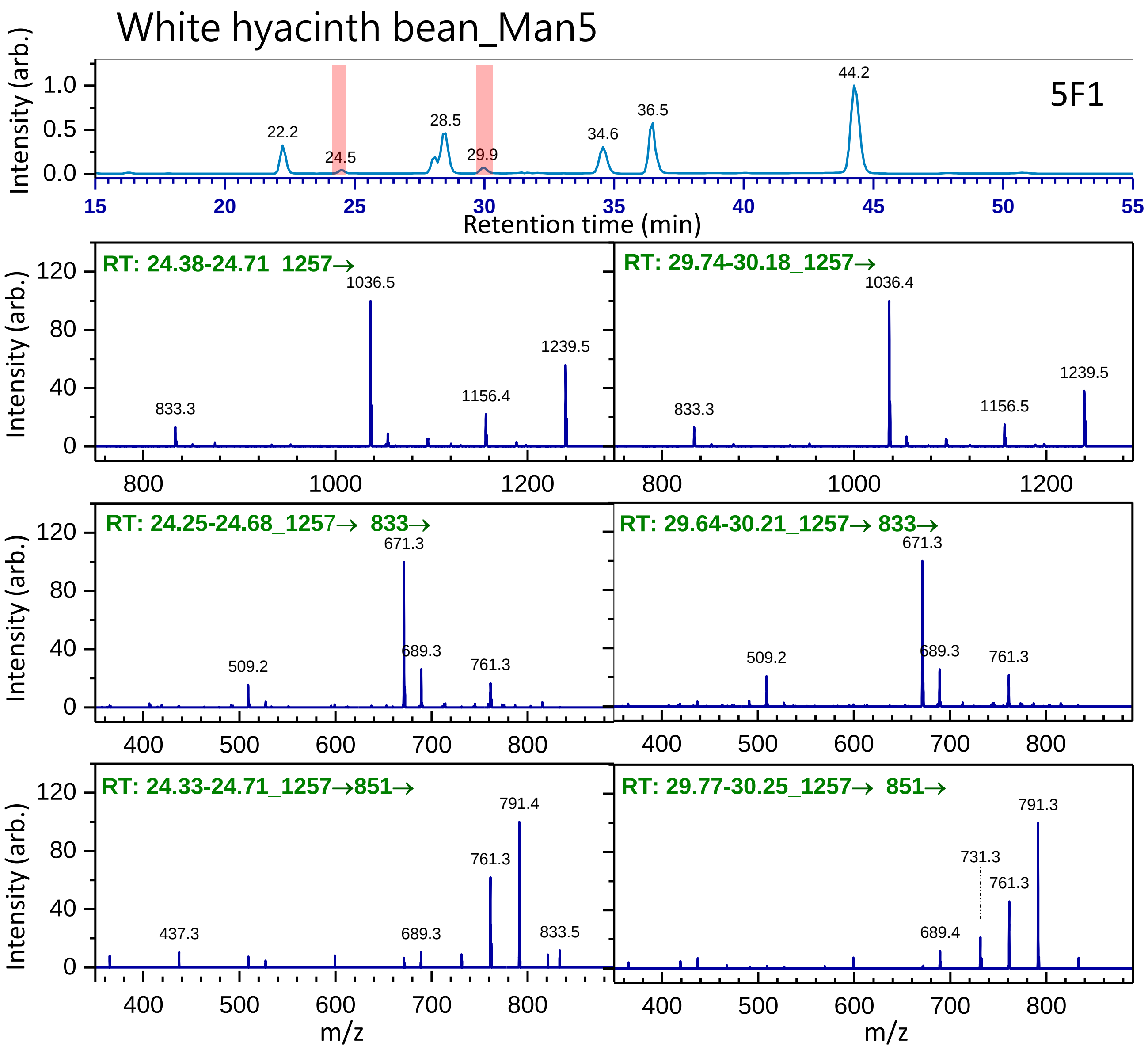

Figure S13. Chromatogram and MS^2 and MS^3 mass spectra of white hyacinth bean $Man_5GlcNAC_2$.

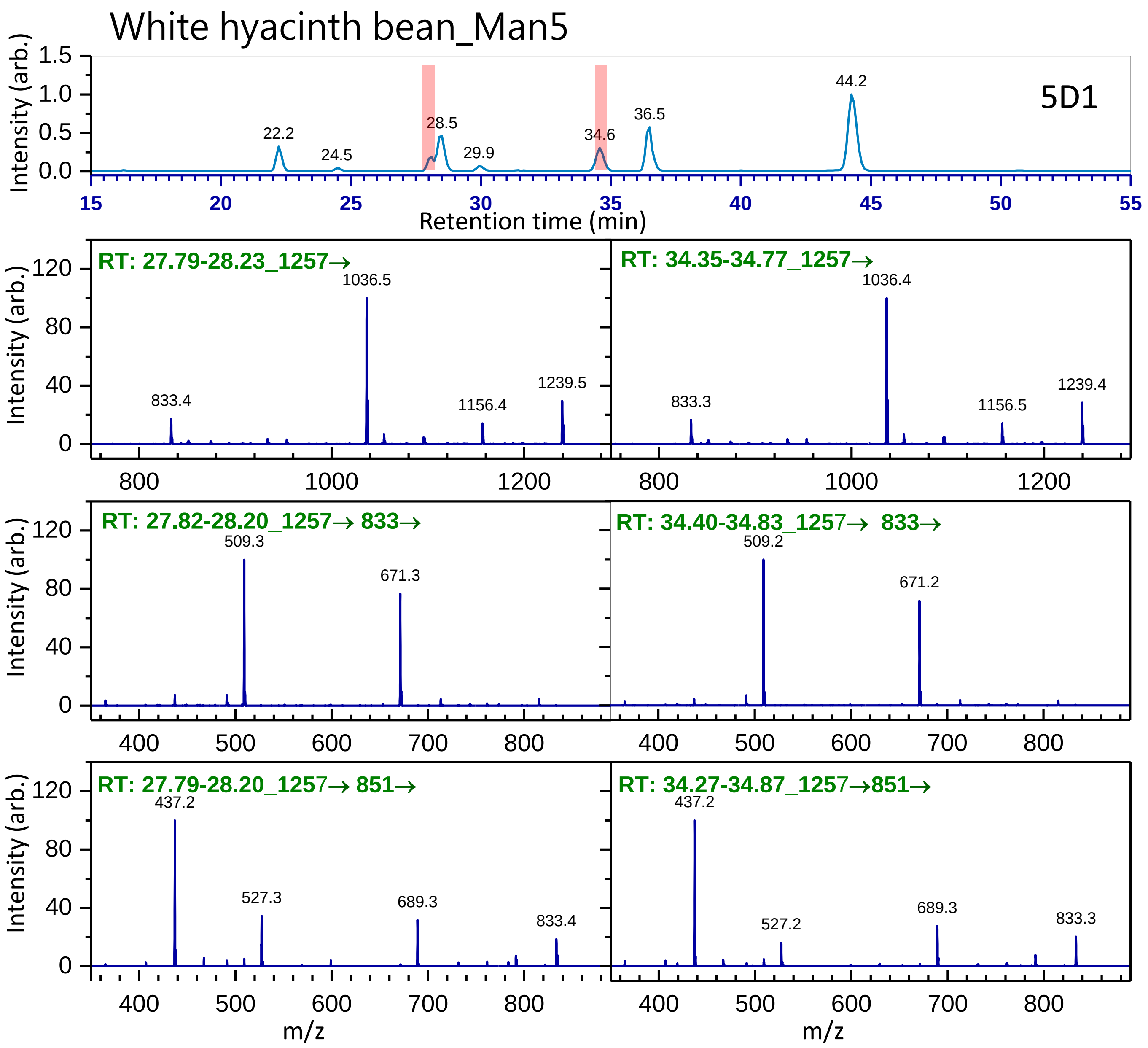

Figure S14. Chromatogram and MS^2 and MS^3 mass spectra of white hyacinth bean $Man_5GlcNAC_2$.

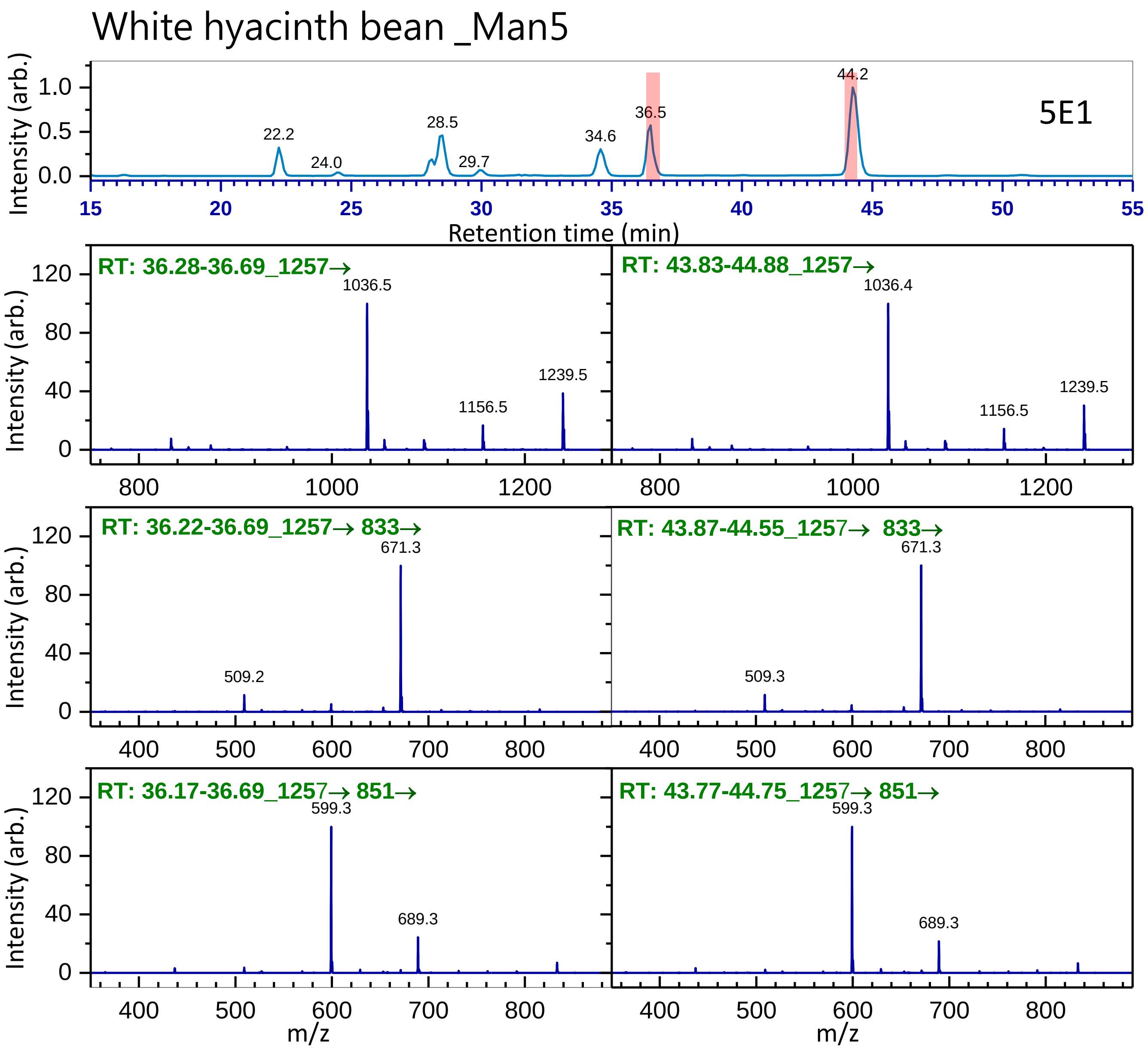

Figure S15. Chromatogram and MS^2 and MS^3 mass spectra of white hyacinth bean $Man_5GlcNAC_2$.

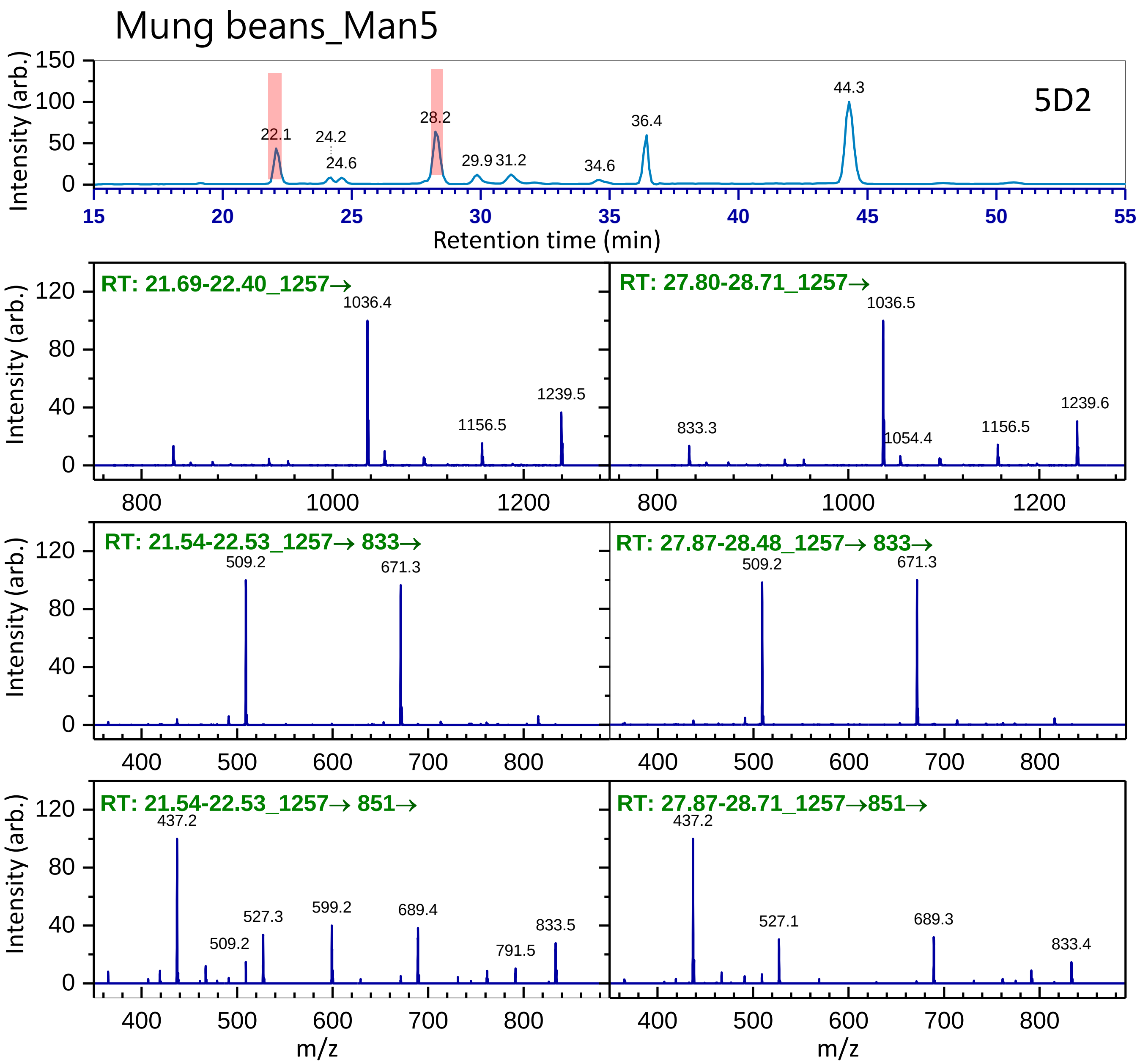

Figure S16. Chromatogram and MS^2 and MS^3 mass spectra of mung bean $Man_5GlcNAC_2$.

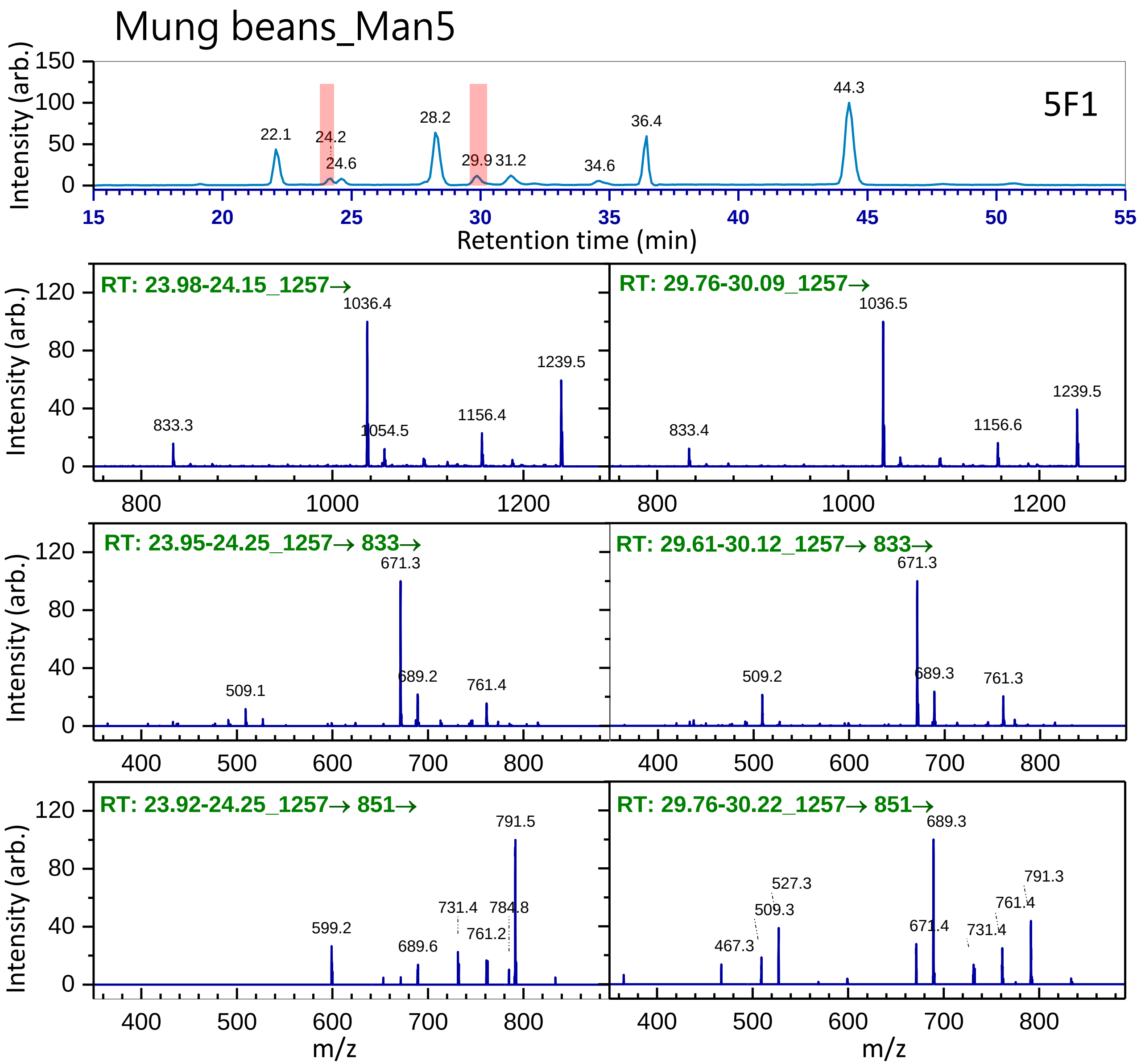

Figure S17. Chromatogram and MS^2 and MS^3 mass spectra of mung bean $Man_5GlcNAC_2$.

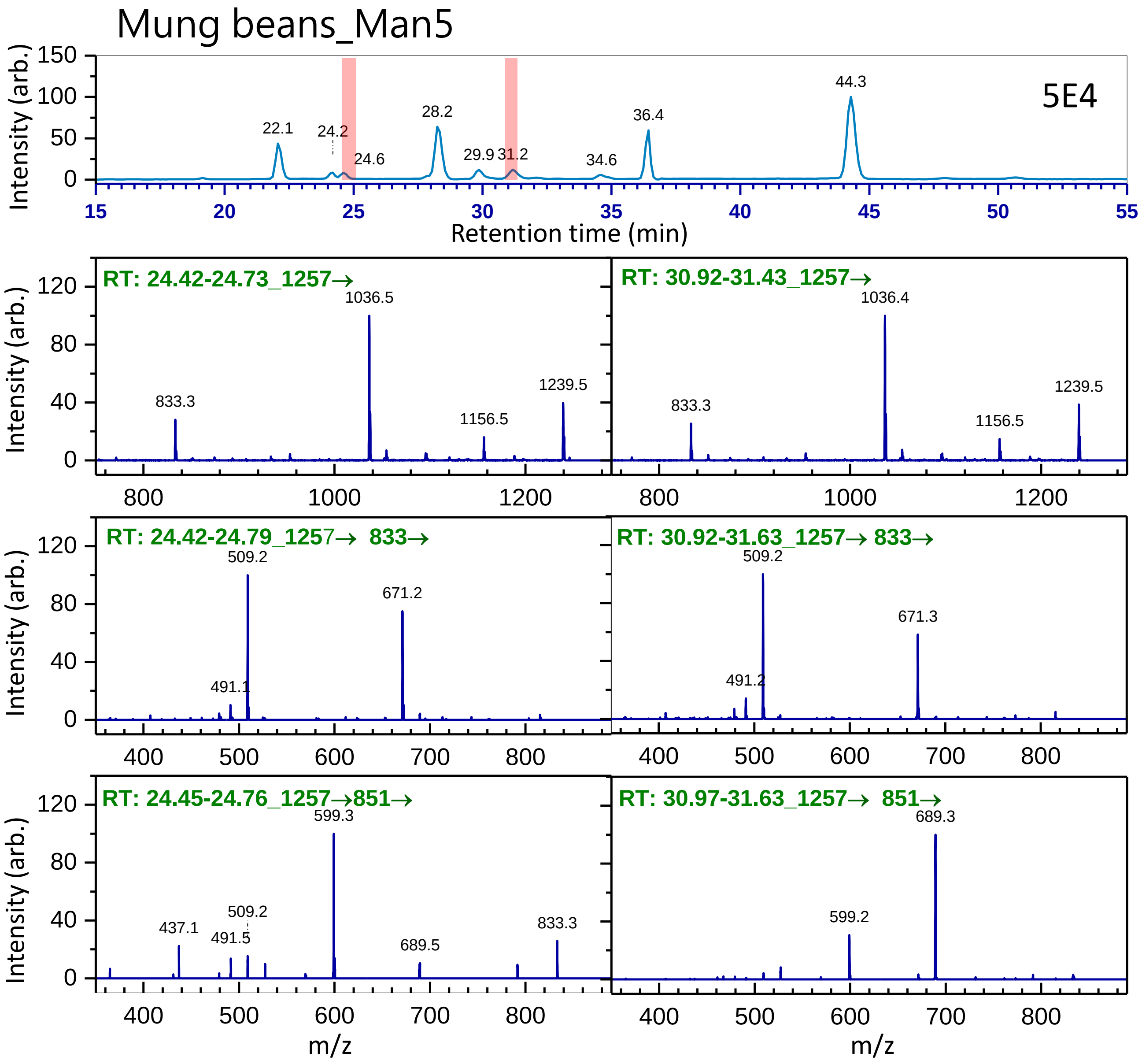

Figure S18. Chromatogram and MS^2 and MS^3 mass spectra of mung bean $Man_5GlcNAC_2$.

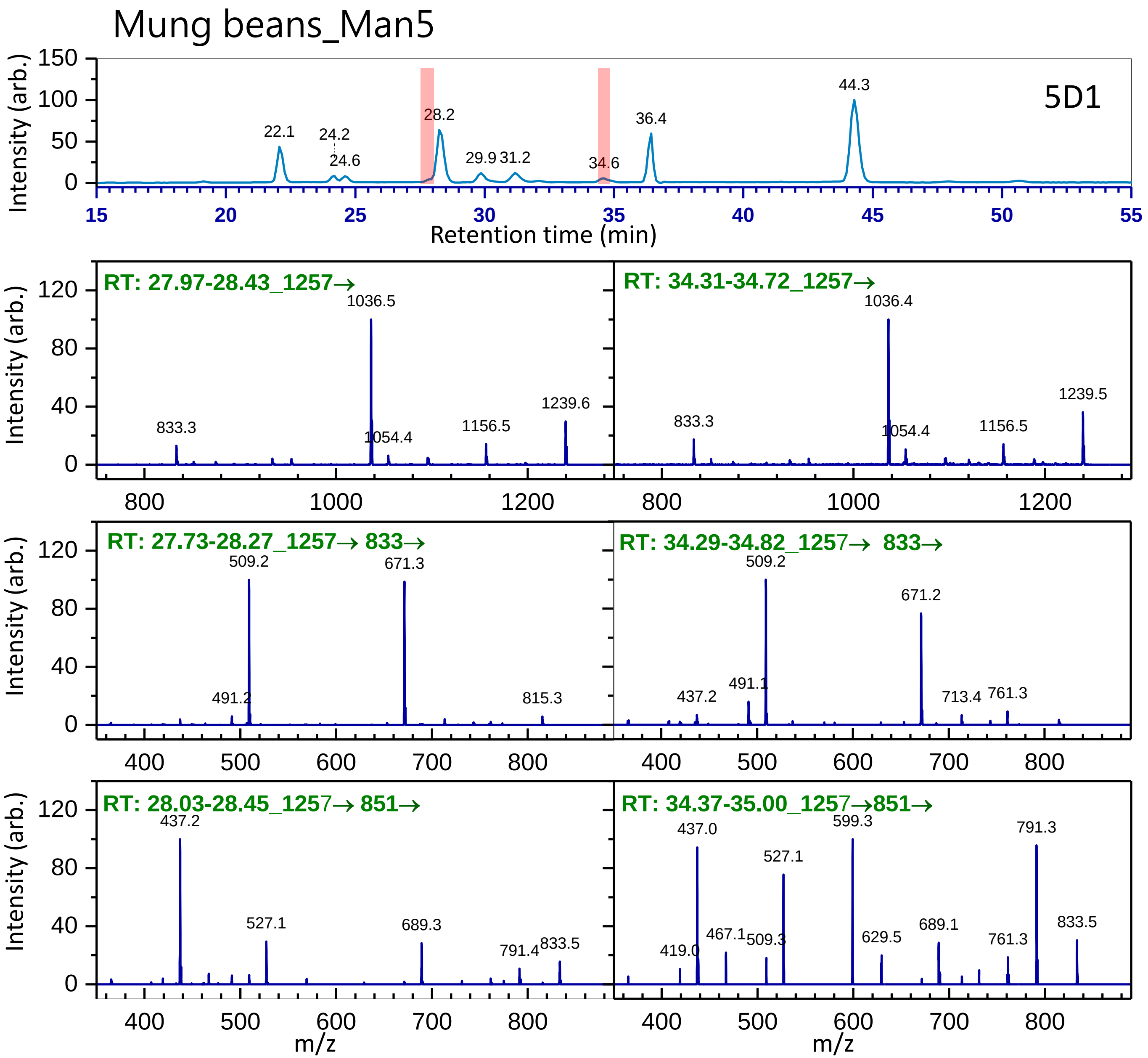

Figure S19. Chromatogram and MS^2 and MS^3 mass spectra of mung bean $Man_5GlcNAC_2$.

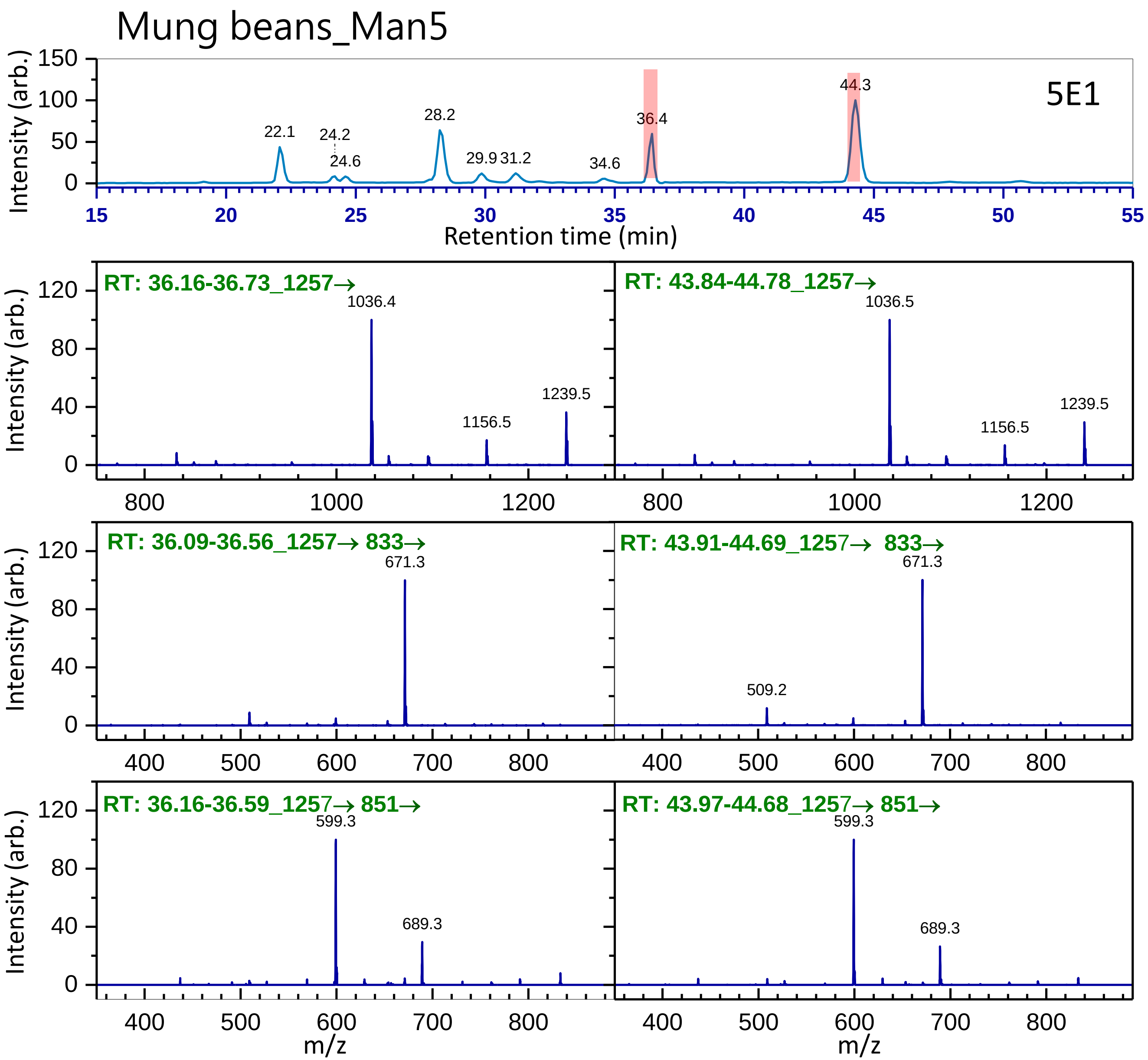

Figure S20. Chromatogram and MS^2 and MS^3 mass spectra of mung bean $Man_5GlcNAC_2$.

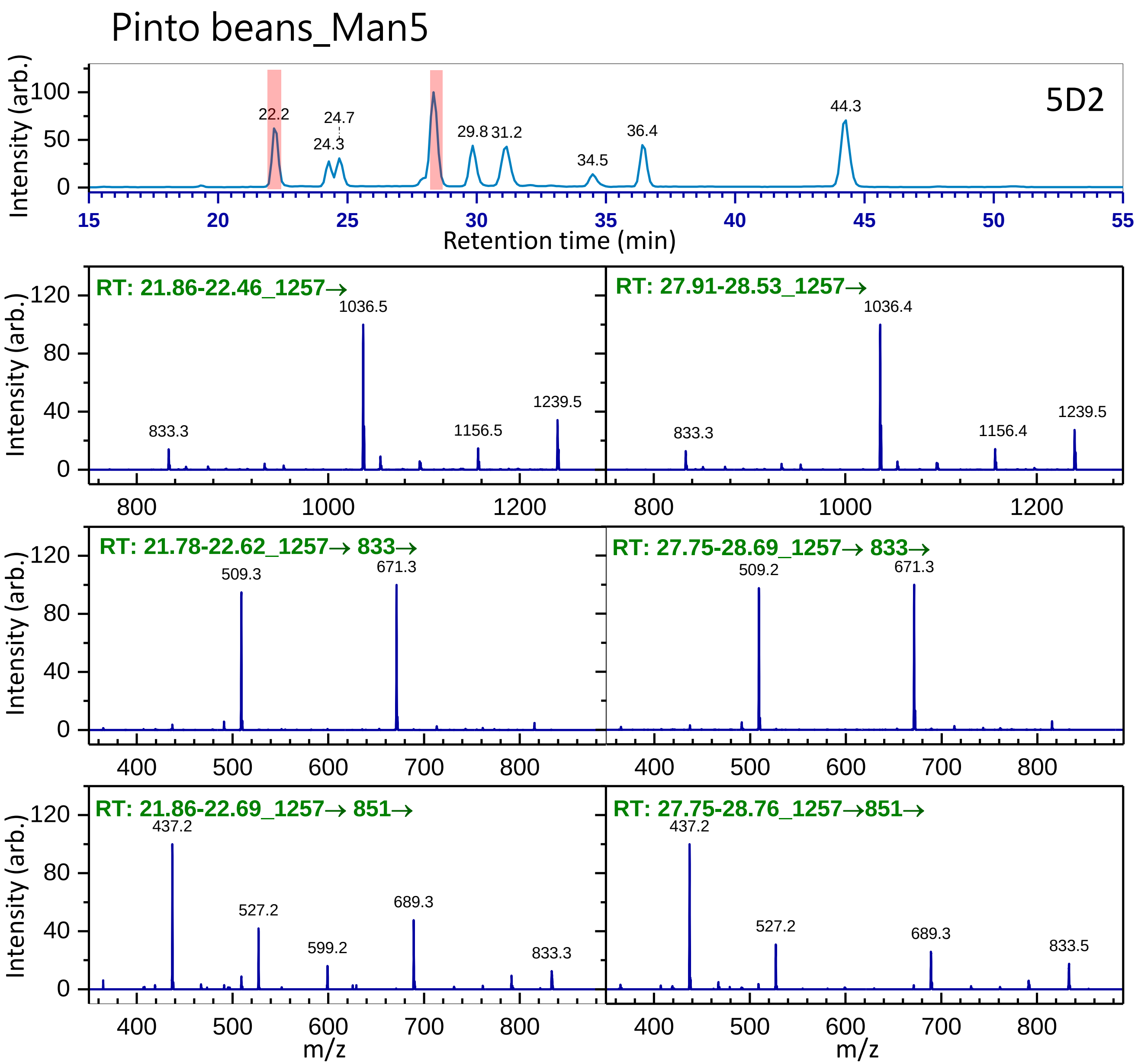

Figure S21. Chromatogram and MS^2 and MS^3 mass spectra of pinto bean $Man_5GlcNAC_2$.

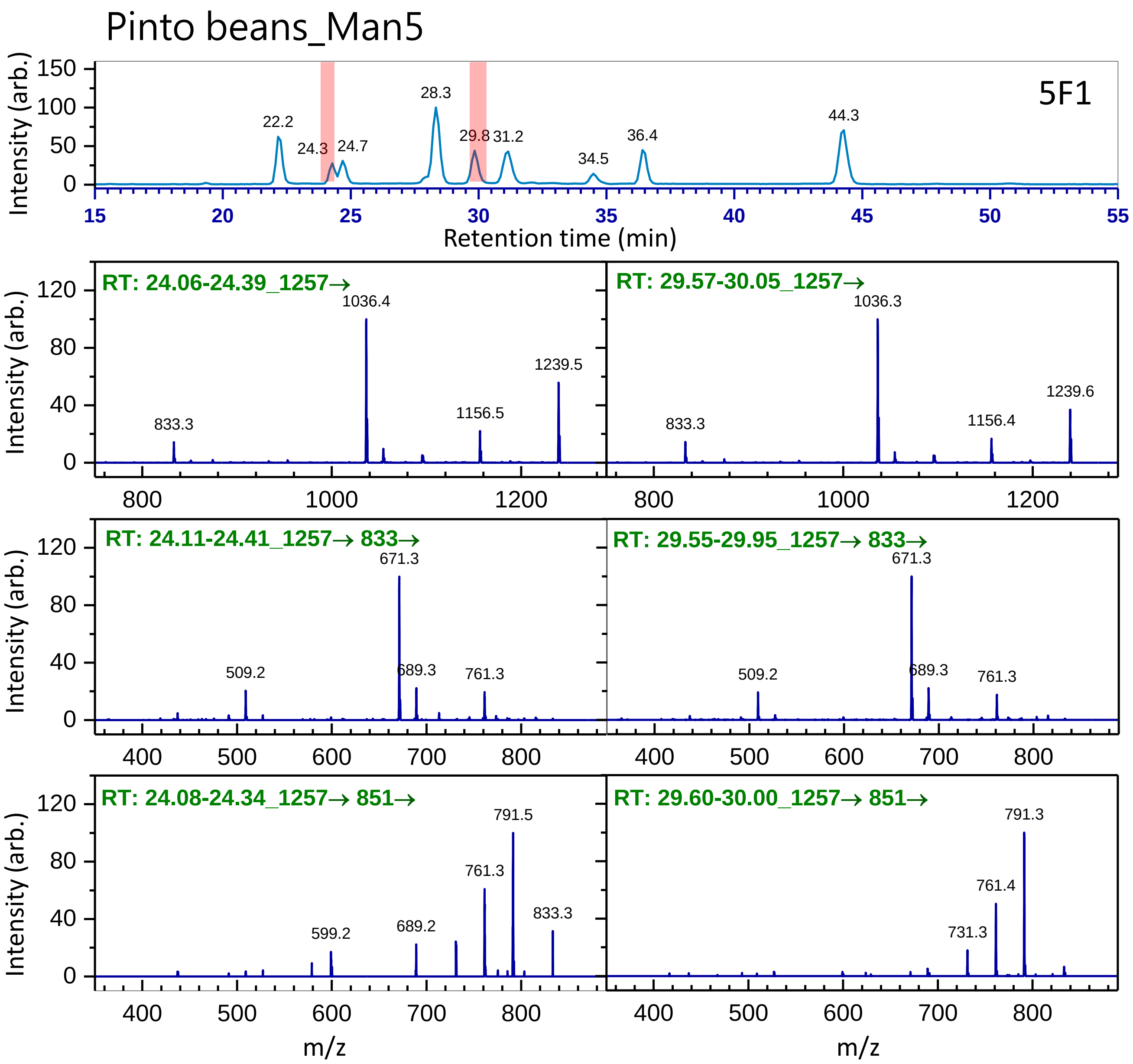

Figure S22. Chromatogram and MS^2 and MS^3 mass spectra of pinto bean $Man_5GlcNAC_2$.

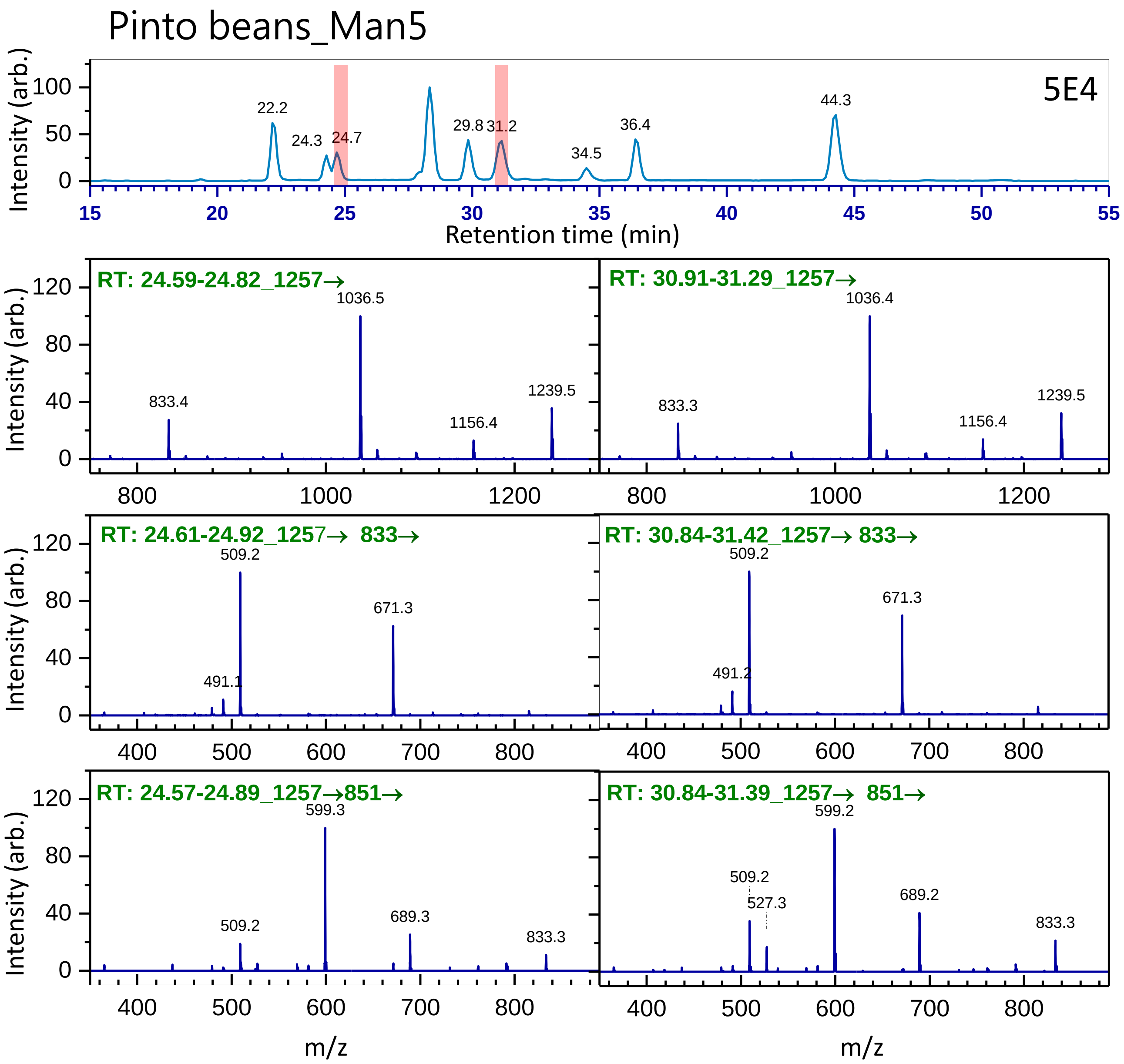

Figure S23. Chromatogram and MS^2 and MS^3 mass spectra of pinto bean $Man_5GlcNAC_2$.

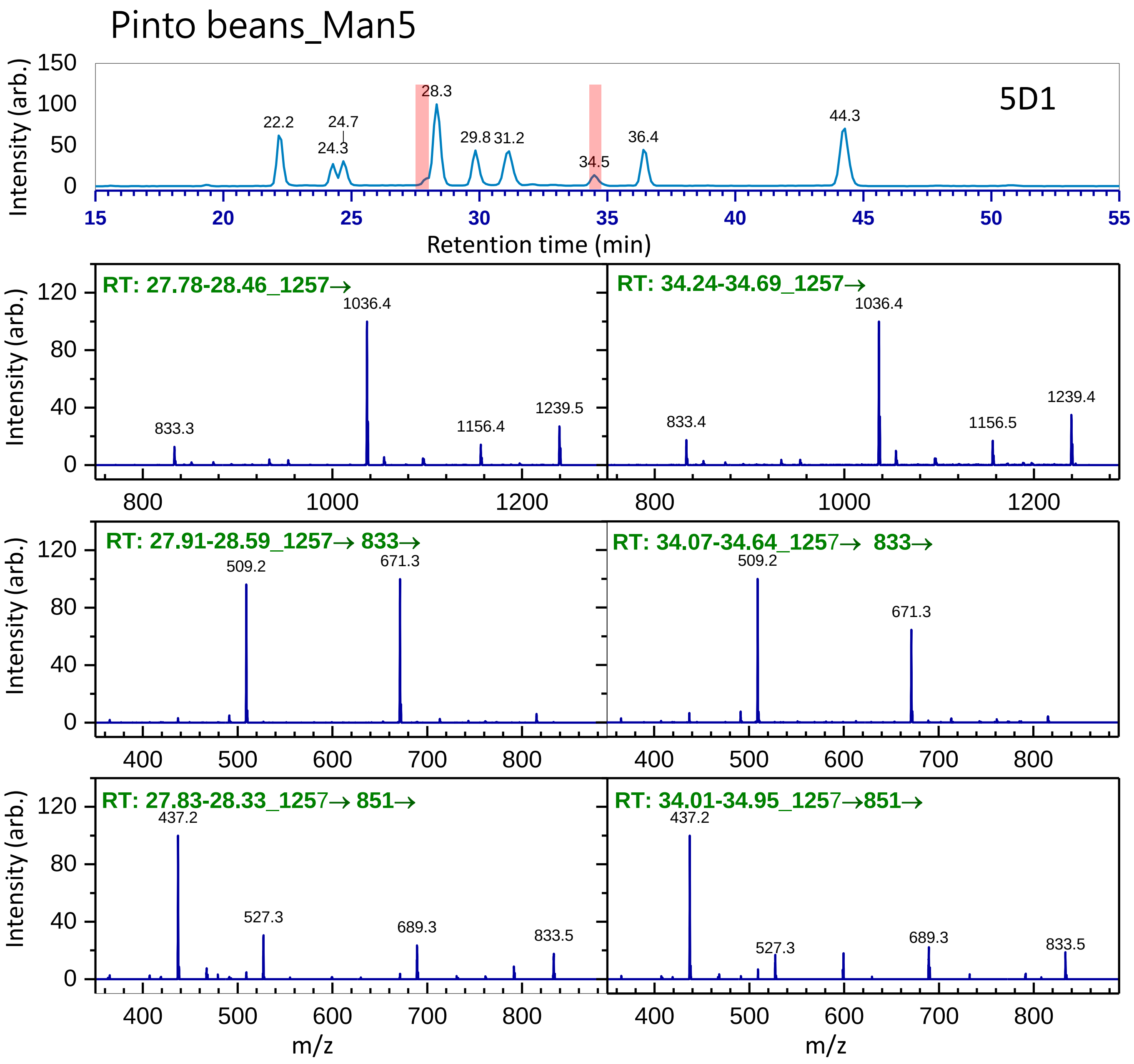

Figure S24. Chromatogram and MS^2 and MS^3 mass spectra of pinto bean $Man_5GlcNAC_2$.

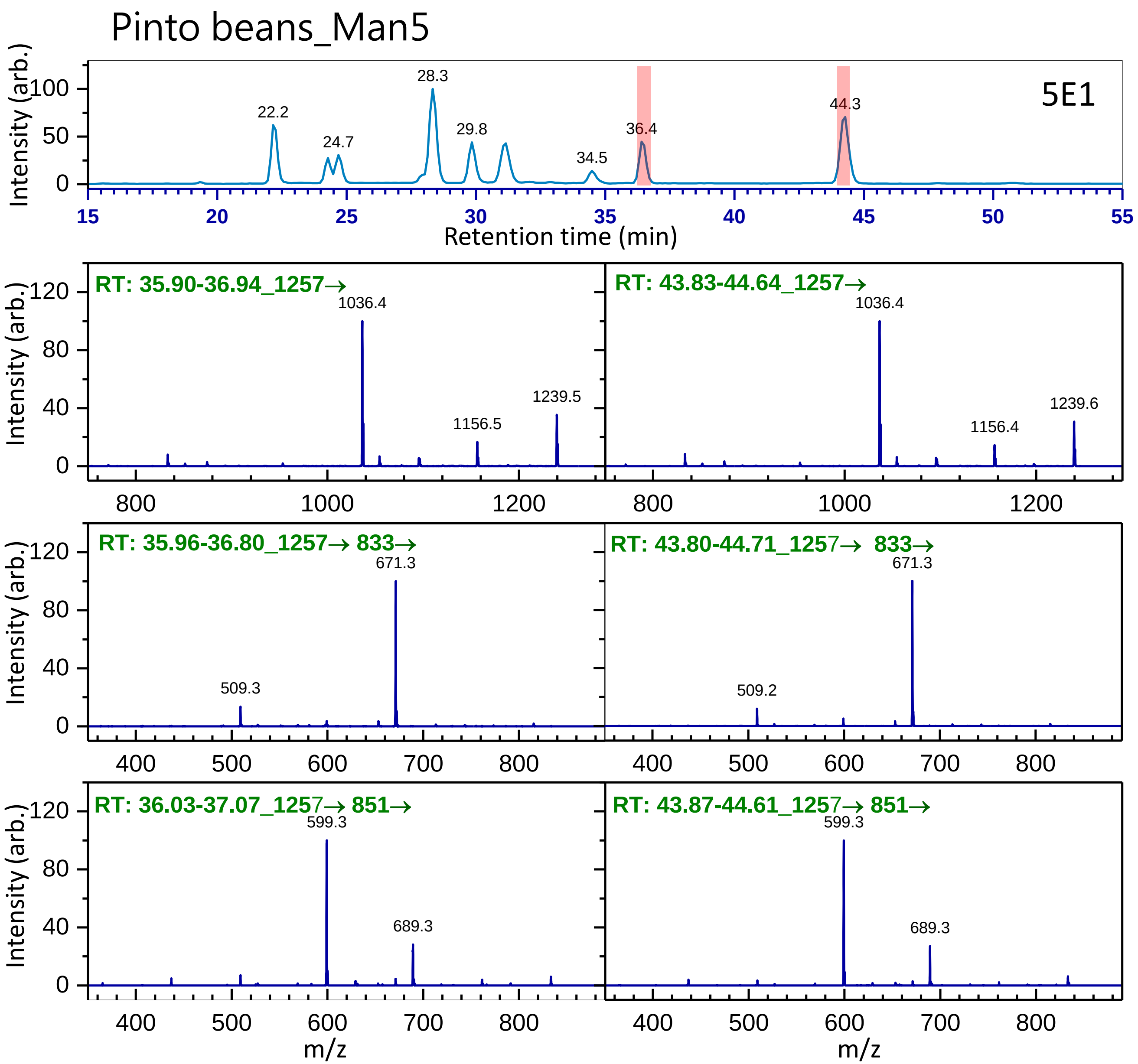

Figure S25. Chromatogram and MS^2 and MS^3 mass spectra of pinto bean $Man_5GlcNAC_2$.

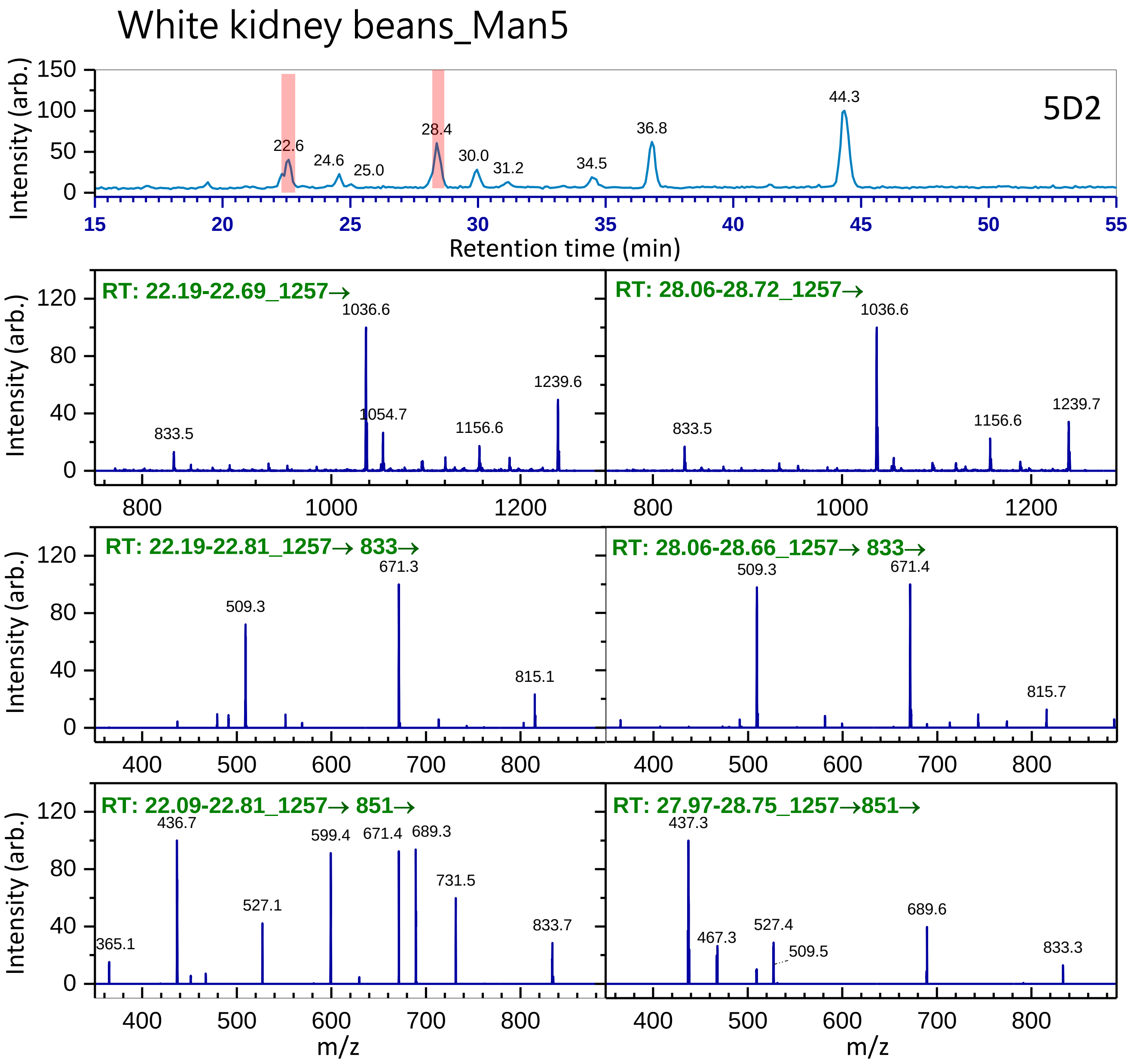

Figure S26. Chromatogram and MS^2 and MS^3 mass spectra of white kidney bean $Man_5GlcNAC_2$.

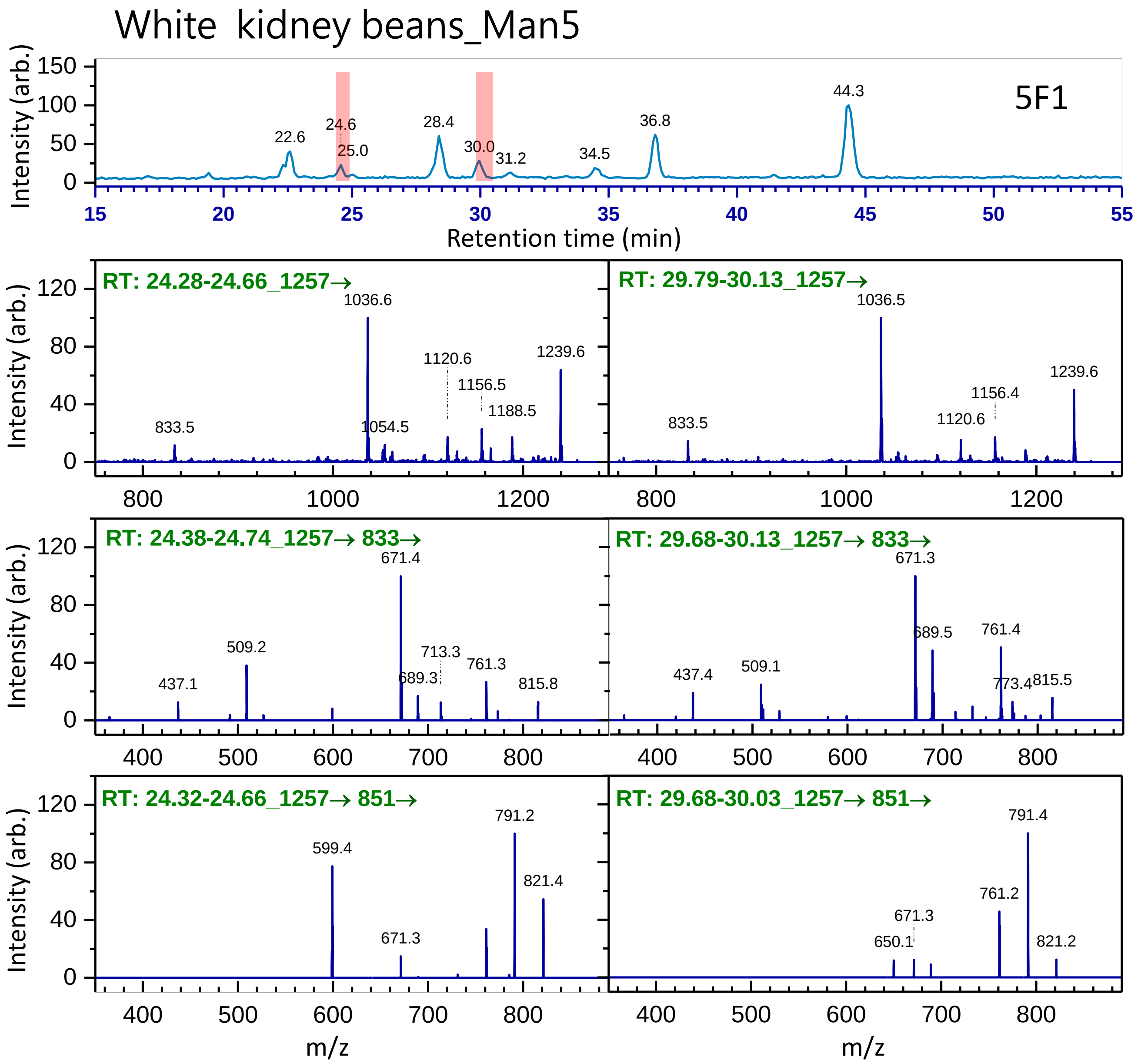

Figure S27. Chromatogram and MS^2 and MS^3 mass spectra of white kidney bean $Man_5GlcNAC_2$.

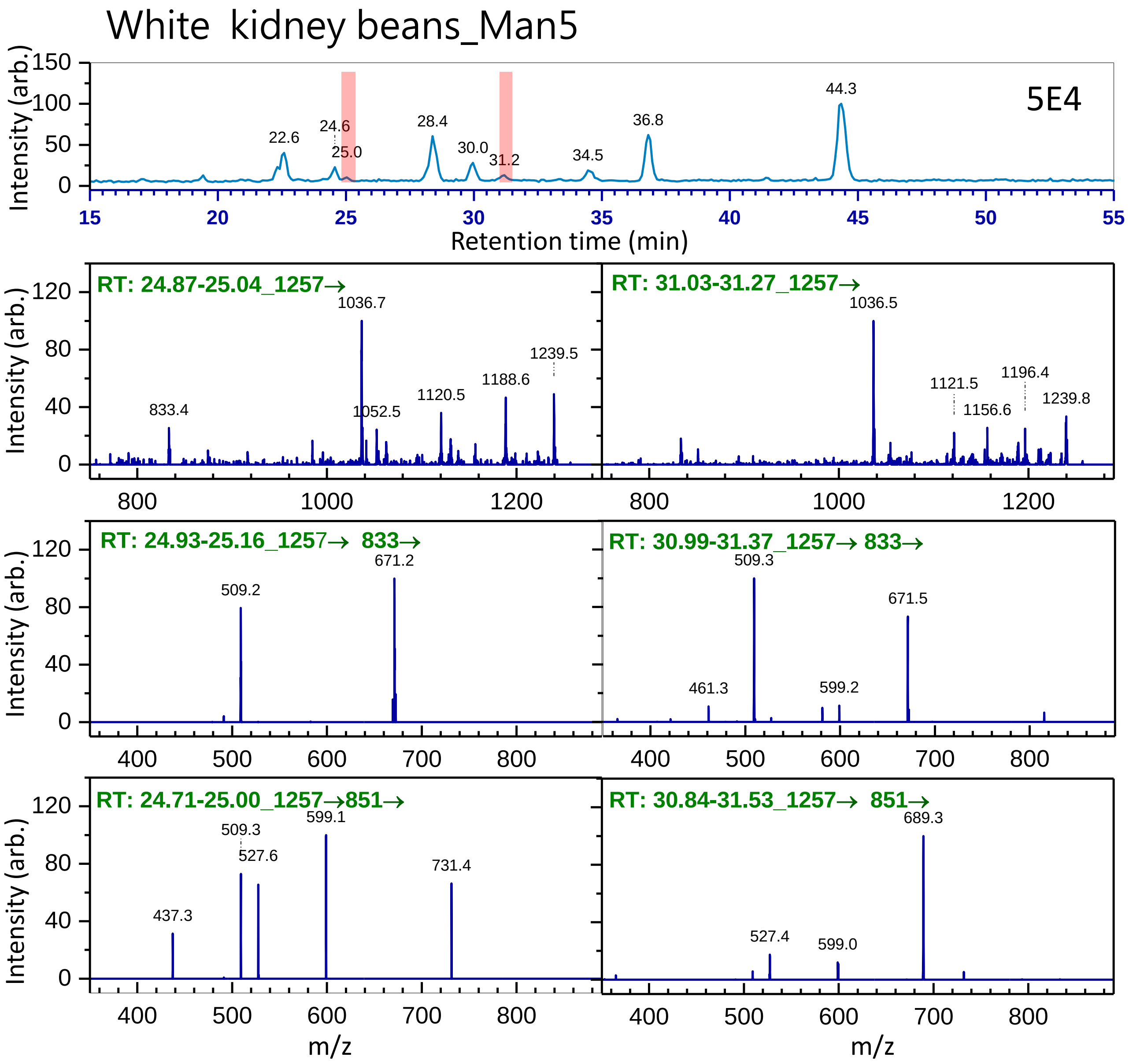

Figure S28. Chromatogram and MS^2 and MS^3 mass spectra of white kidney bean $Man_5GlcNAC_2$.

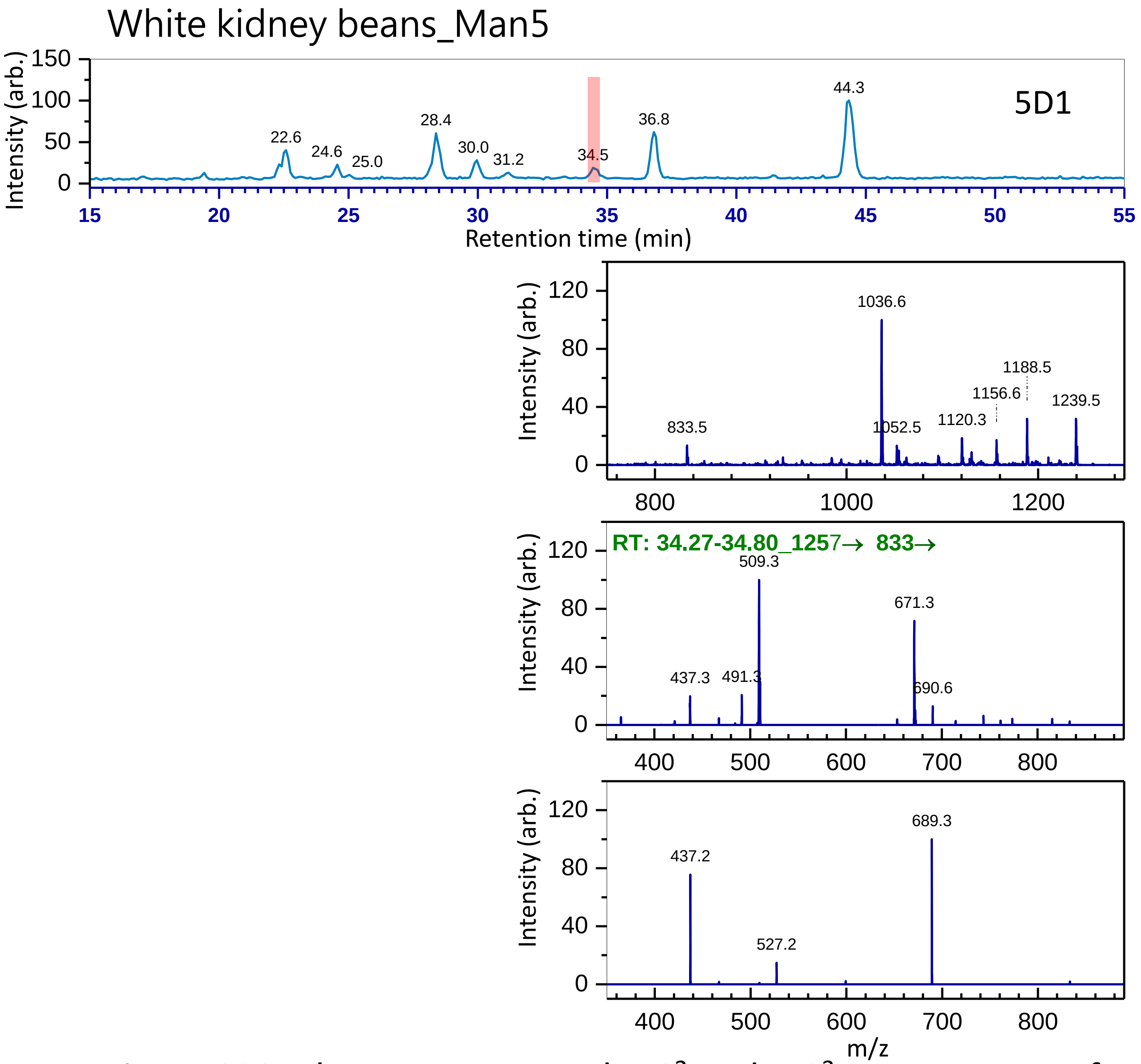

Figure S29. Chromatogram and MS^2 and MS^3 mass spectra of white kidney bean $Man_5GlcNAC_2$.

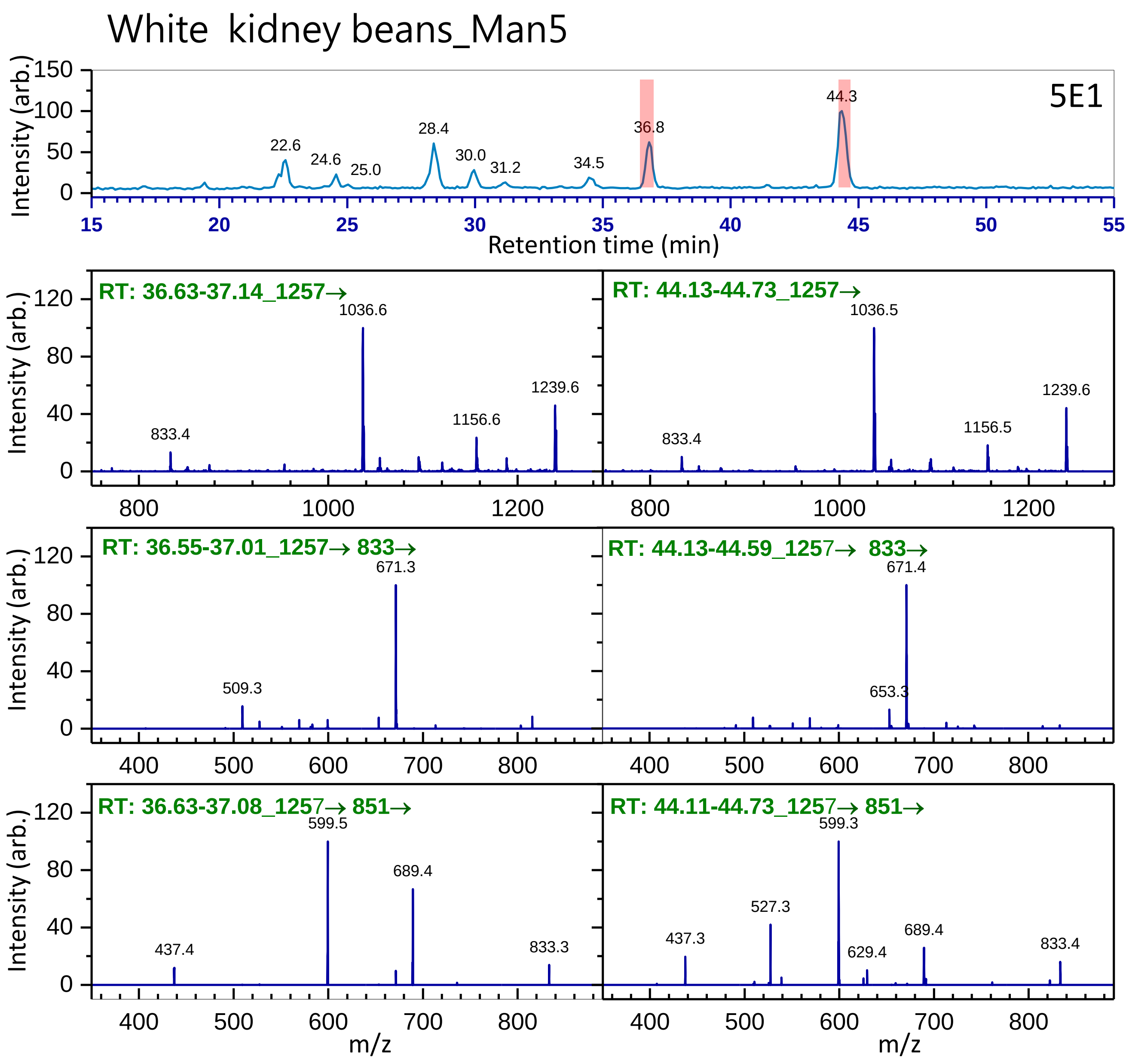

Figure S30. Chromatogram and MS^2 and MS^3 mass spectra of white kidney bean $Man_5GlcNAC_2$.

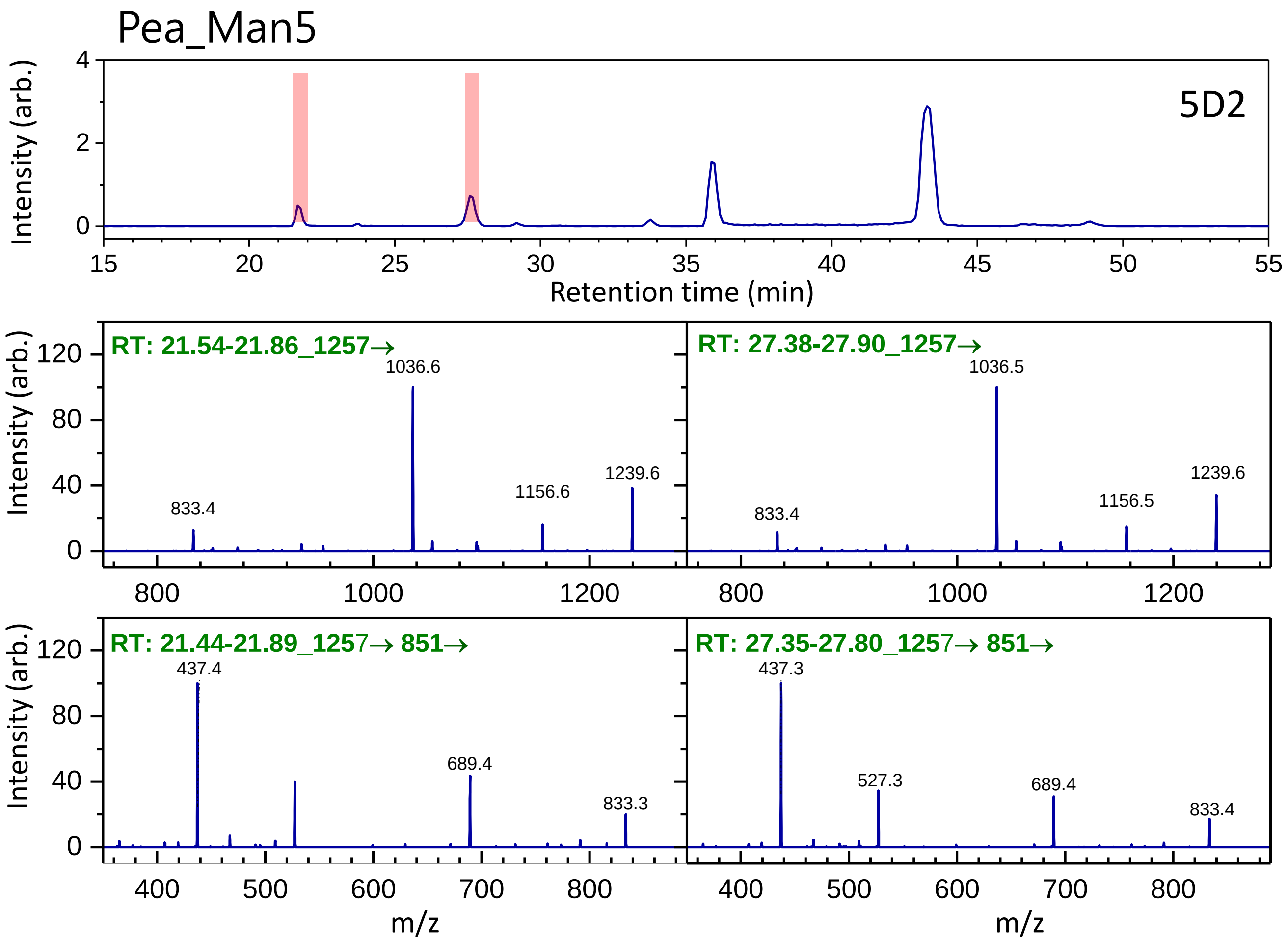

Figure S31. Chromatogram and MS^2 and MS^3 mass spectra of pea $Man_5GlcNAC_2$.

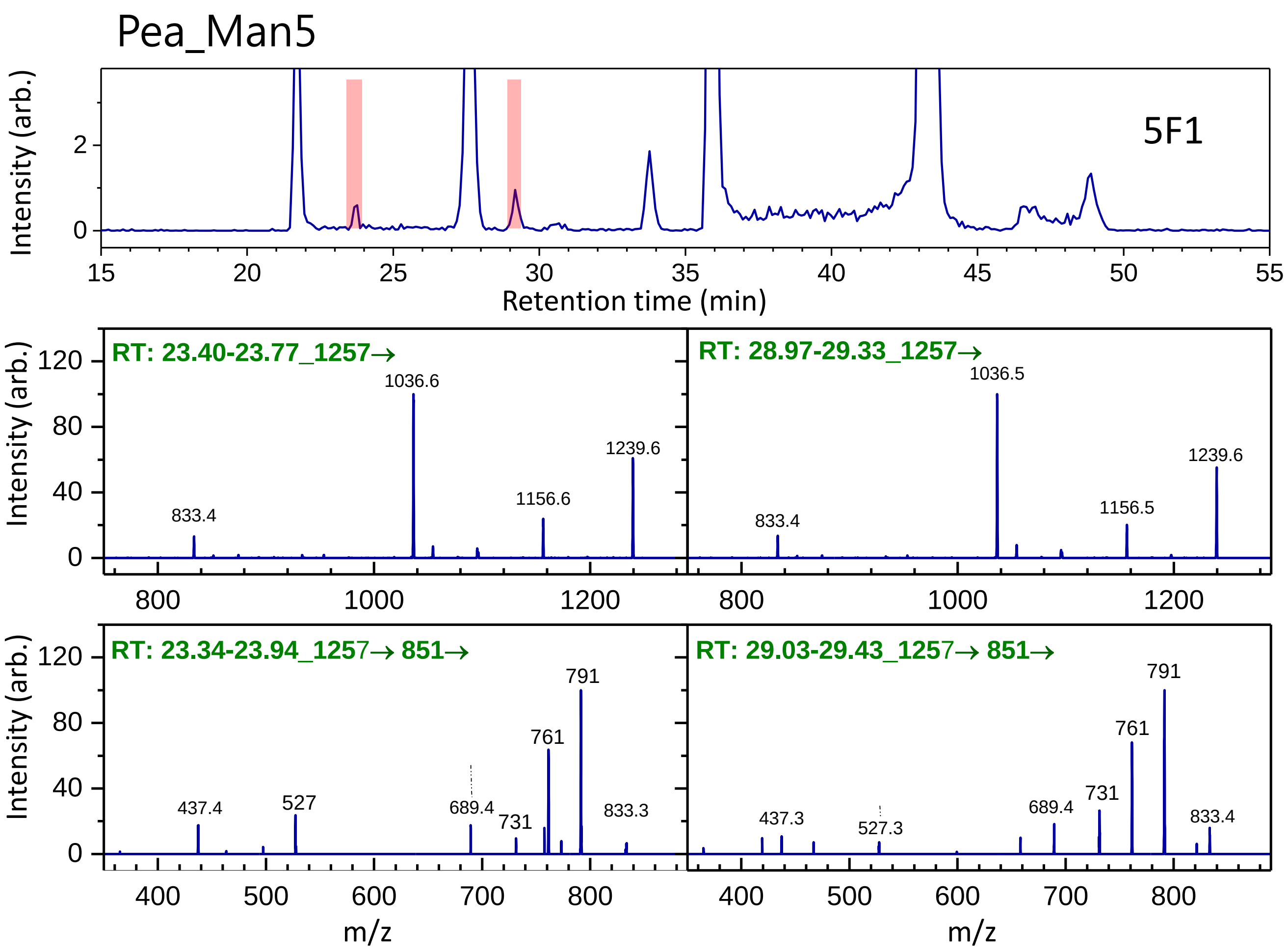

Figure S32. Chromatogram and MS^2 and MS^3 mass spectra of pea $Man_5GlcNAC_2$.

Pea_Man5

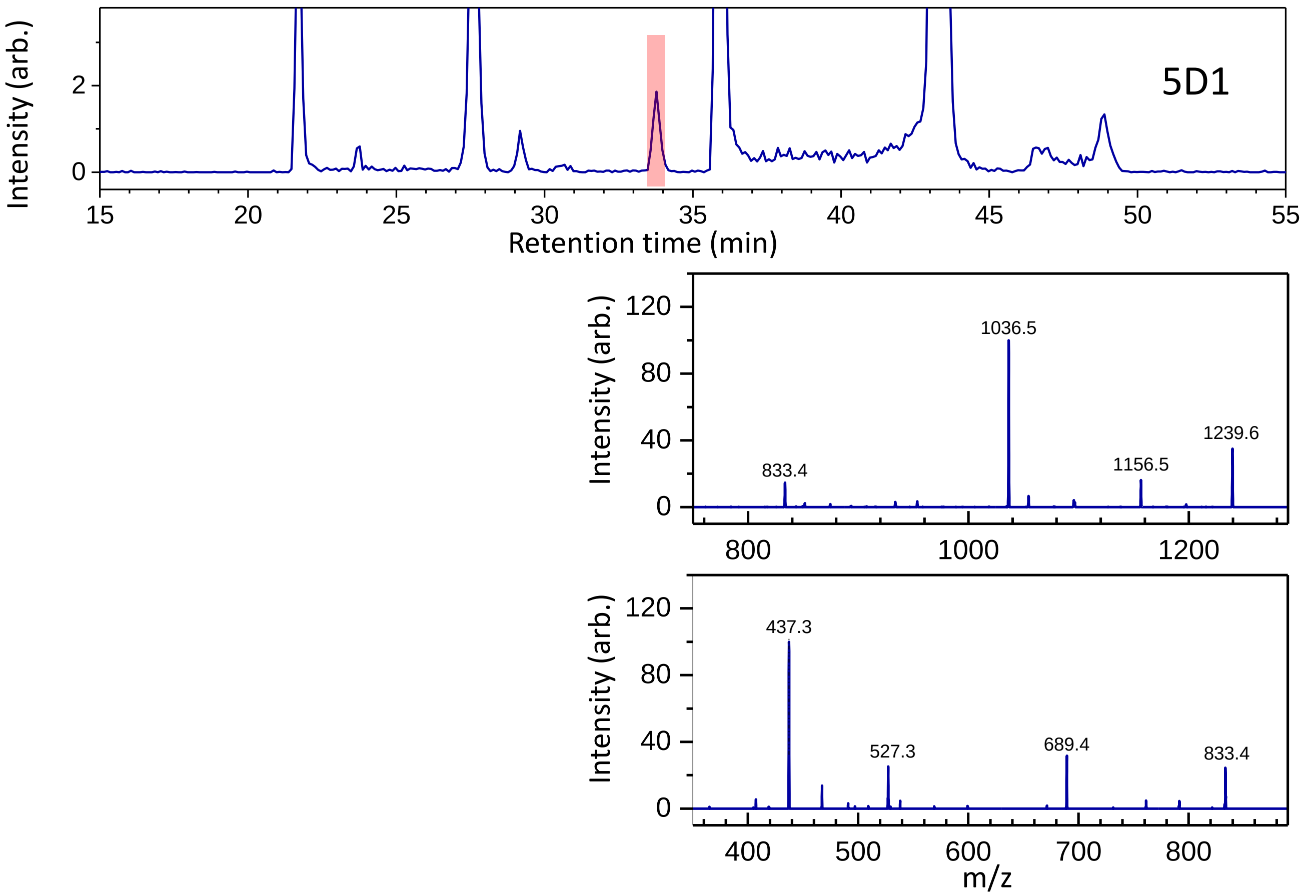

Figure S33. Chromatogram and MS^2 and MS^3 mass spectra of pea $Man_5GlcNAC_2$.

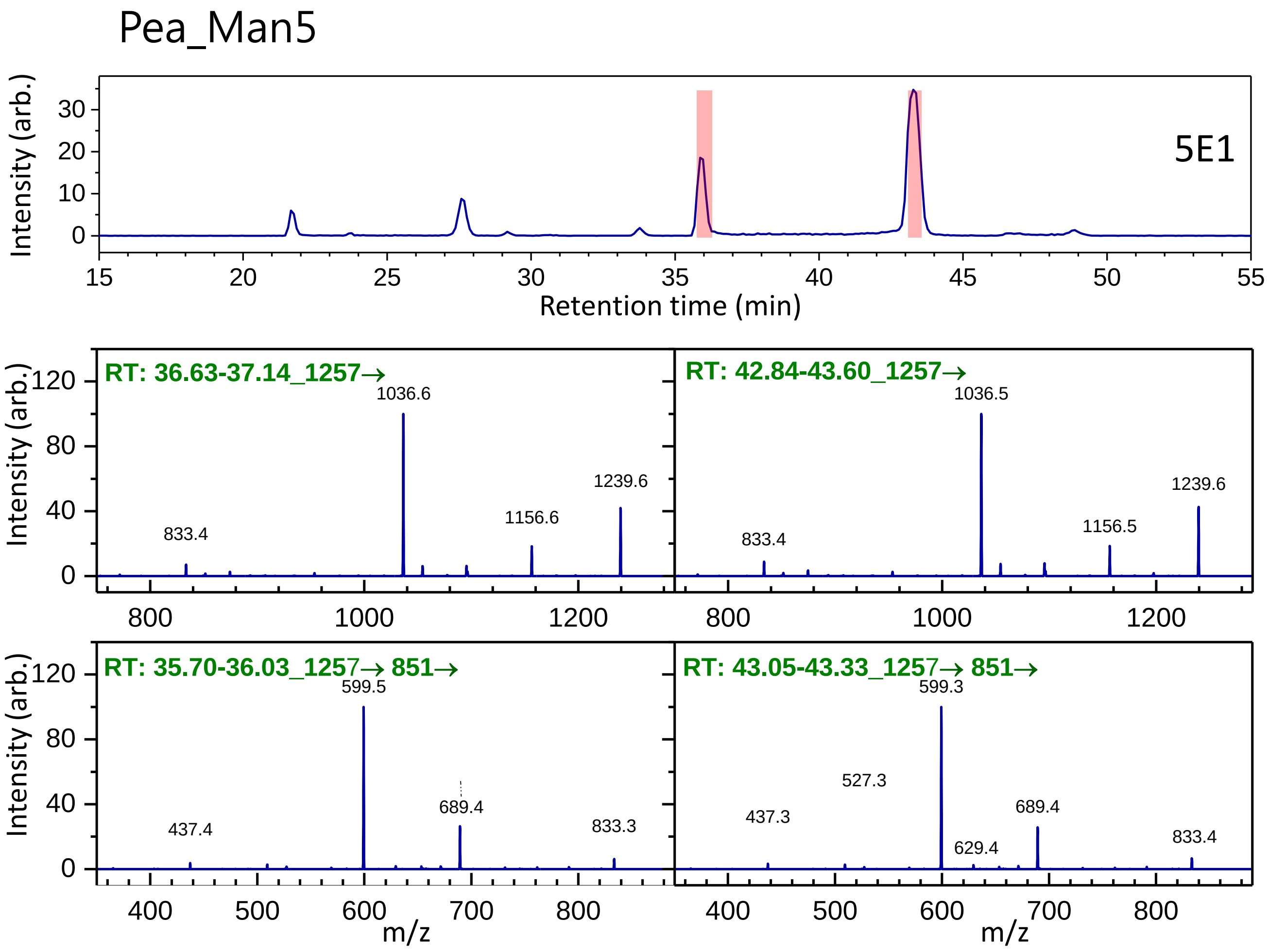

Figure S34. Chromatogram and MS^2 and MS^3 mass spectra of pea $Man_5GlcNAC_2$.

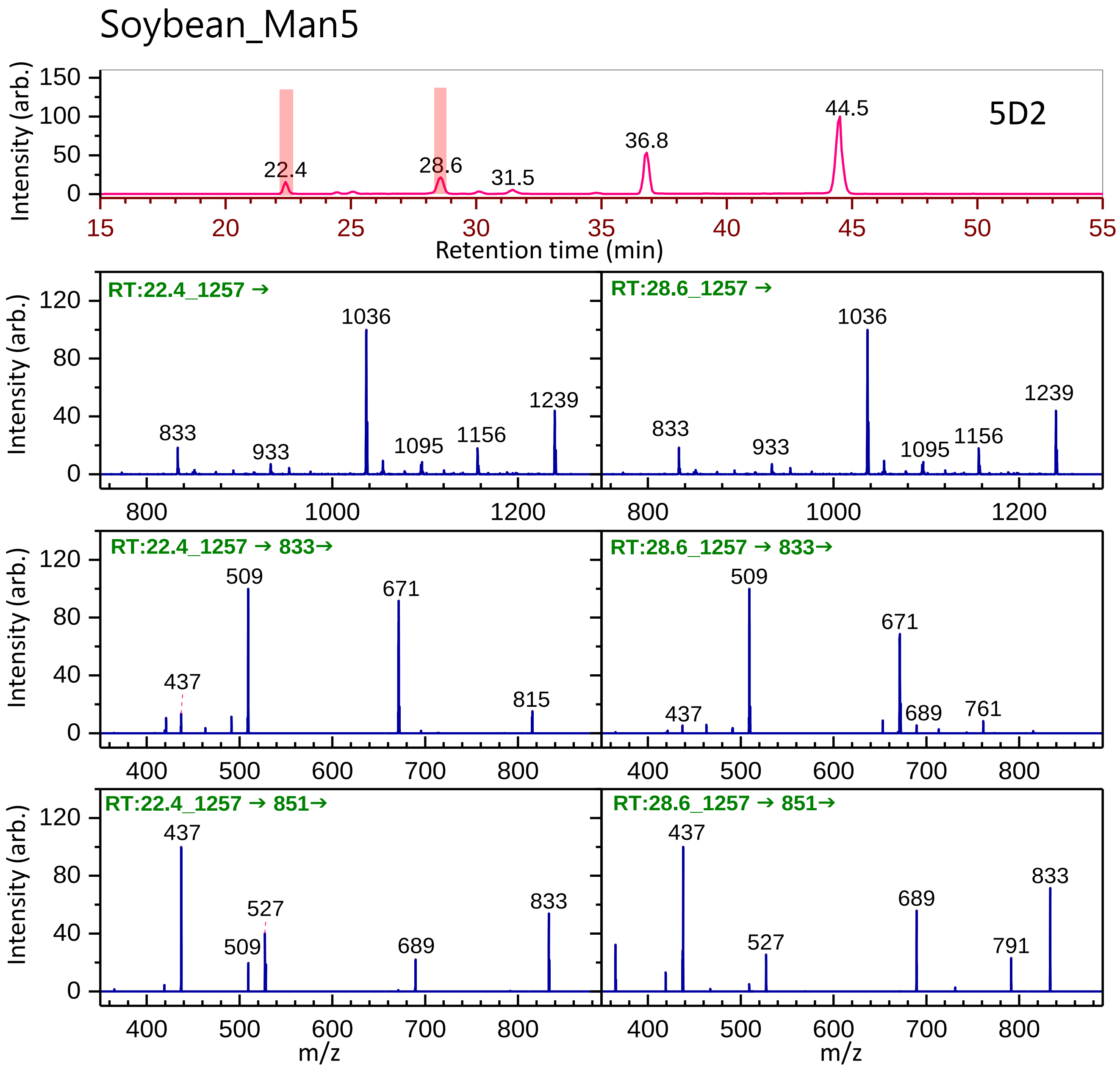

Figure S35. Chromatogram and MS^2 and MS^3 mass spectra of soybean $Man_5GlcNAC_2$.

Soybean_Man5

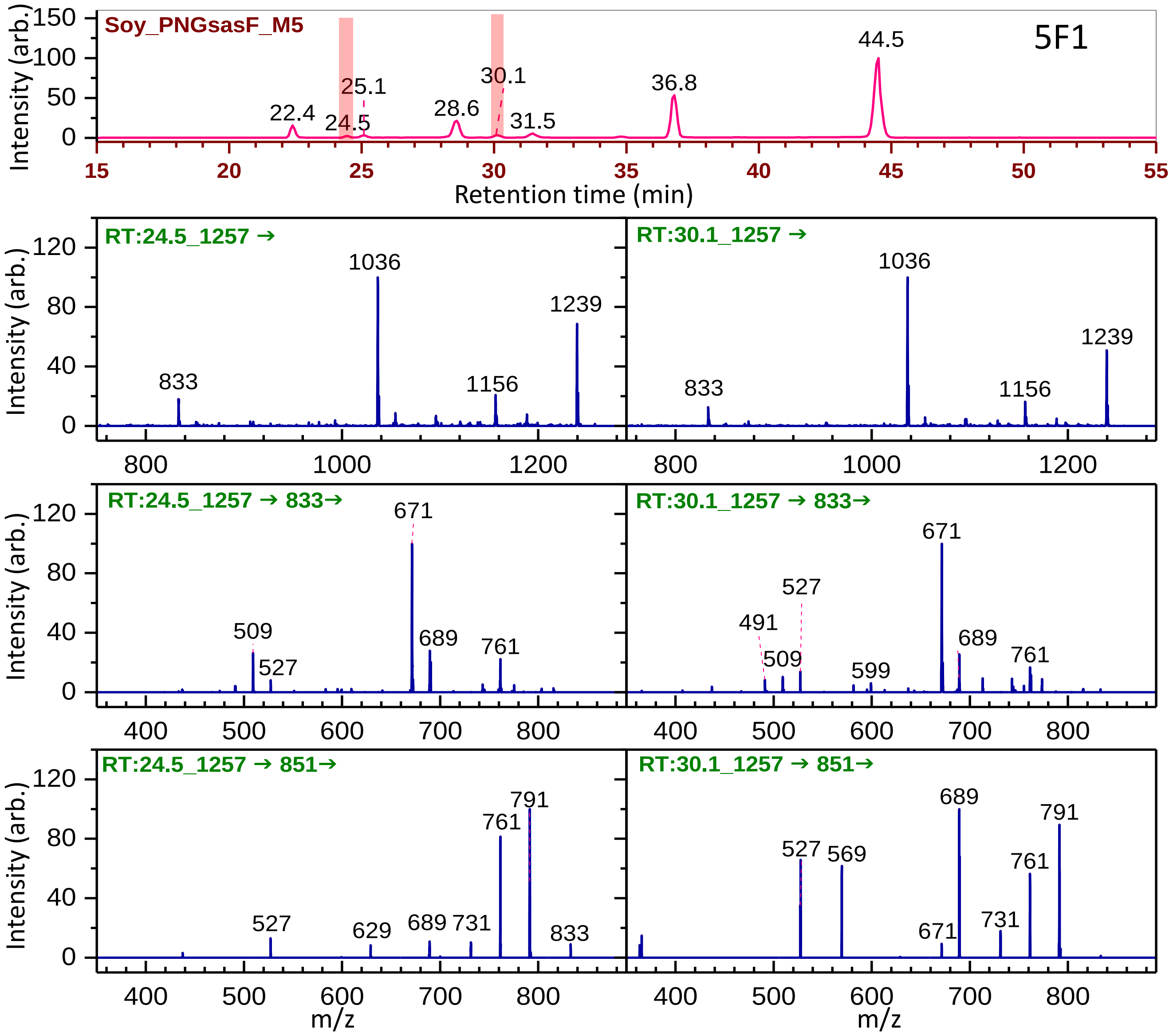

Figure S36. Chromatogram and MS^2 and MS^3 mass spectra of soybean $Man_5GlcNAC_2$.

Soybean_Man5

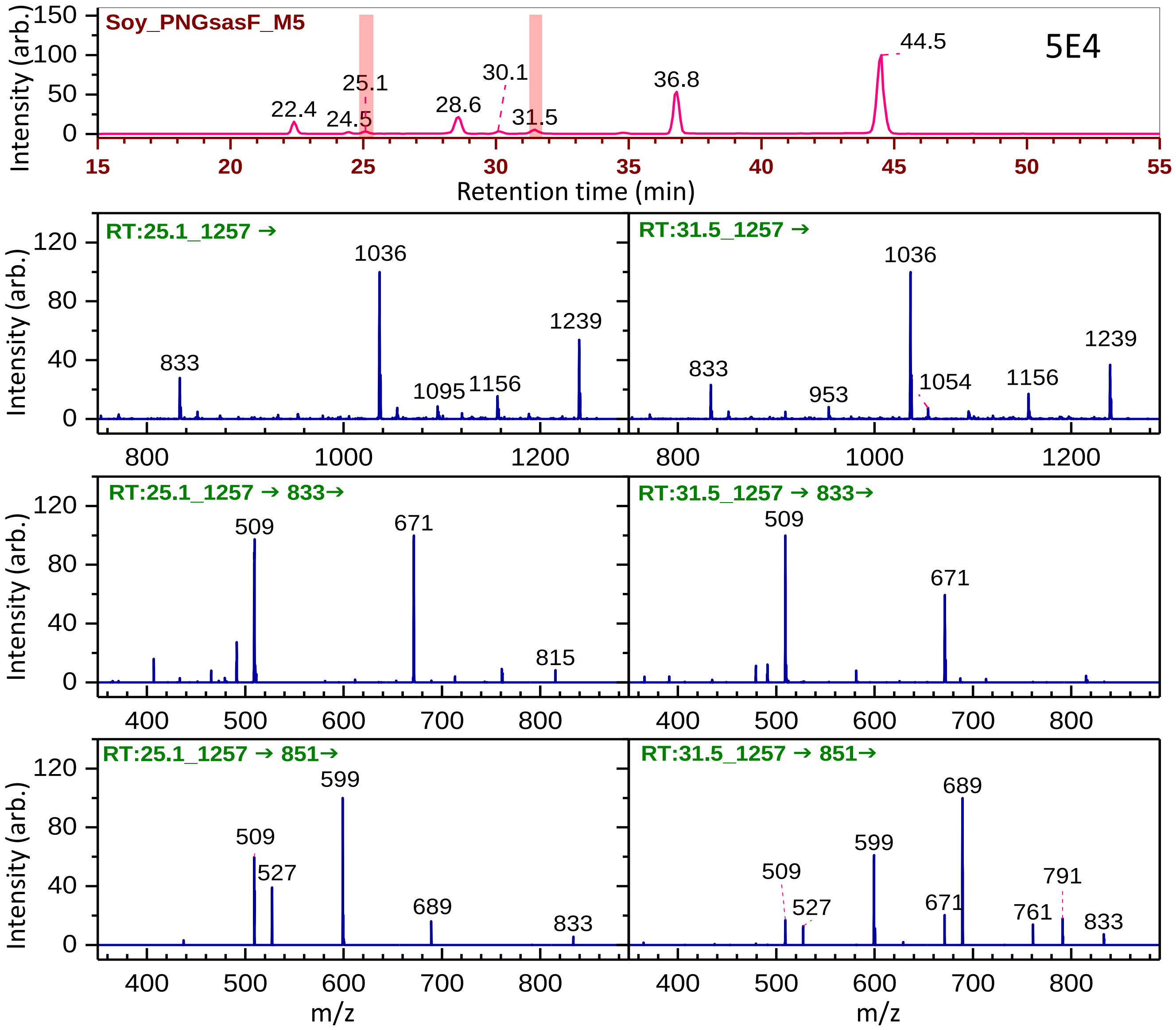

Figure S37. Chromatogram and MS^2 and MS^3 mass spectra of soybean $Man_5GlcNAC_2$.

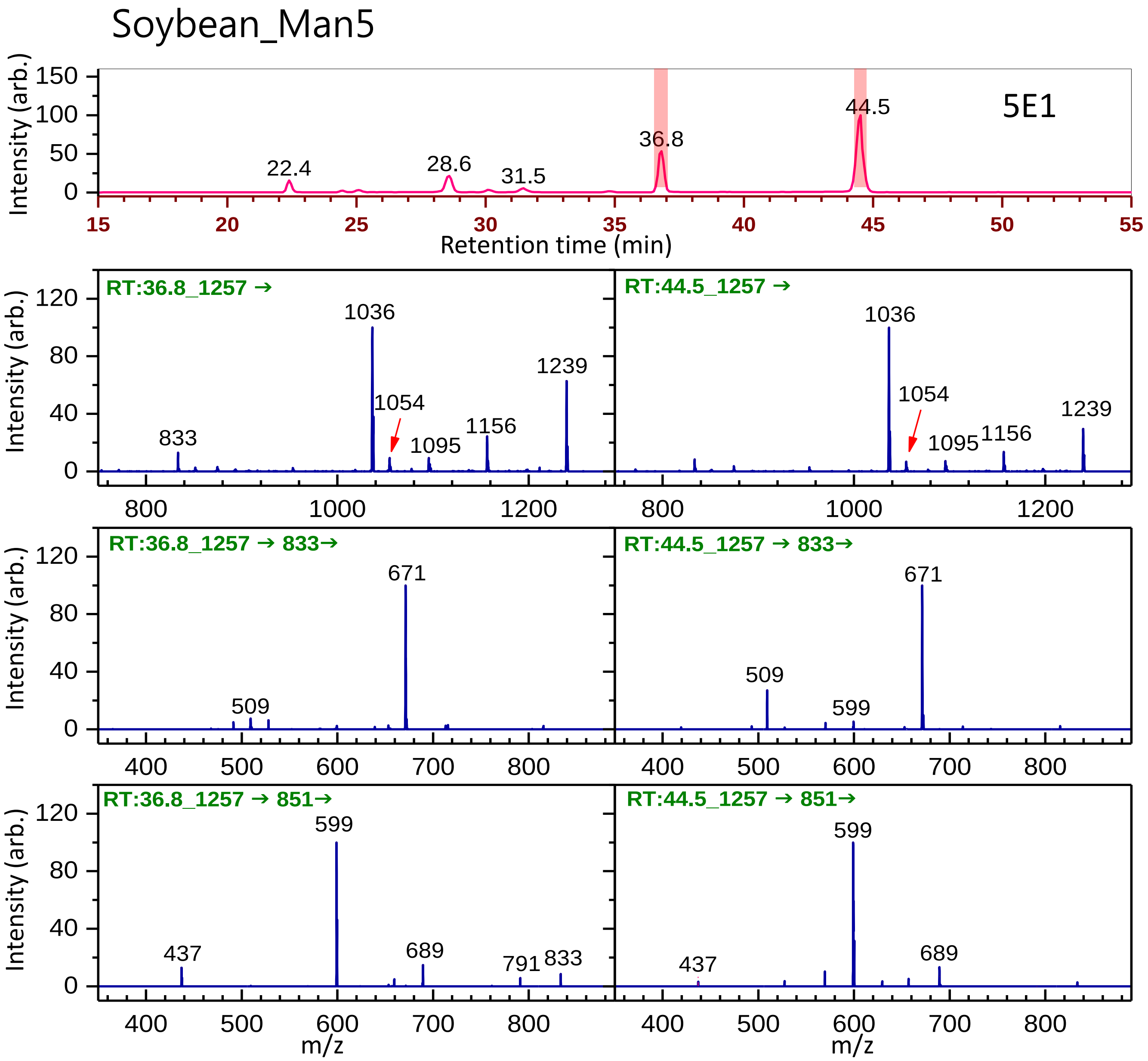

Figure S38. Chromatogram and MS^2 and MS^3 mass spectra of soybean $Man_5GlcNAC_2$.

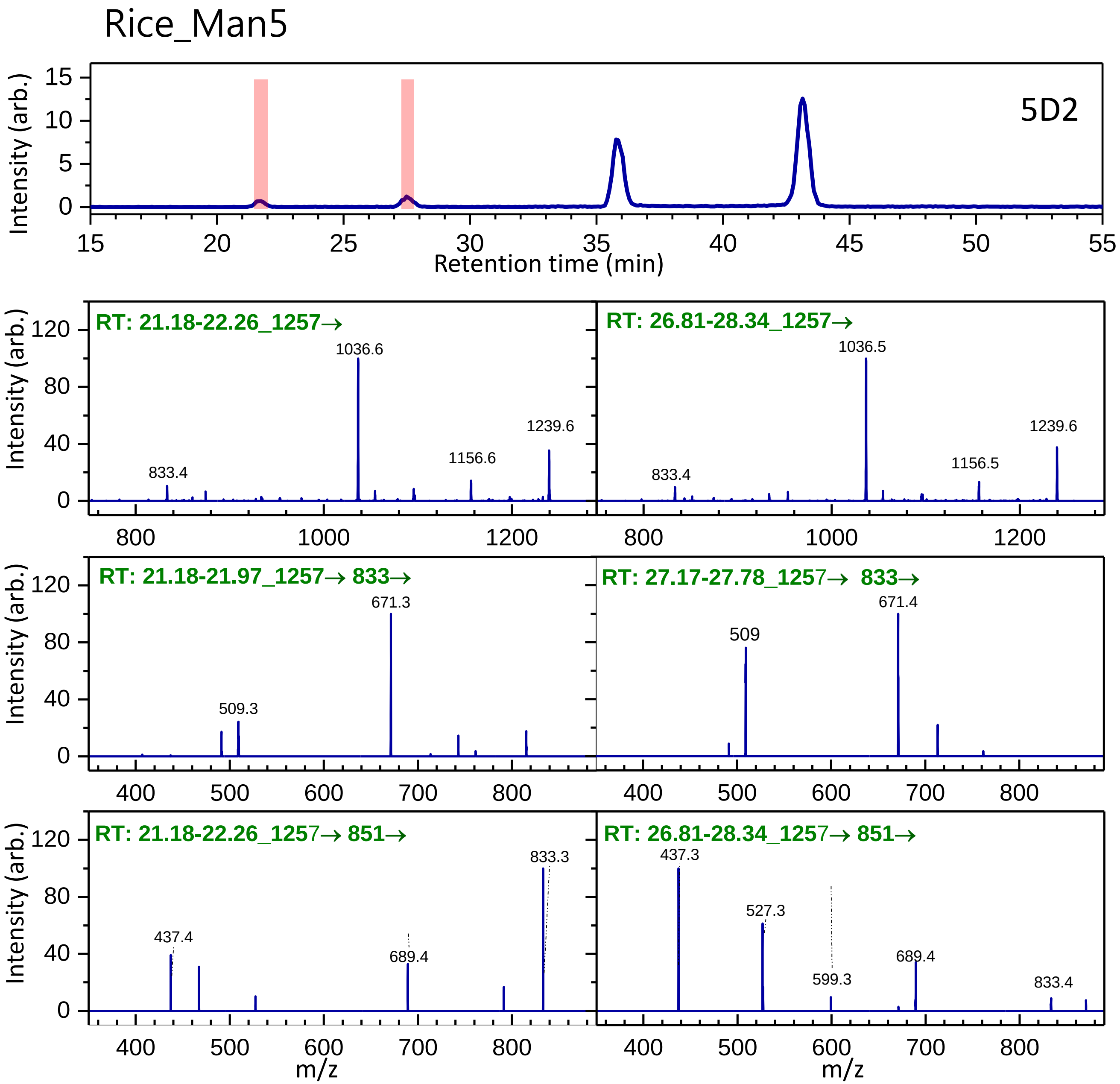

Figure S39. Chromatogram and MS^2 and MS^3 mass spectra of rice $Man_5GlcNAC_2$.

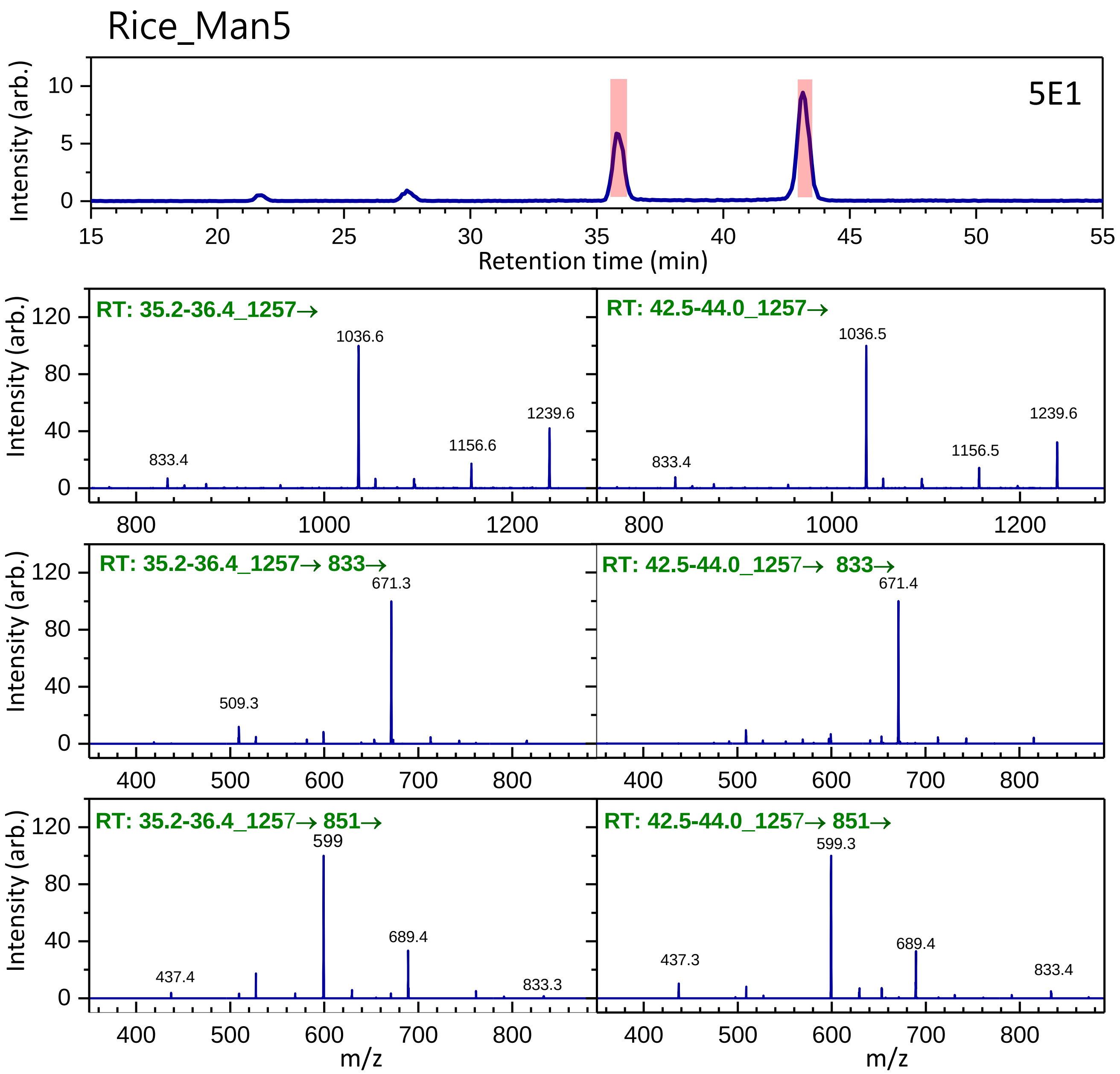

Figure S40. Chromatogram and MS^2 and MS^3 mass spectra of rice $Man_5GlcNAC_2$.

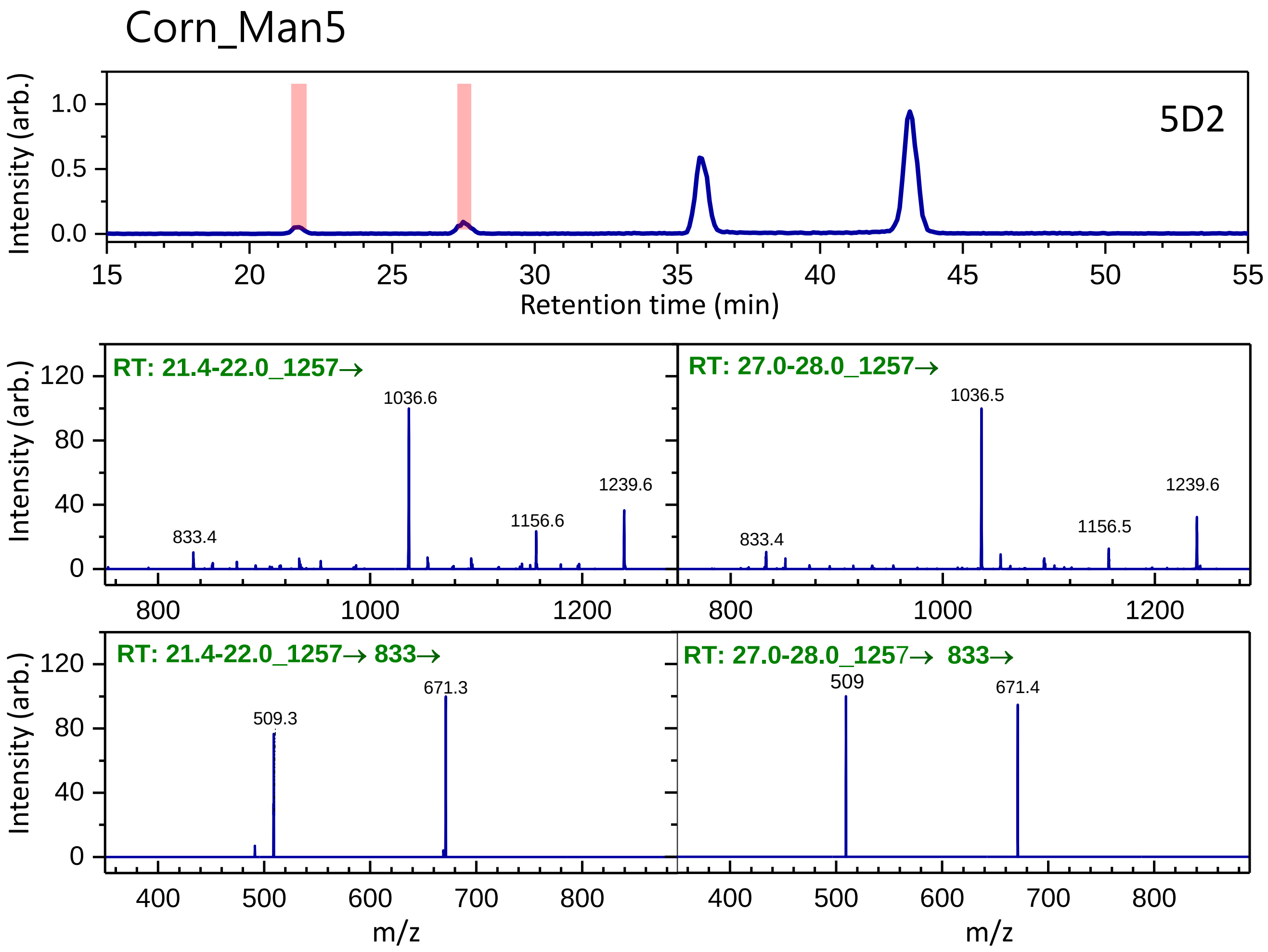

Figure S41. Chromatogram and MS^2 and MS^3 mass spectra of corn $Man_5GlcNAC_2$.

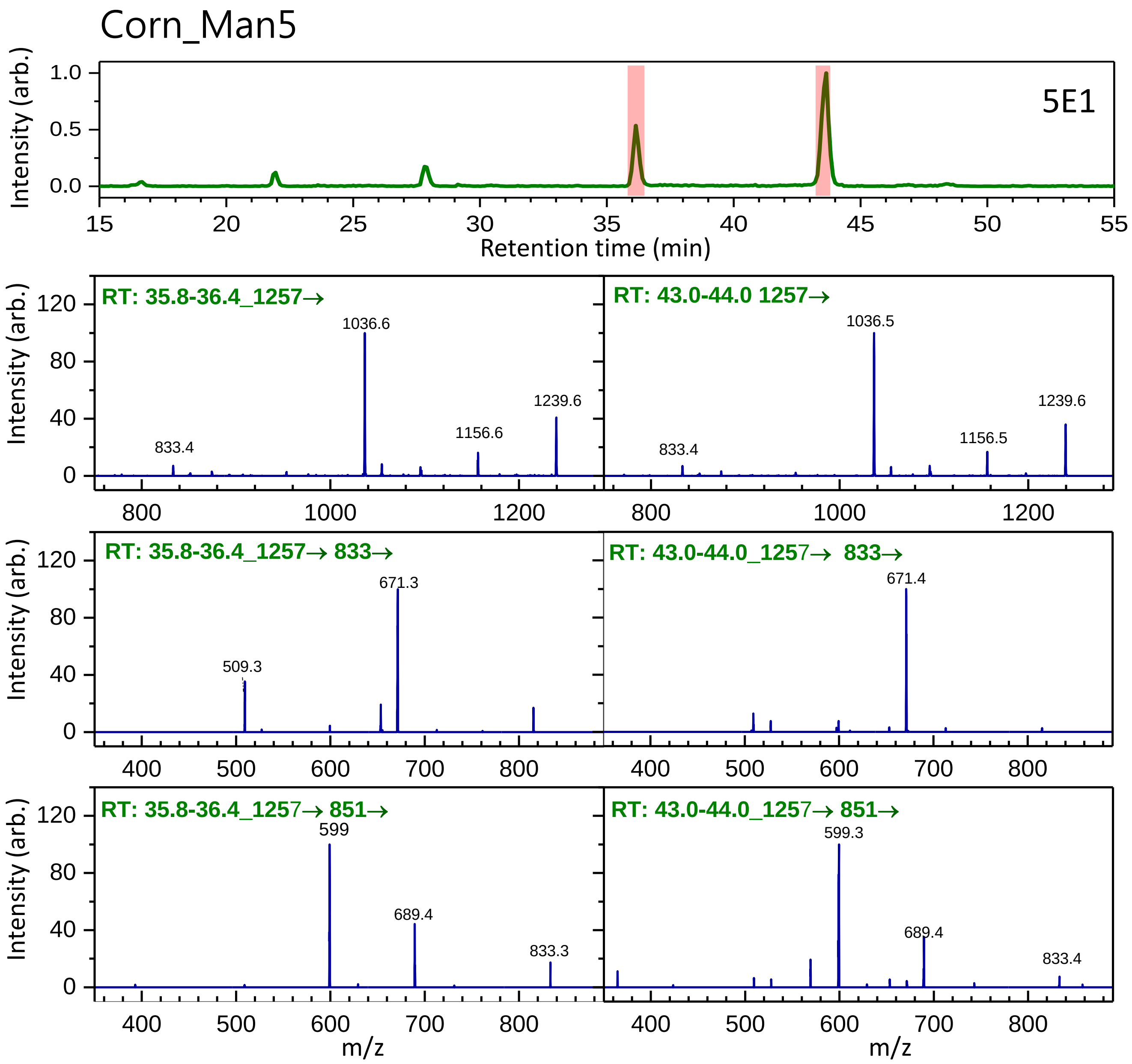

Figure S42. Chromatogram and MS^2 and MS^3 mass spectra of corn $Man_5GlcNAC_2$.

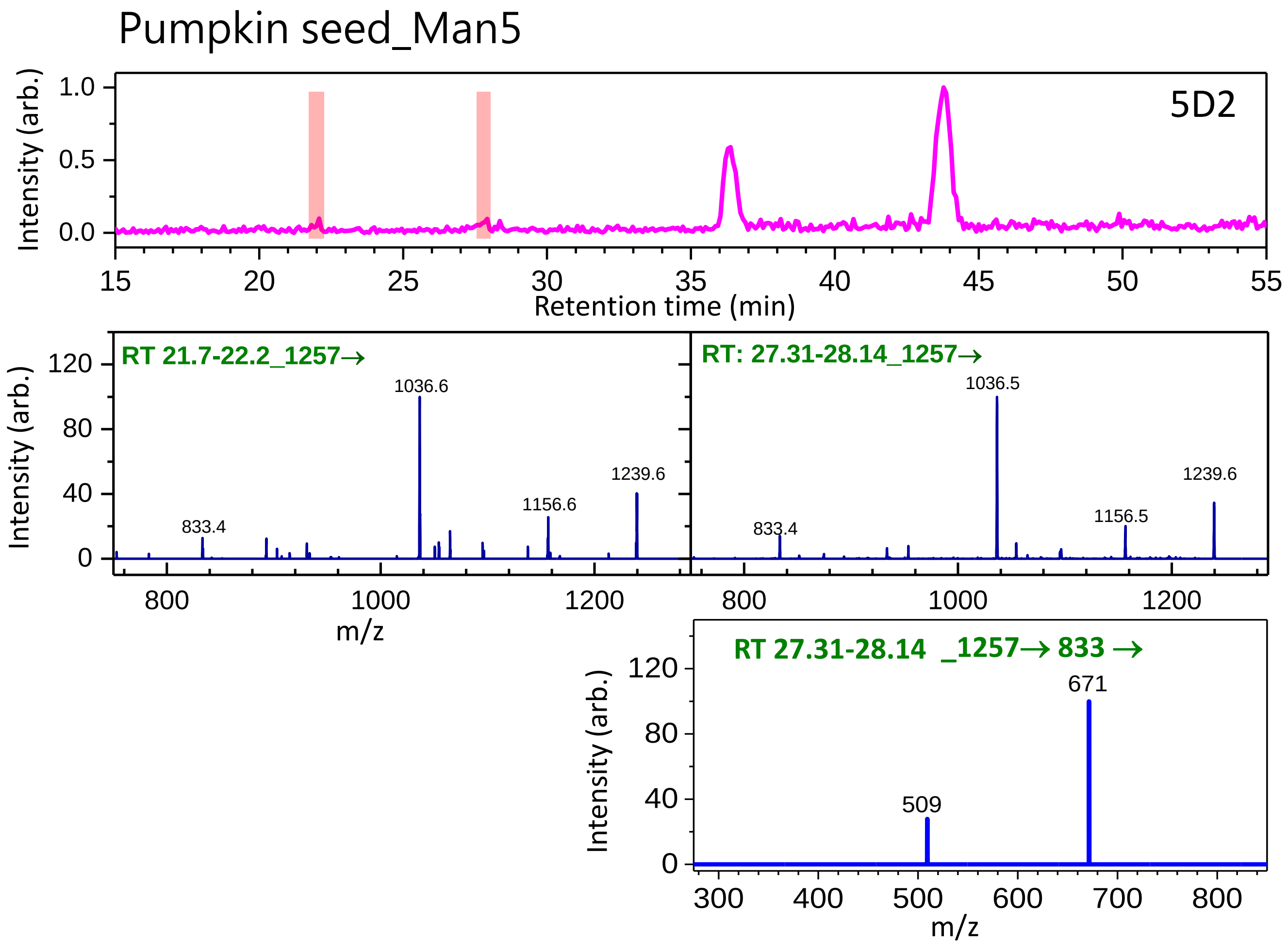

Figure S43. Chromatogram and MS^2 and MS^3 mass spectra of pumpkin seed $Man_5GlcNAC_2$.

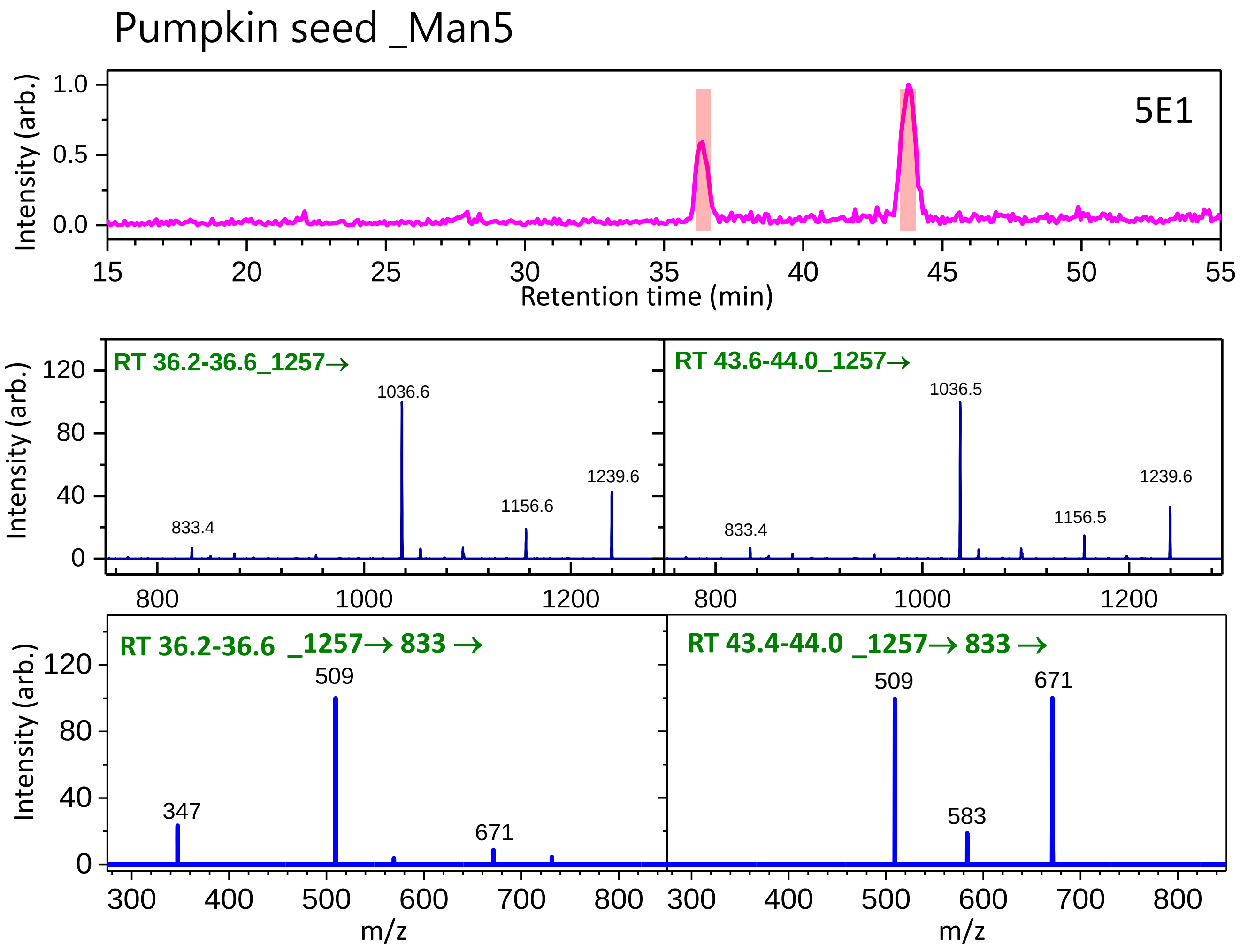

Figure S44. Chromatogram and MS^2 and MS^3 mass spectra of pumpkin seed $Man_5GlcNAC_2$.

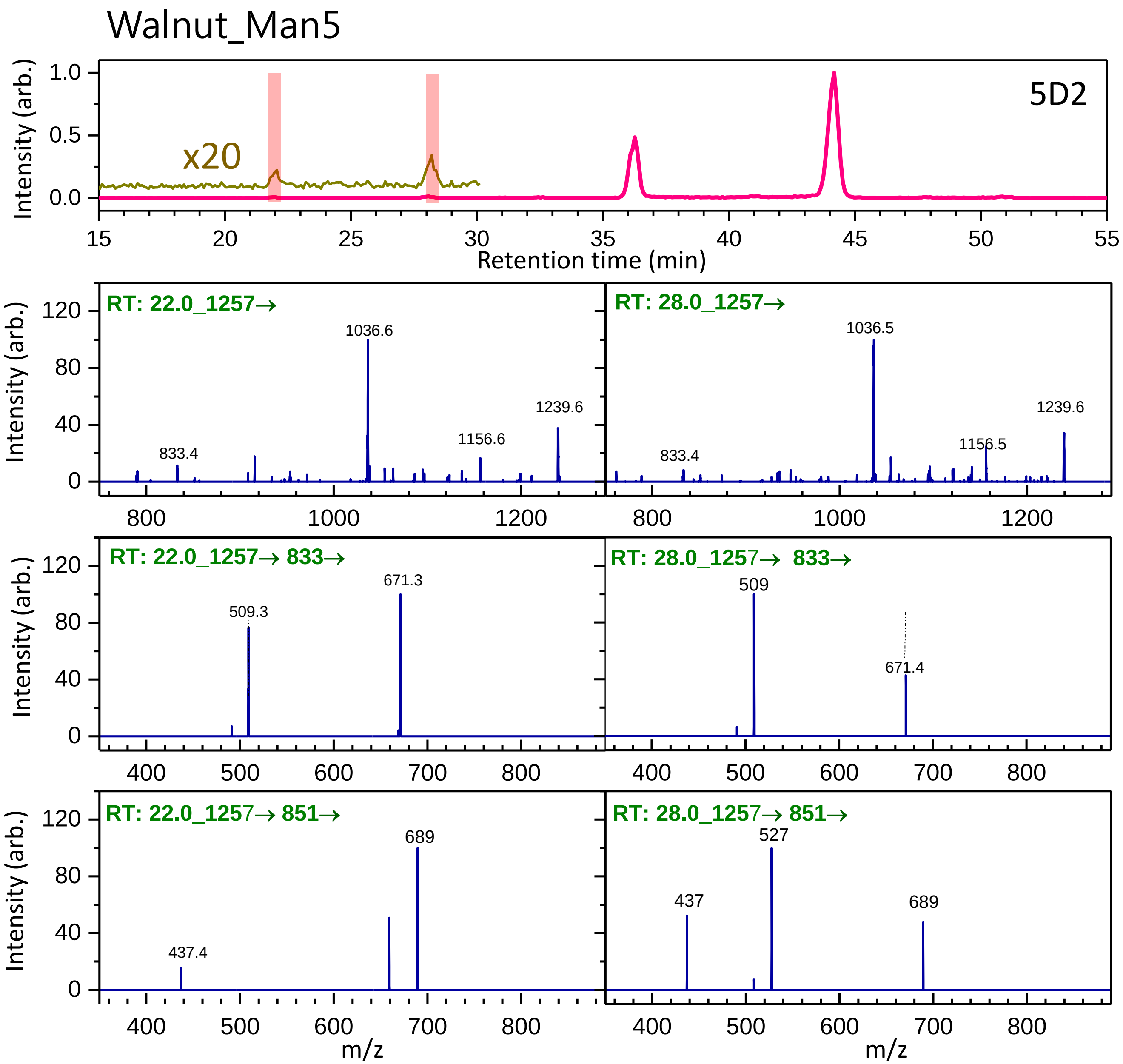

Figure S45. Chromatogram and MS^2 and MS^3 mass spectra of walnut $Man_5GlcNAC_2$.

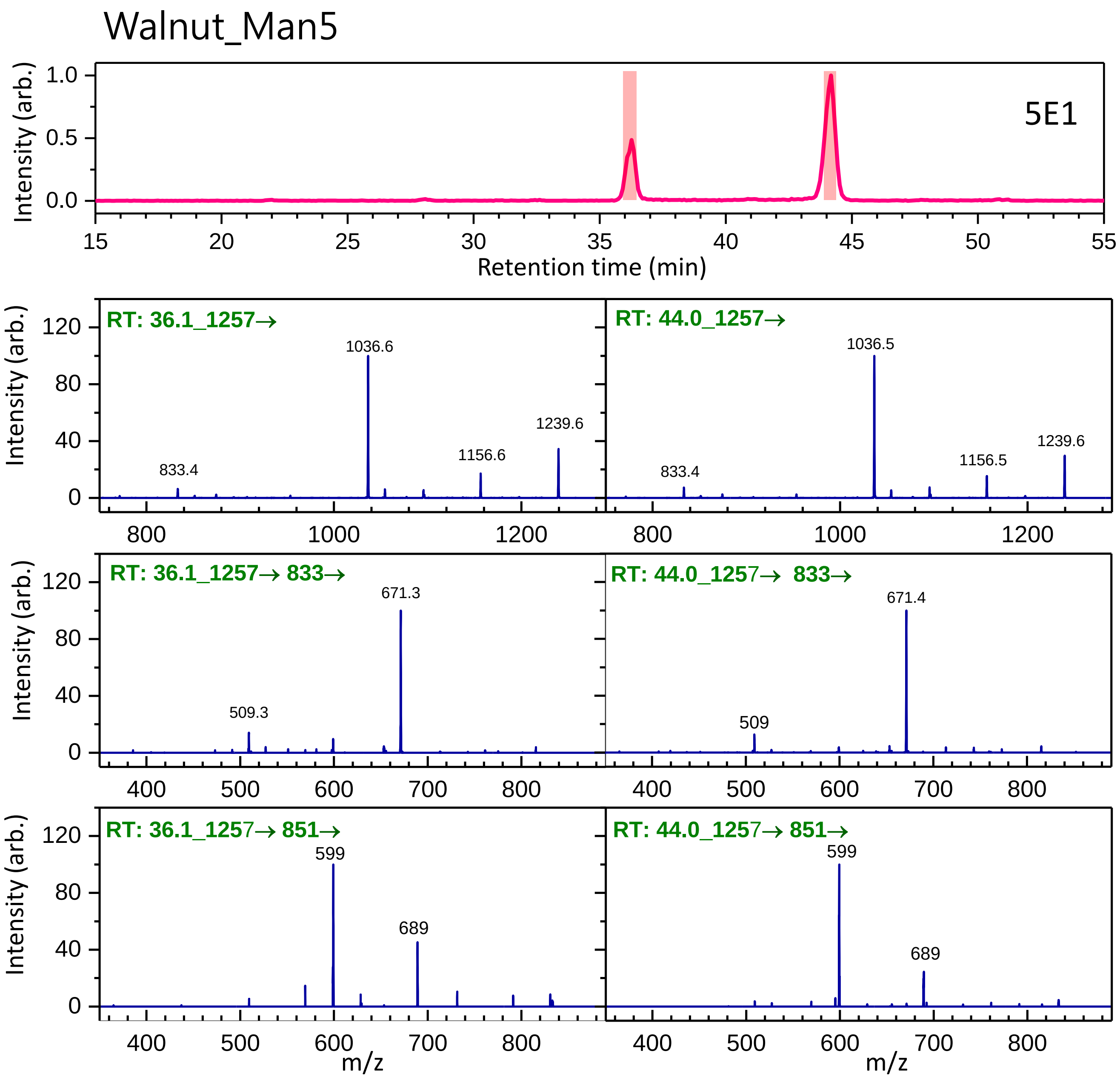

Figure S46. Chromatogram and MS^2 and MS^3 mass spectra of walnut $Man_5GlcNAC_2$.

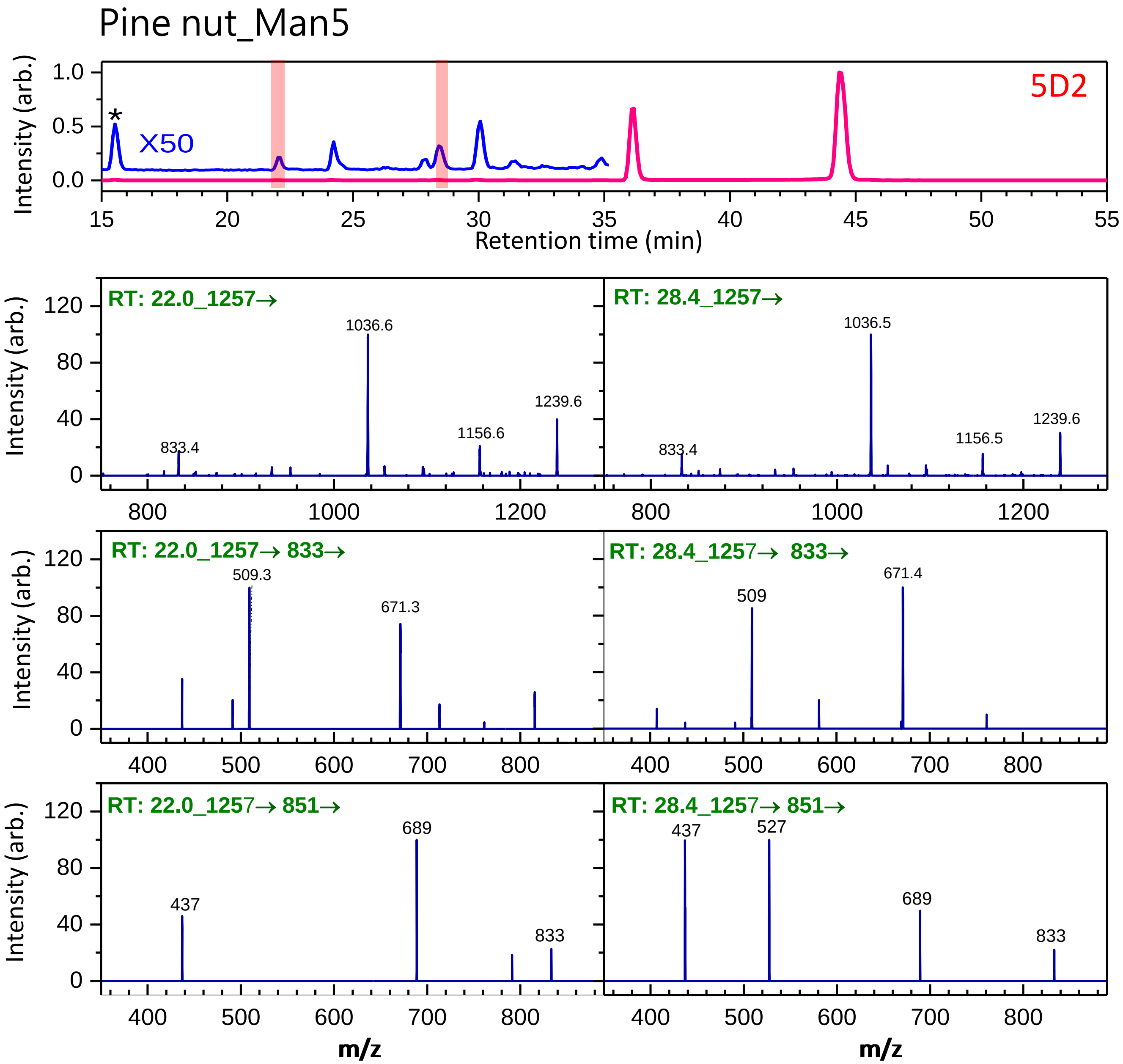

Figure S47. Chromatogram and MS^2 and MS^3 mass spectra of pine nut $Man_5GlcNAC_2$.

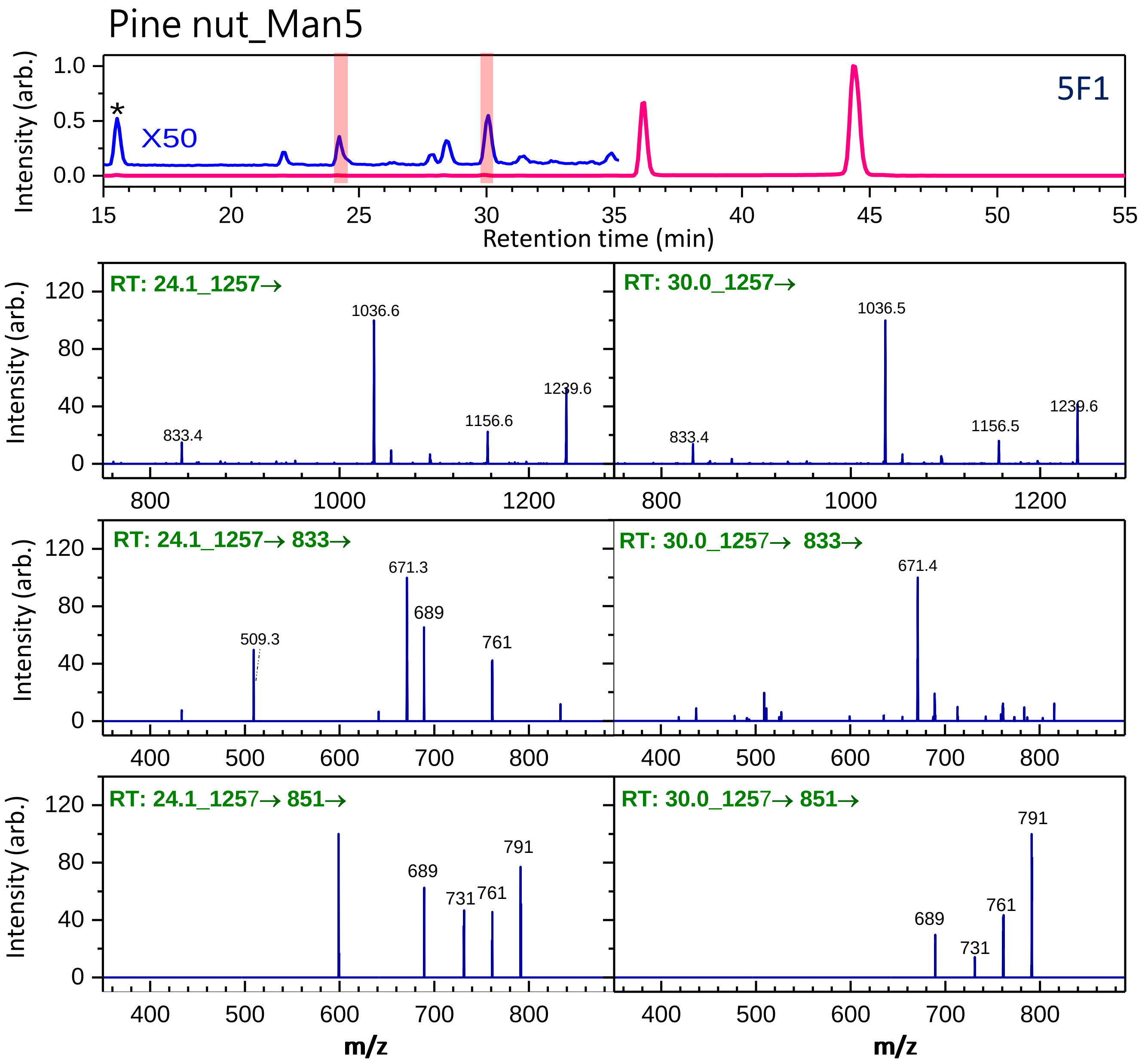

Figure S48. Chromatogram and MS^2 and MS^3 mass spectra of pine nut $Man_5GlcNAC_2$.

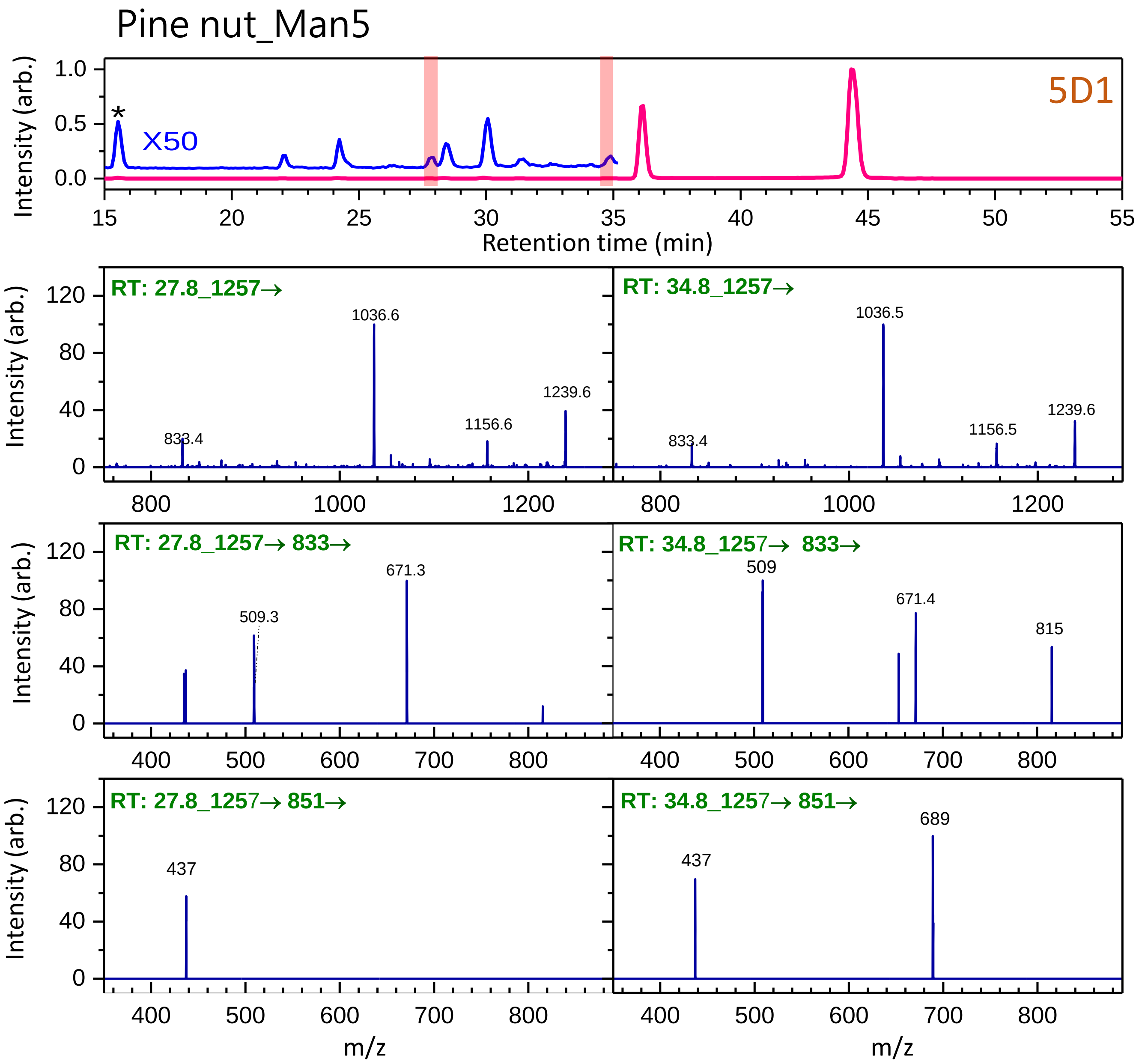

Figure S49. Chromatogram and MS^2 and MS^3 mass spectra of pine nut $Man_5GlcNAC_2$.

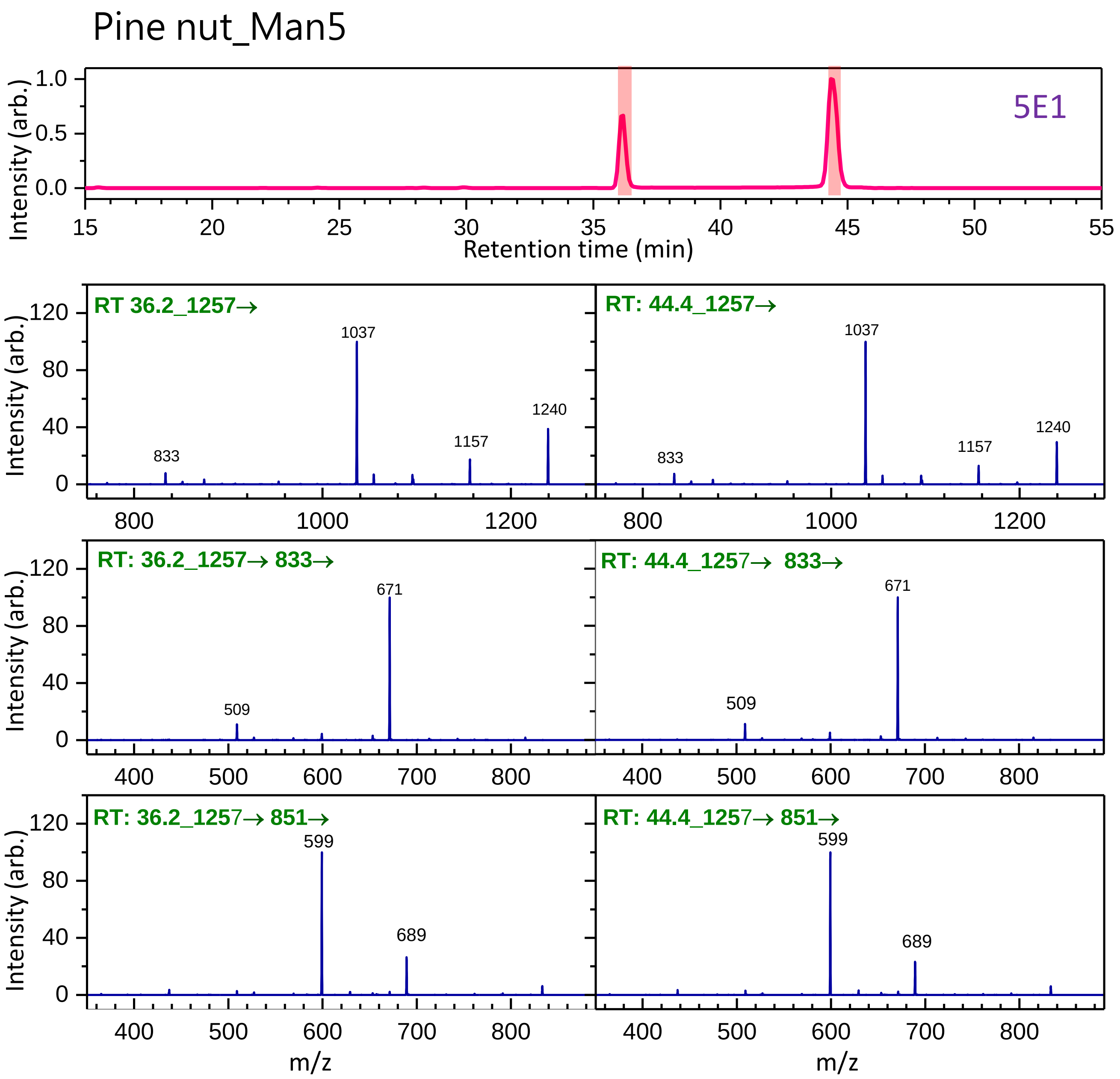

Figure S50. Chromatogram and MS^2 and MS^3 mass spectra of pine nut $Man_5GlcNAC_2$.

$Man_6GlcNAC_2$

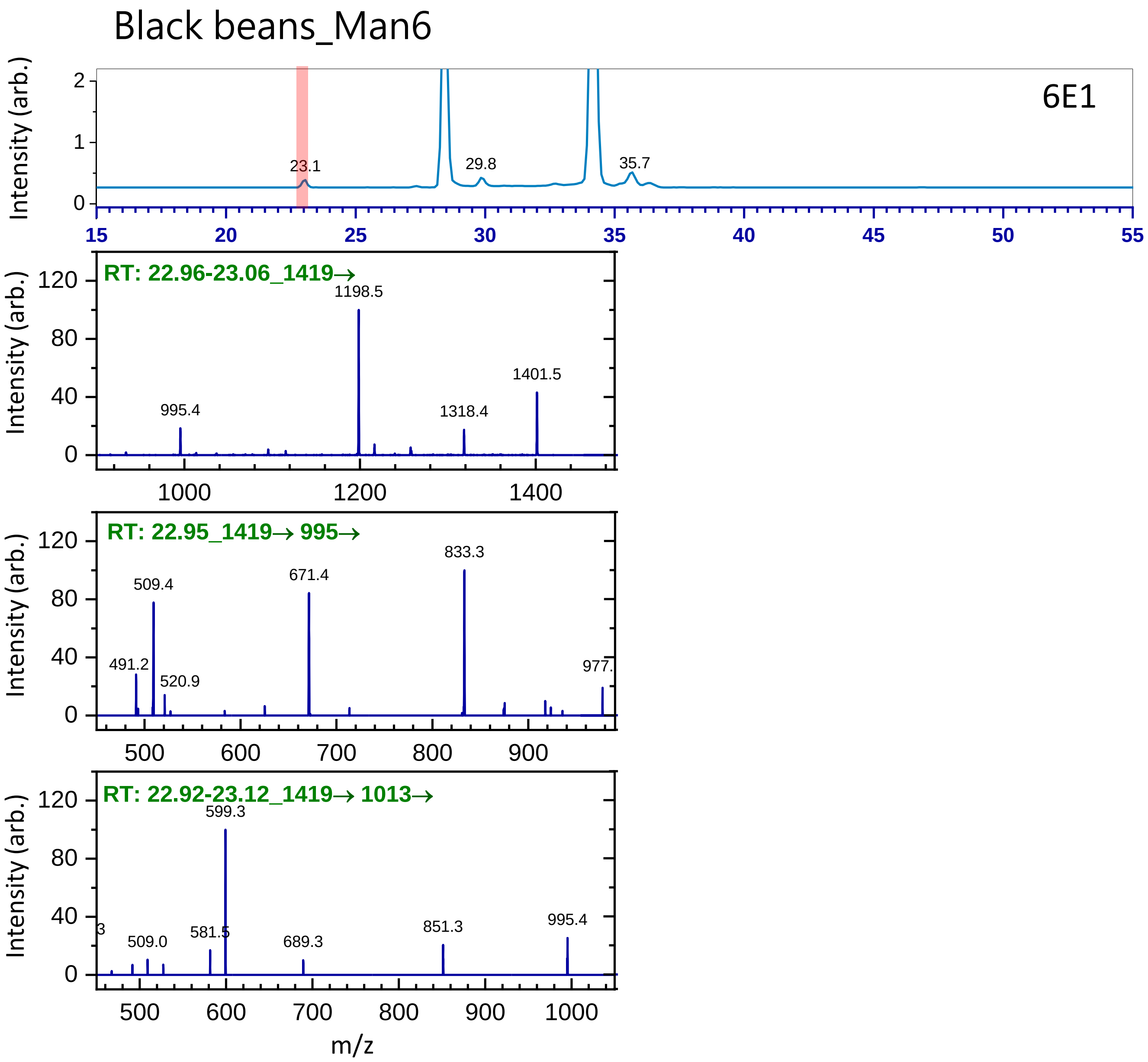

Figure S51. Chromatogram and MS^2 and MS^3 mass spectra of black bean $Man_6GlcNAC_2$.

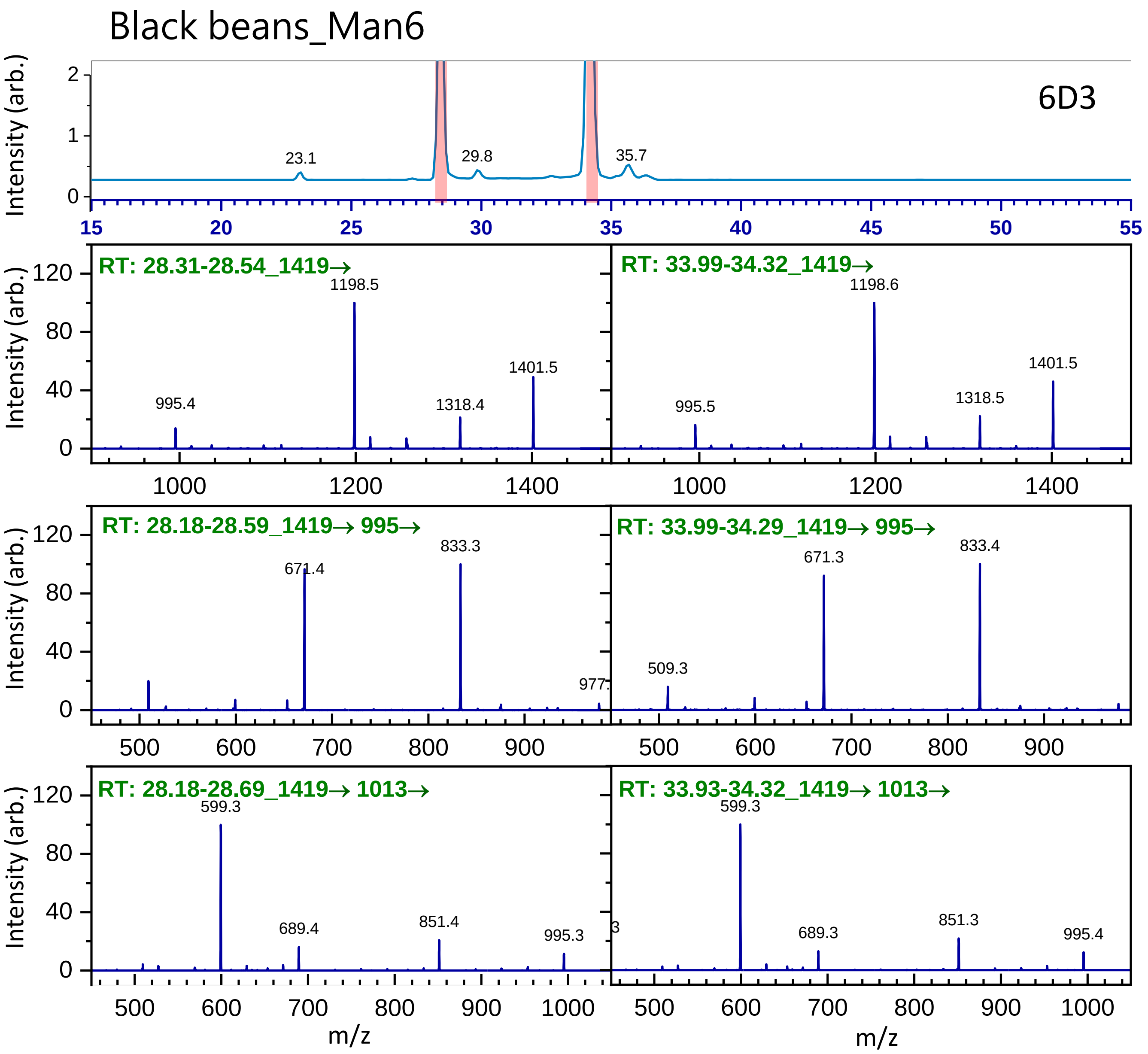

Figure S52. Chromatogram and MS^2 and MS^3 mass spectra of black bean $Man_6GlcNAC_2$.

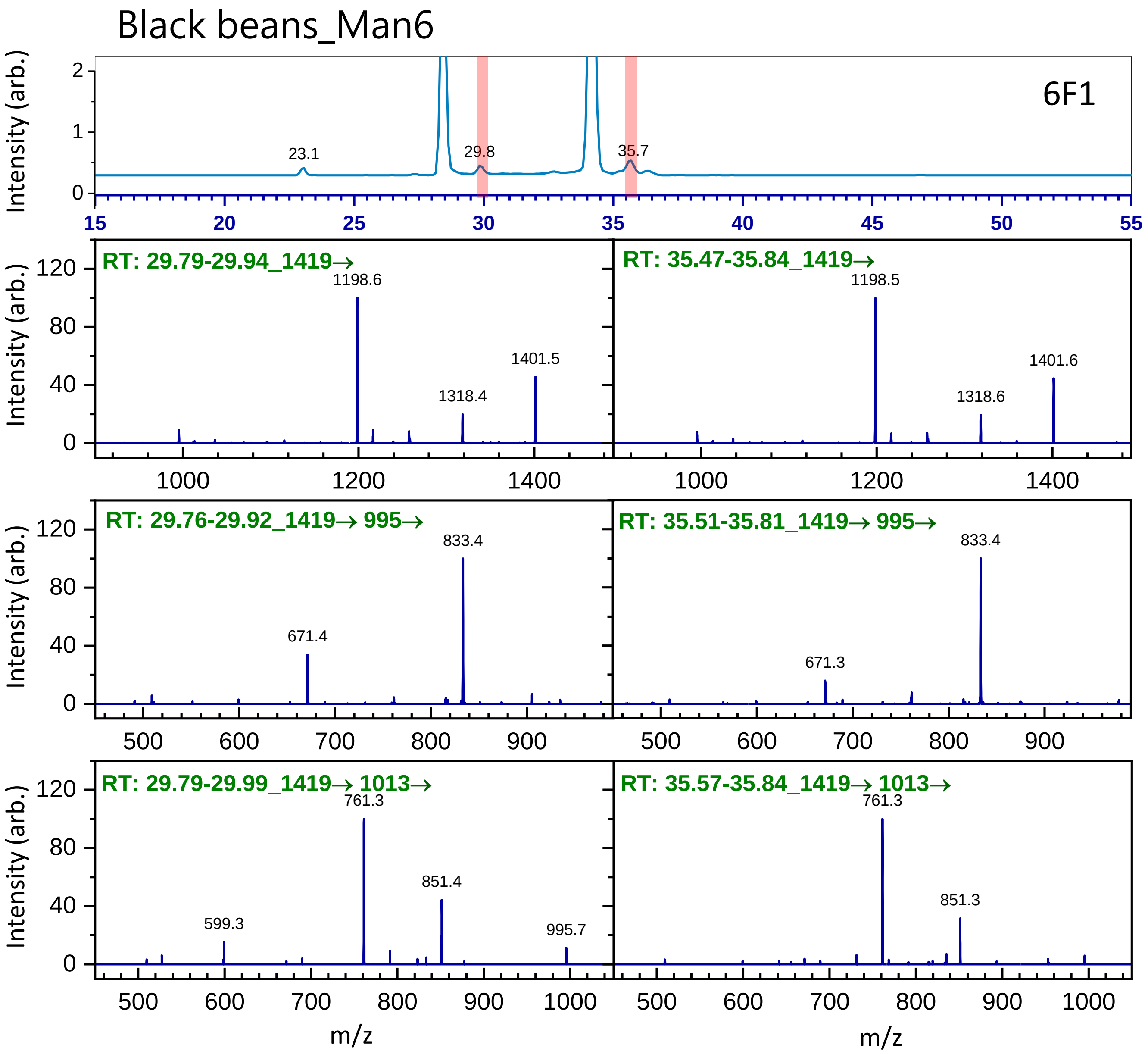

Figure S53. Chromatogram and MS^2 and MS^3 mass spectra of black bean $Man_6GlcNAC_2$.

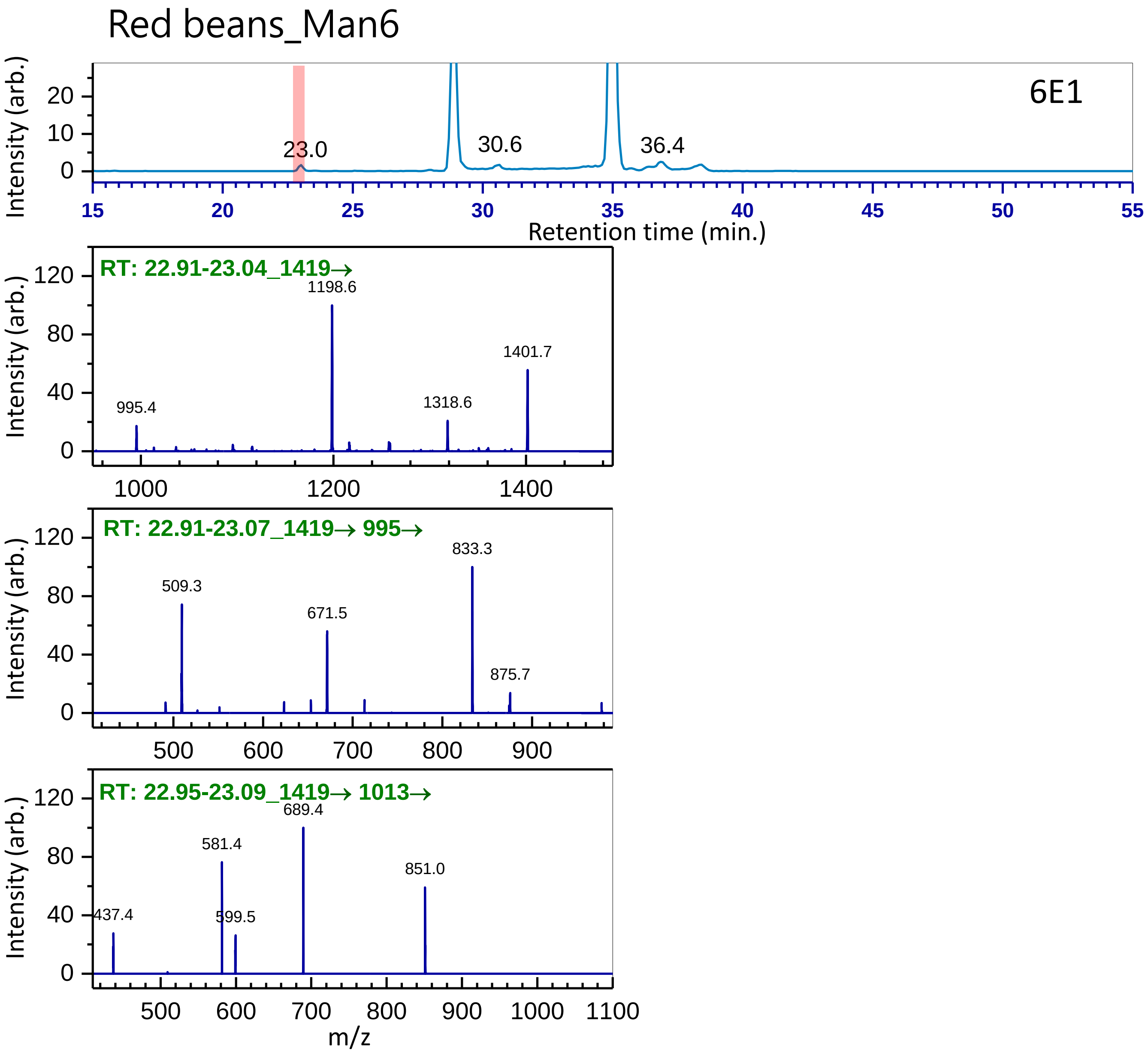

Figure S54. Chromatogram and MS^2 and MS^3 mass spectra of red bean $Man_6GlcNAC_2$.

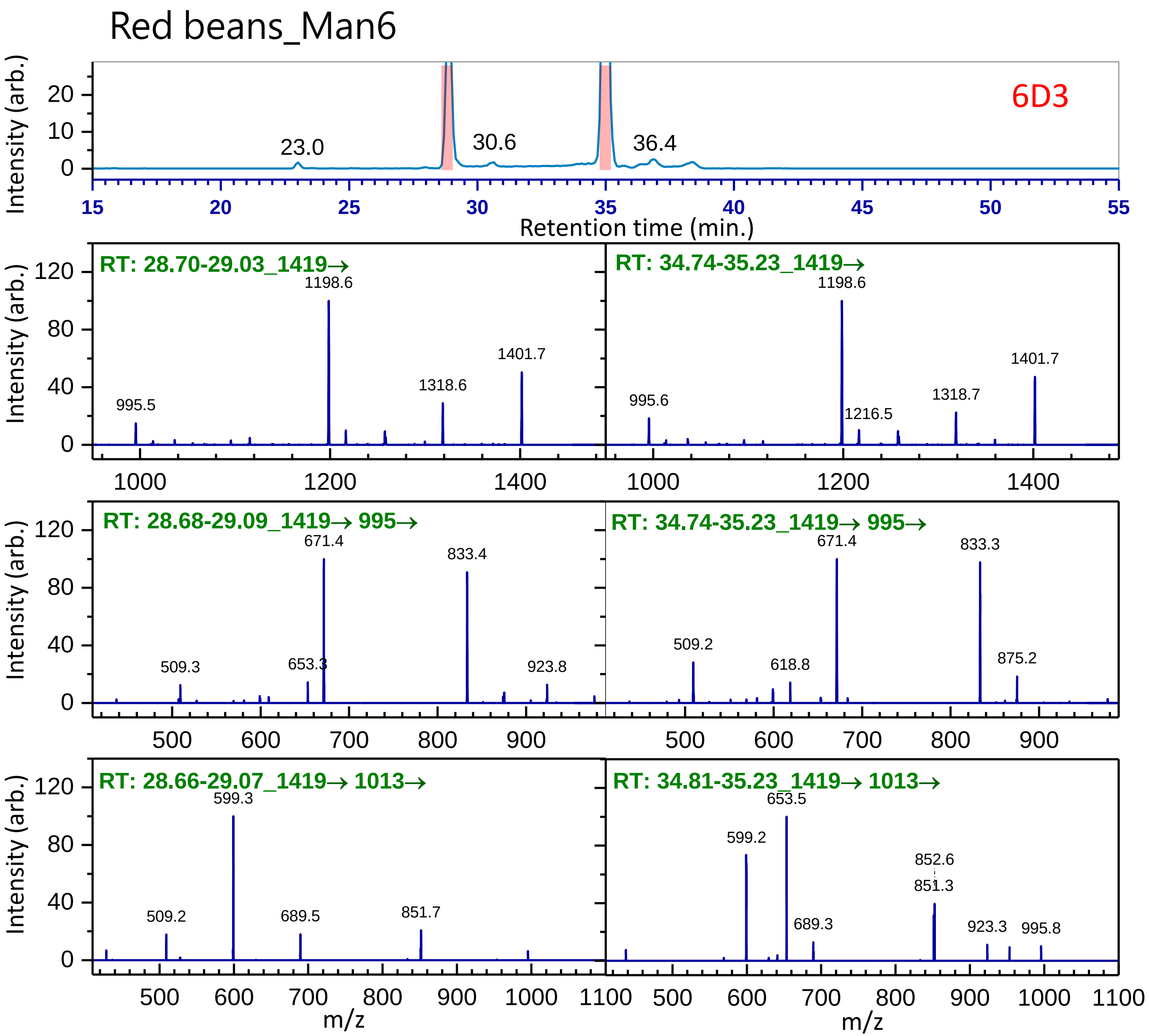

Figure S55. Chromatogram and MS^2 and MS^3 mass spectra of red bean $Man_6GlcNAC_2$.

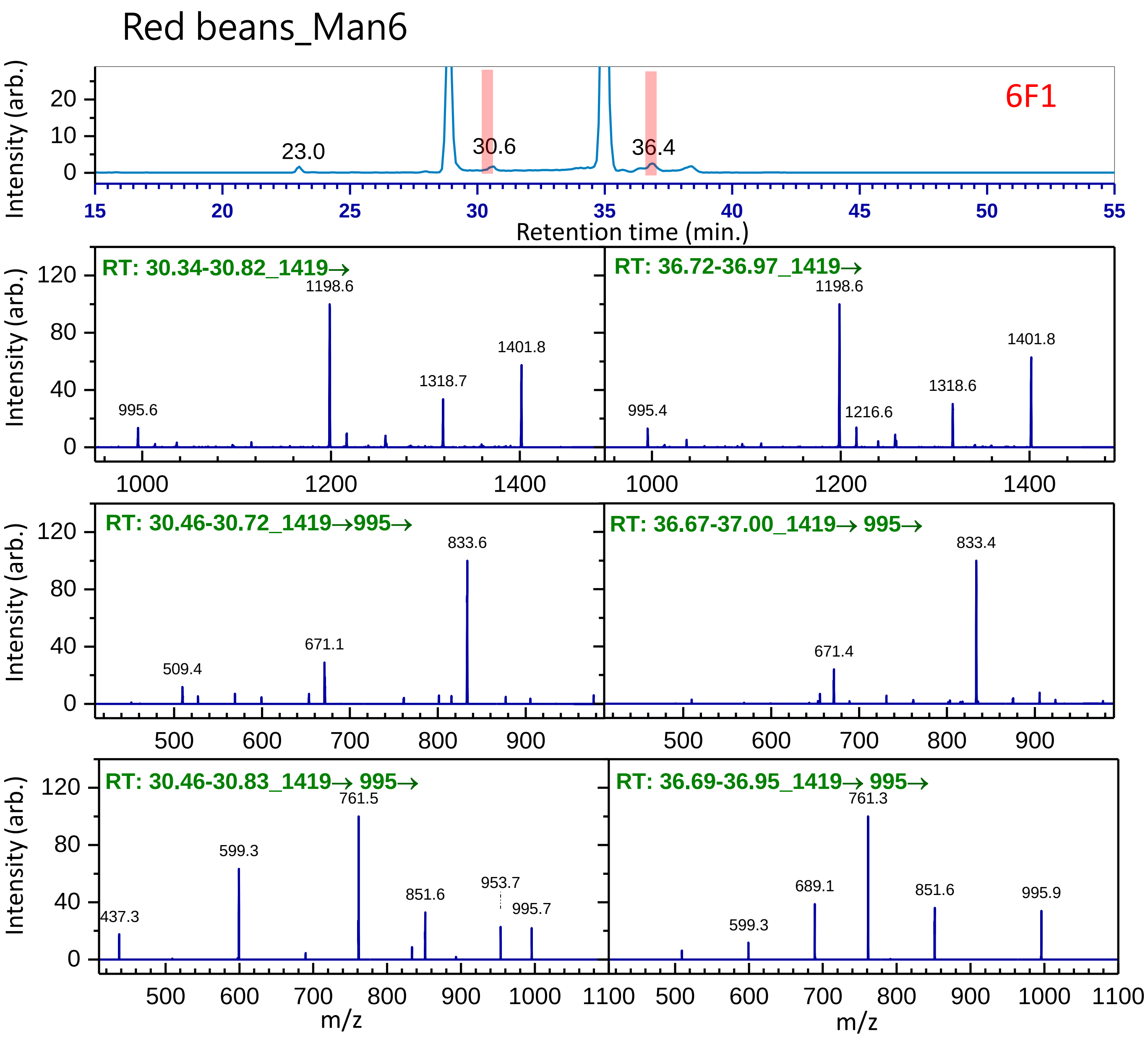

Figure S56. Chromatogram and MS^2 and MS^3 mass spectra of red bean $Man_6GlcNAC_2$.

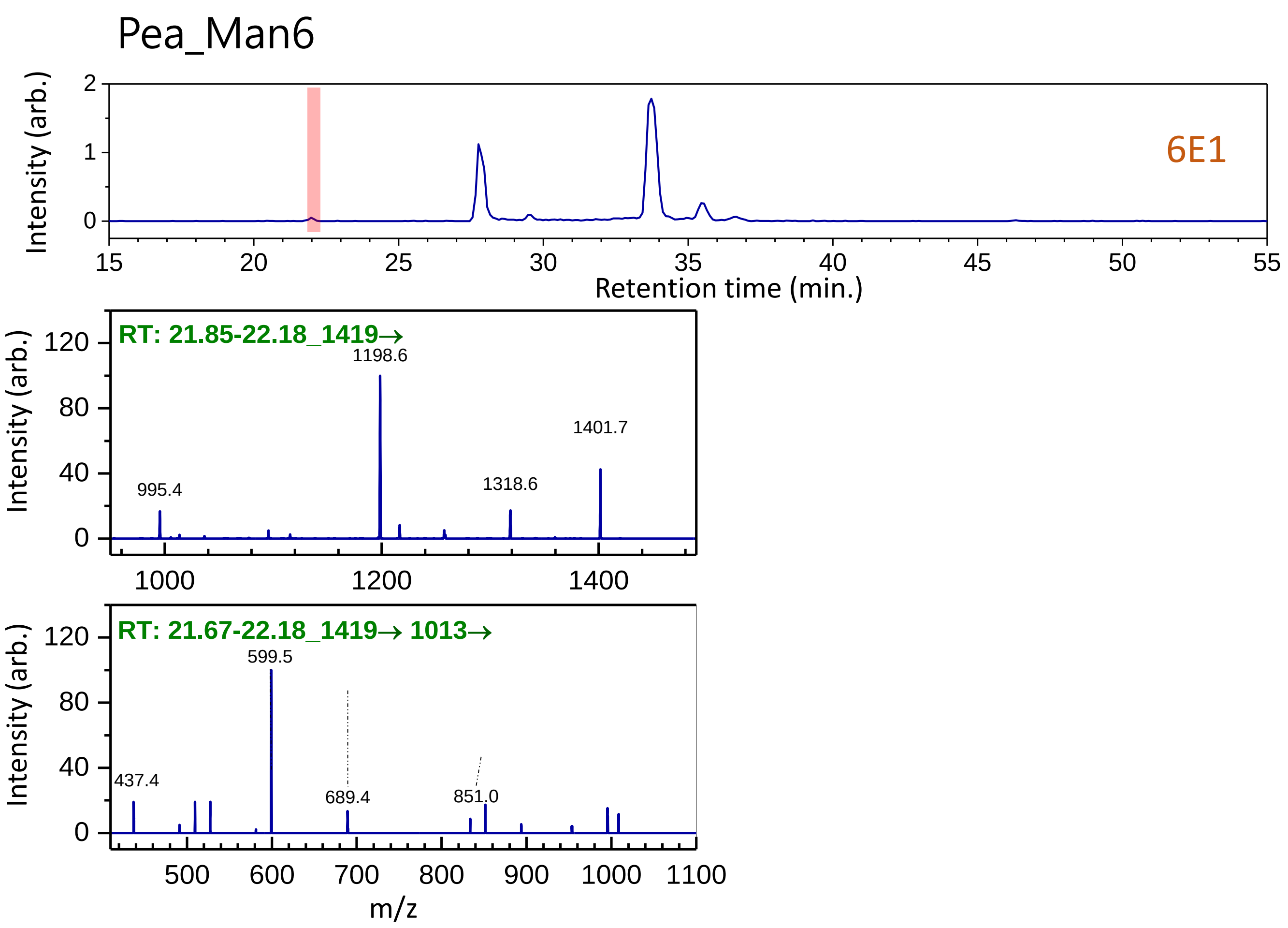

Figure S57. Chromatogram and MS^2 and MS^3 mass spectra of pea $Man_6GlcNAC_2$.

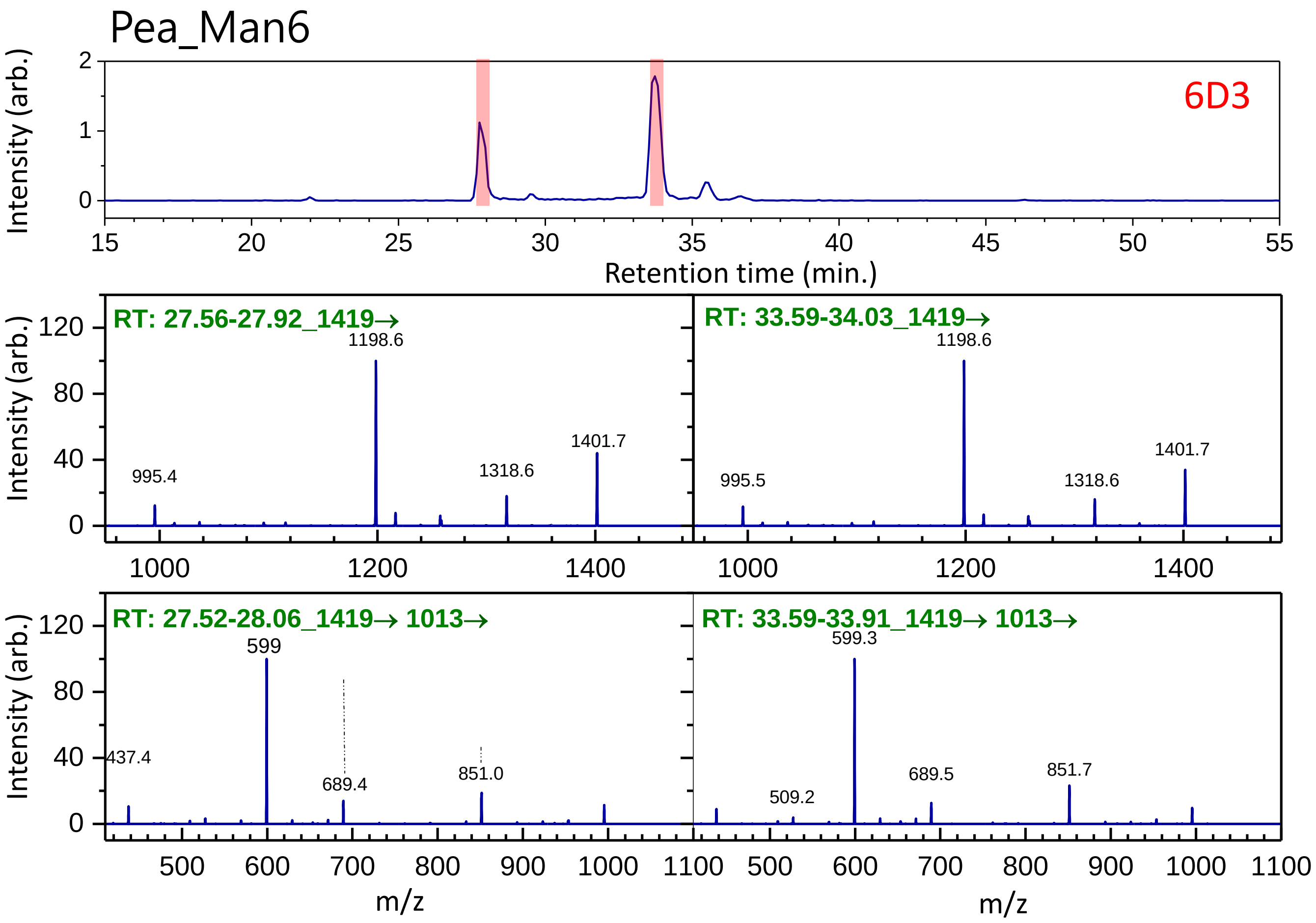

Figure S58. Chromatogram and MS^2 and MS^3 mass spectra of pea $Man_6GlcNAC_2$.

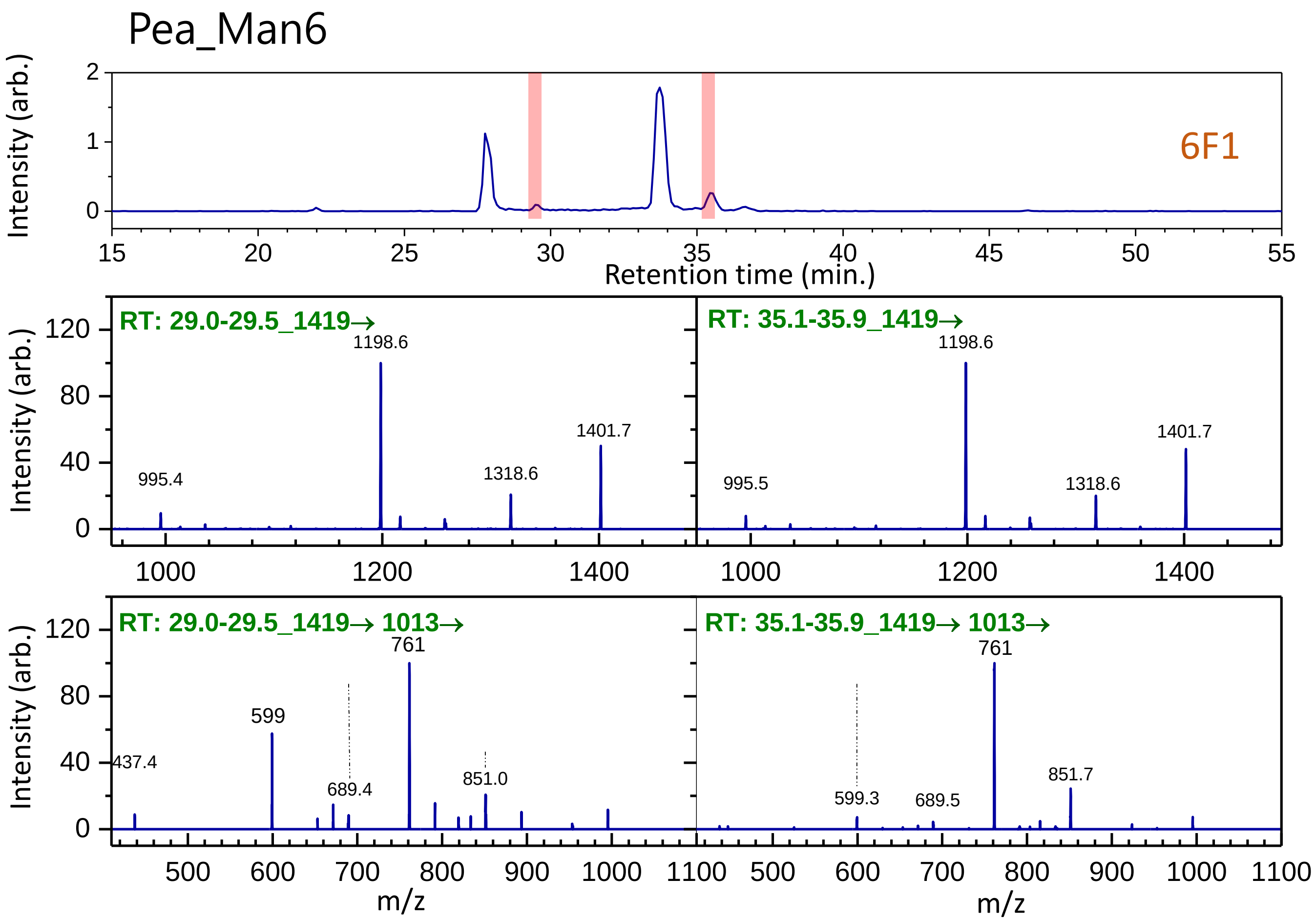

Figure S59. Chromatogram and MS^2 and MS^3 mass spectra of pea $Man_6GlcNAC_2$.

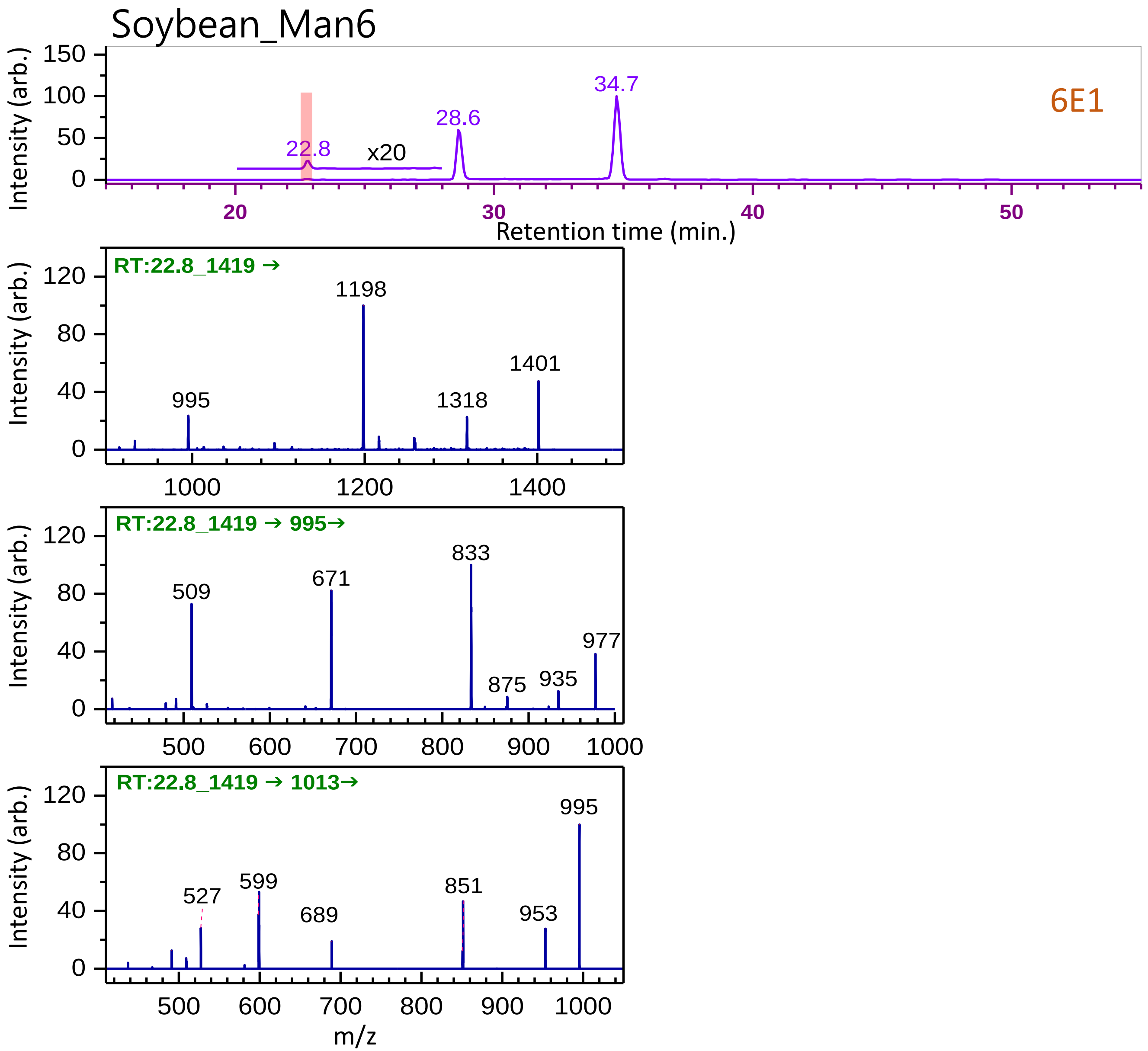

Figure S60. Chromatogram and MS^2 and MS^3 mass spectra of soybean $Man_6GlcNAC_2$.

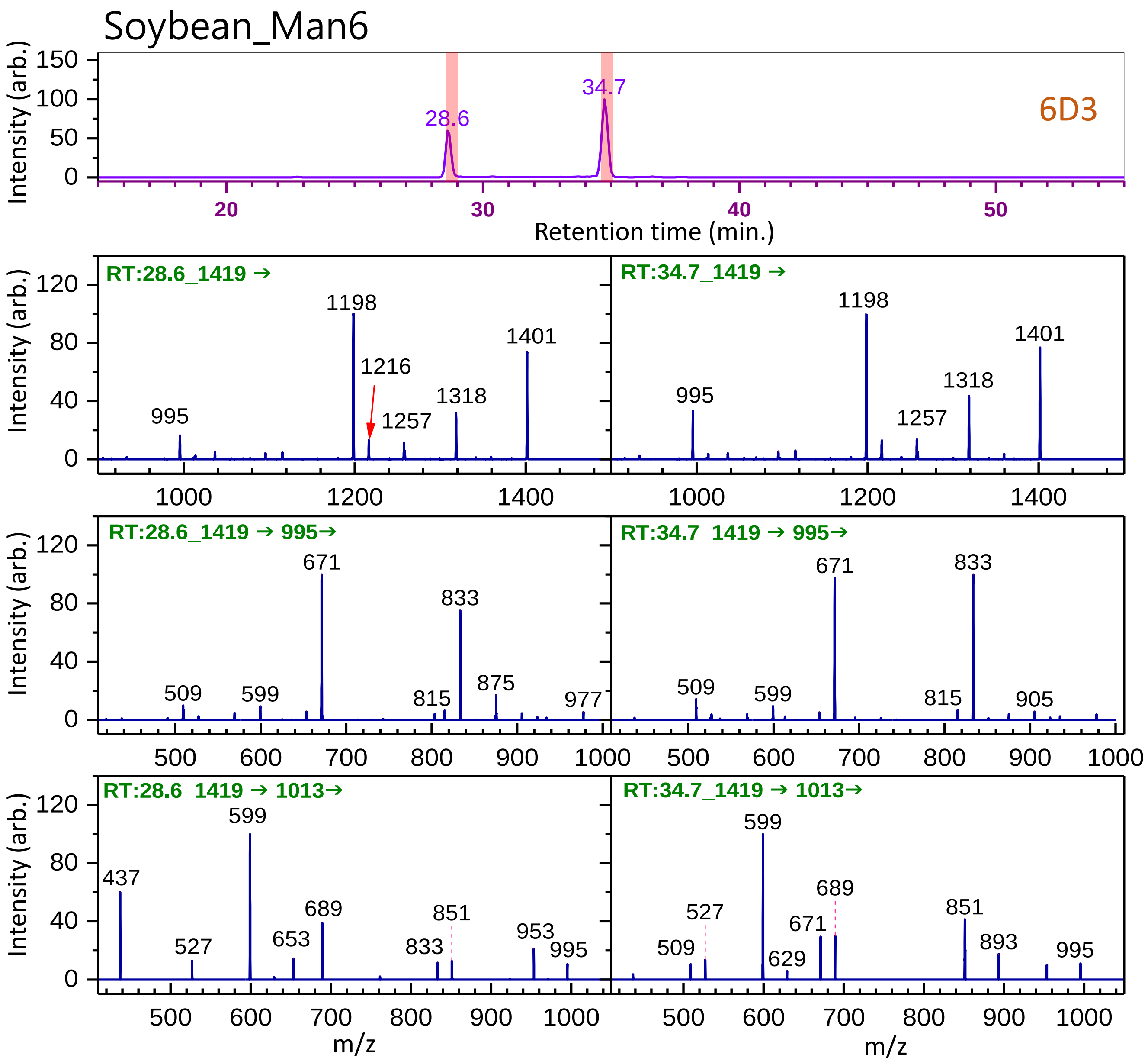

Figure S61. Chromatogram and MS^2 and MS^3 mass spectra of soybean $Man_6GlcNAC_2$.

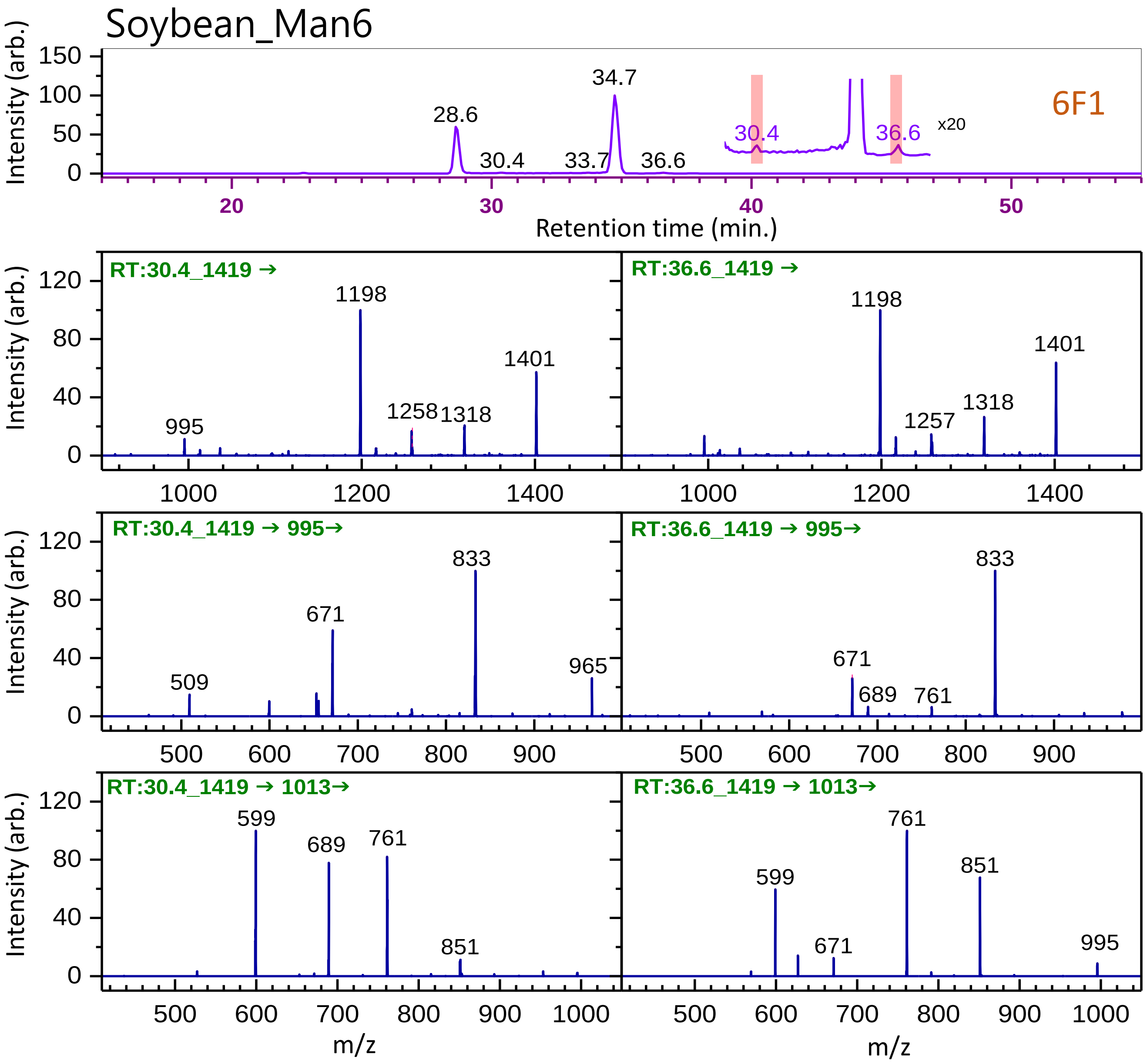

Figure S62. Chromatogram and MS^2 and MS^3 mass spectra of soybean $Man_6GlcNAC_2$.

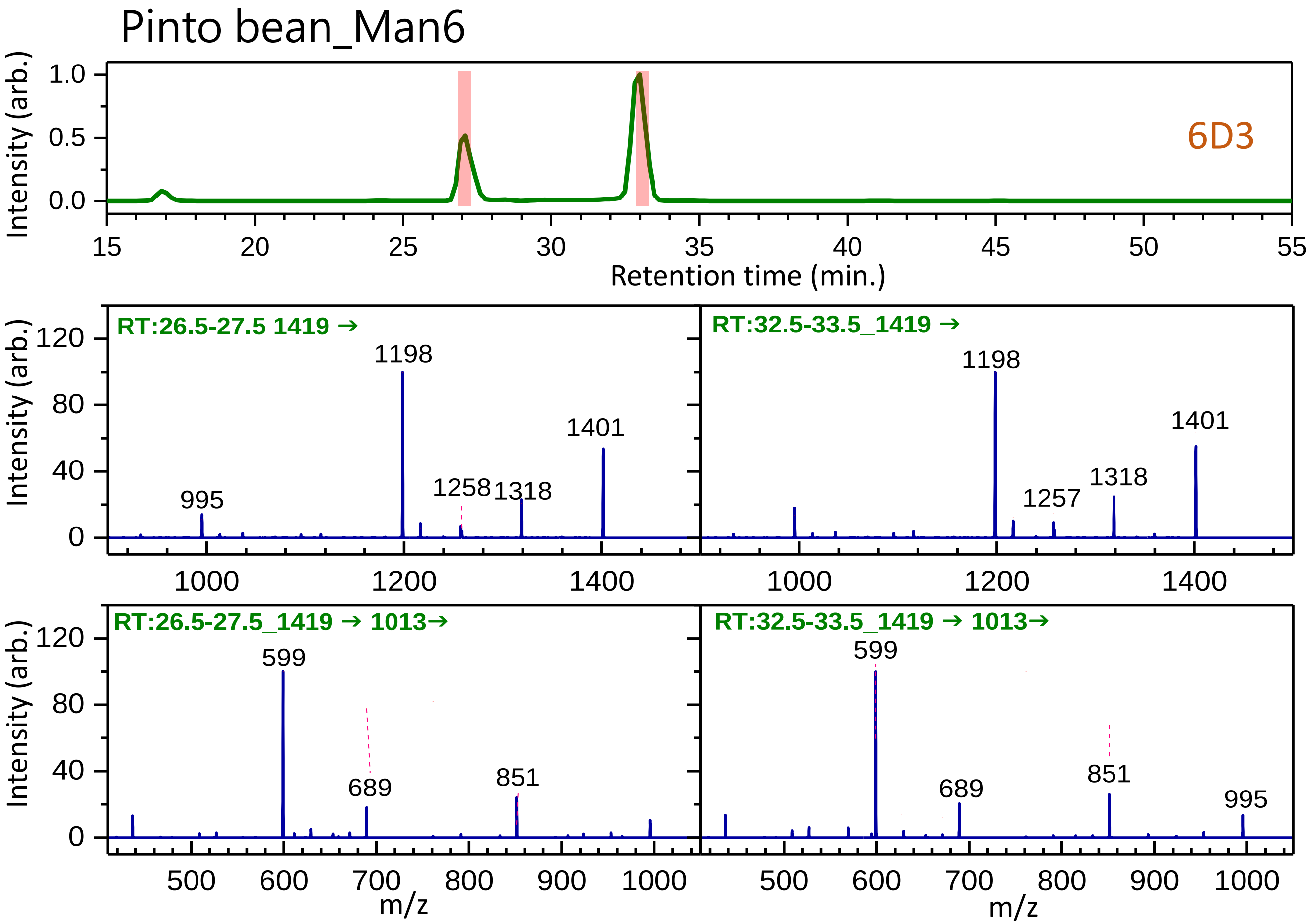

Figure S63. Chromatogram and MS^2 and MS^3 mass spectra of pinto bean $Man_6GlcNAC_2$.

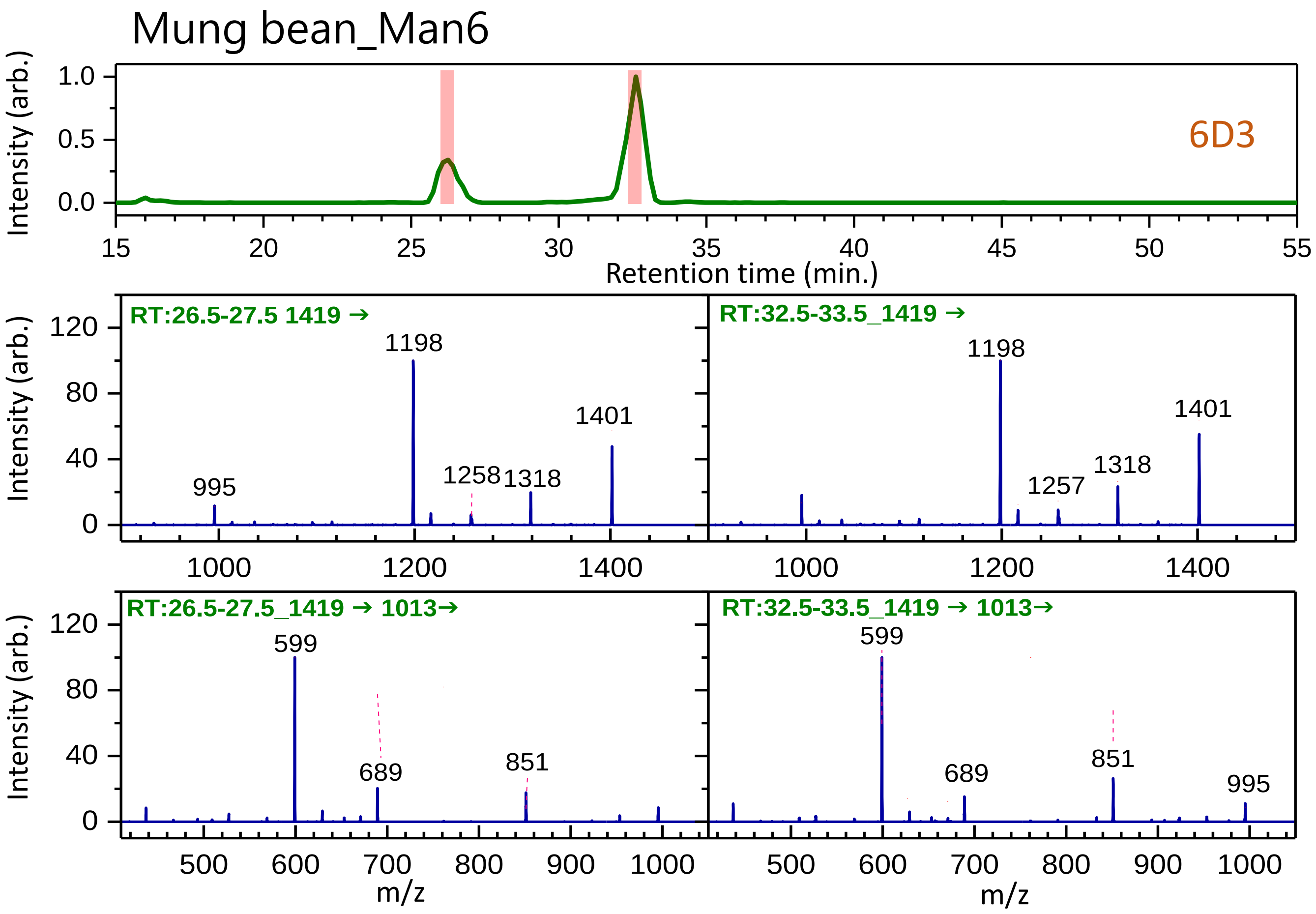

Figure S64. Chromatogram and MS^2 and MS^3 mass spectra of mung bean $Man_6GlcNAC_2$.

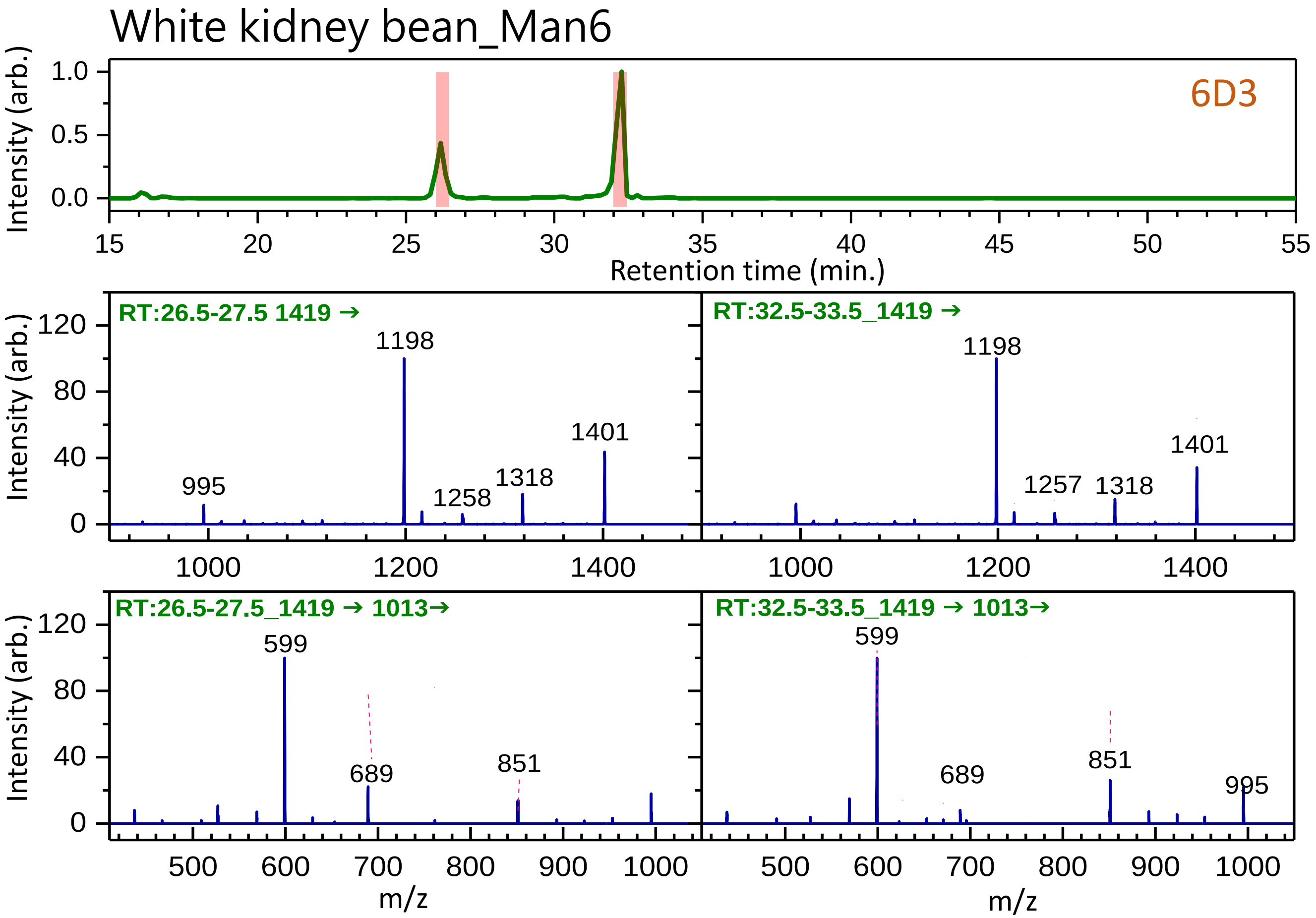

Figure S65. Chromatogram and MS^2 and MS^3 mass spectra of white kidney bean $Man_6GlcNAC_2$.

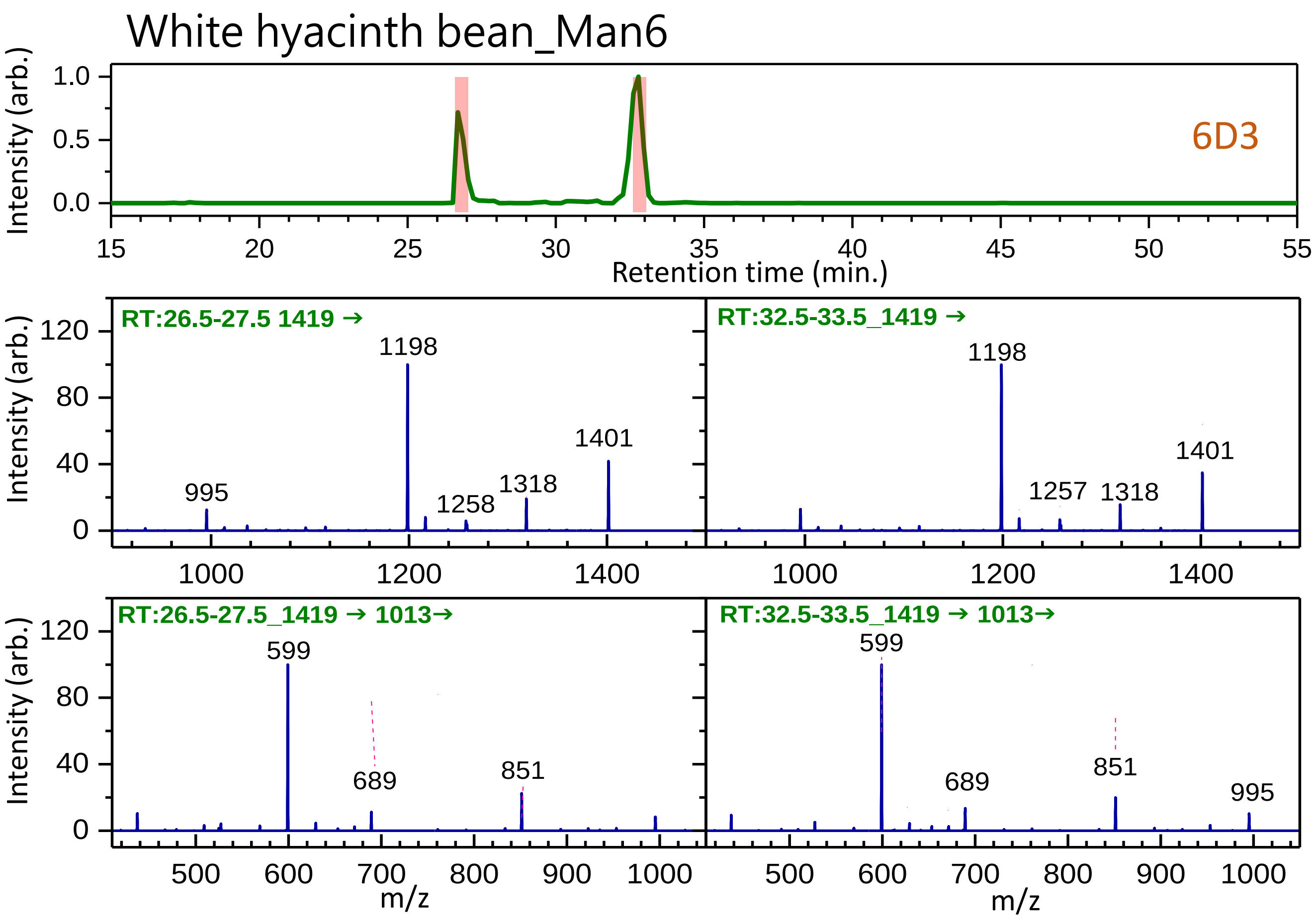

Figure S66. Chromatogram and MS^2 and MS^3 mass spectra of white hyacinth bean $Man_6GlcNAC_2$.

$Man_7GlcNAC_2$

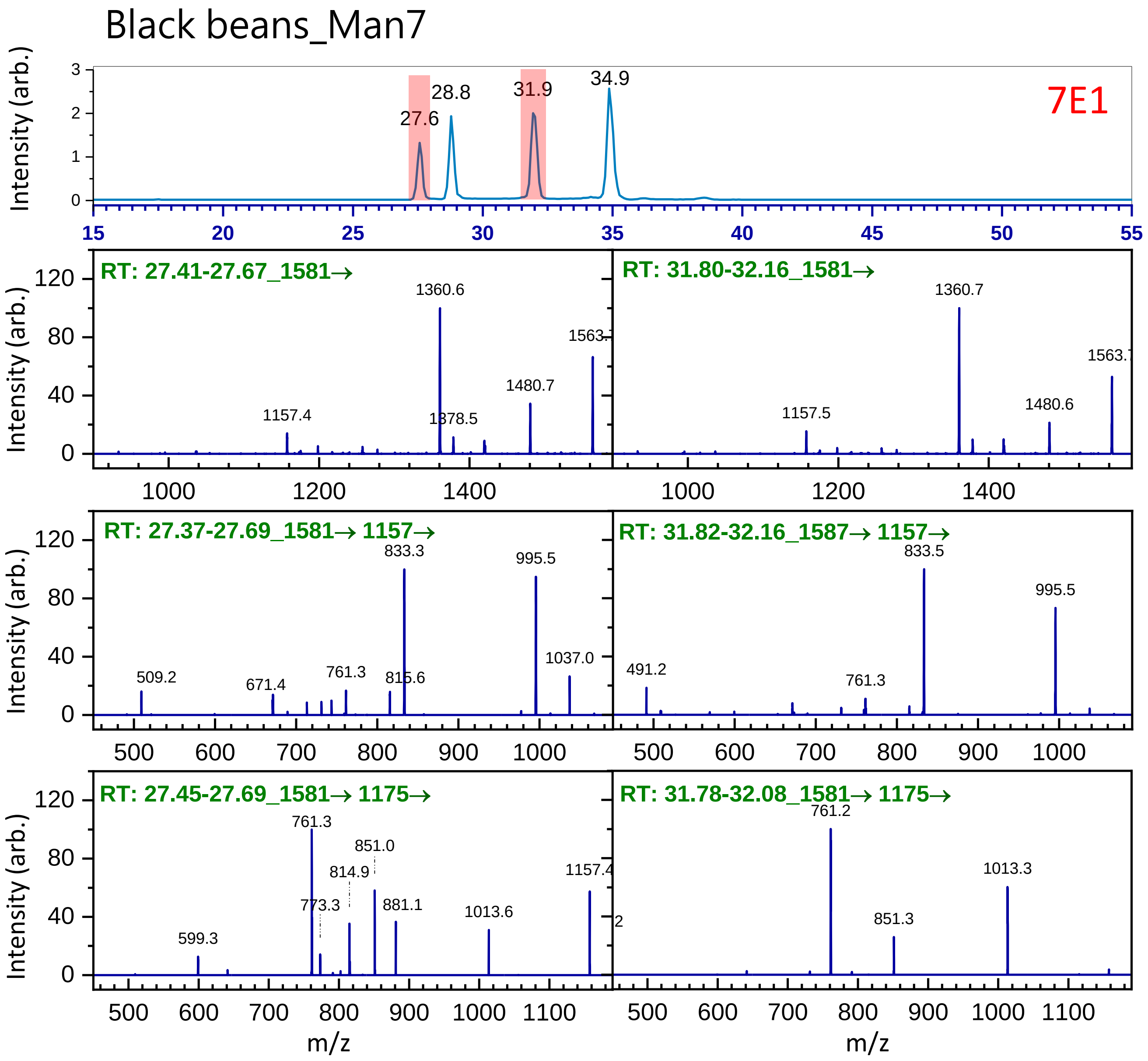

Figure S67. Chromatogram and MS^2 and MS^3 mass spectra of black bean $Man_7GlcNAC_2$.

Black beans_Man7

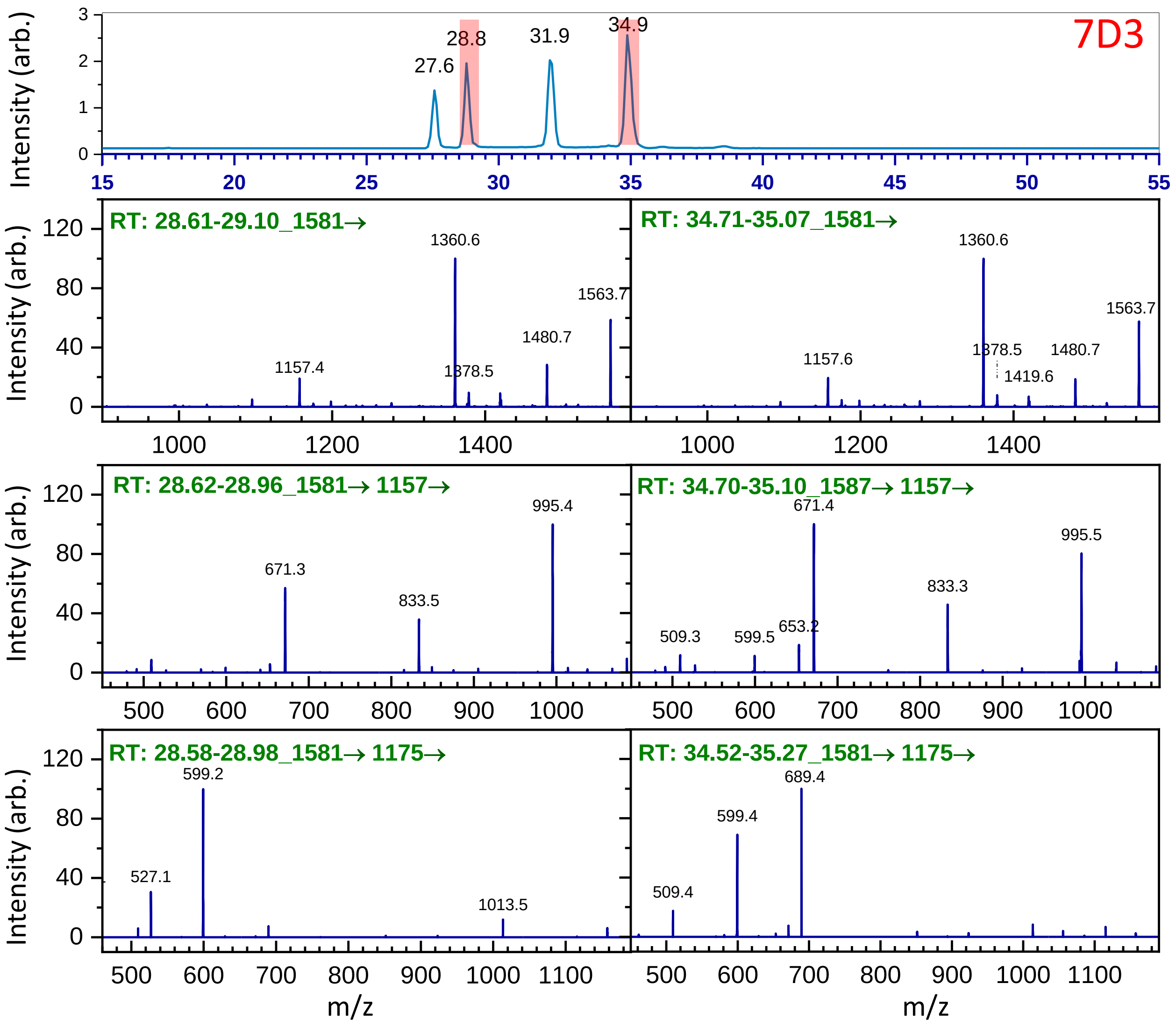

Figure S68. Chromatogram and MS^2 and MS^3 mass spectra of black bean $Man_7GlcNAC_2$.

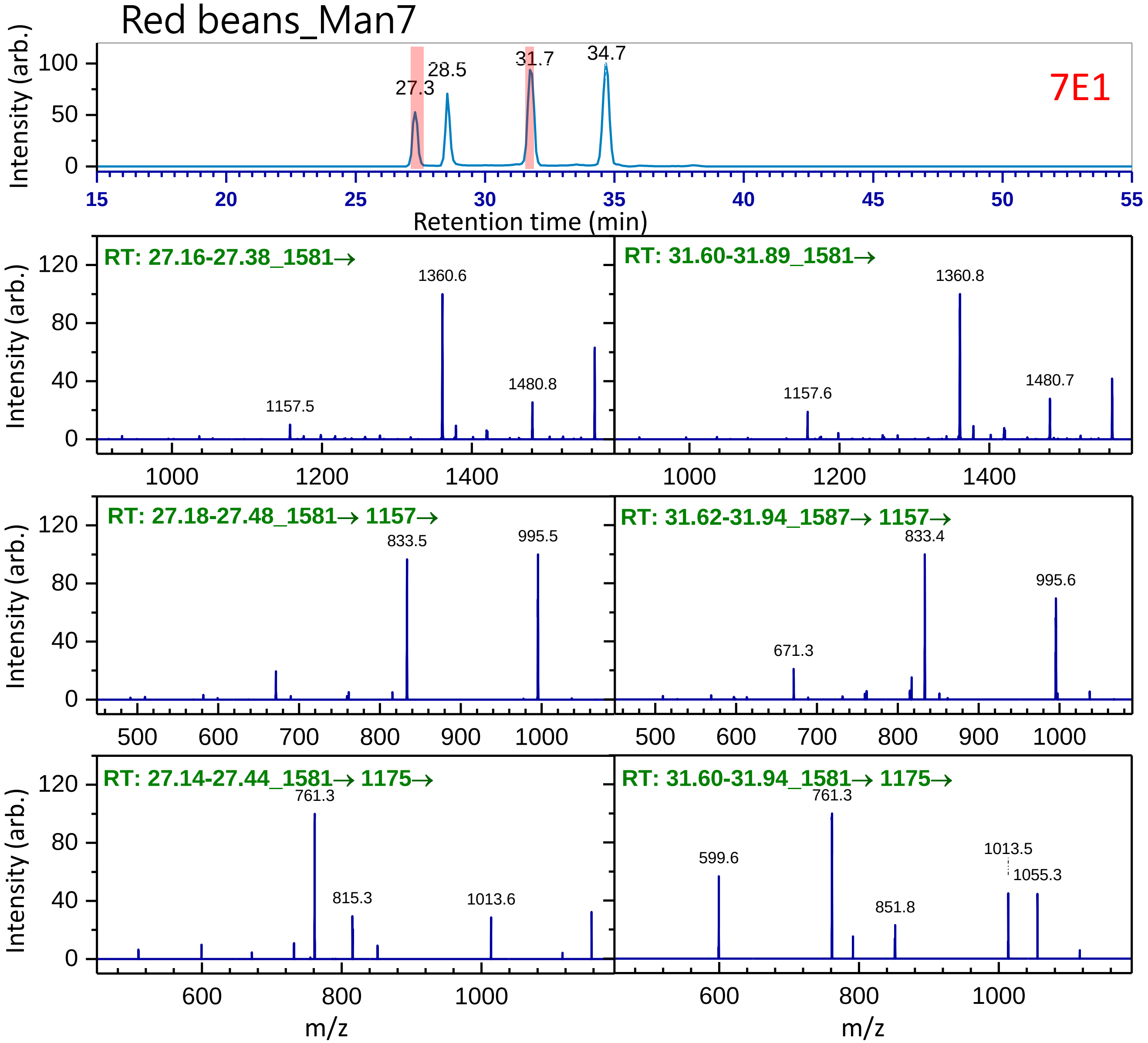

Figure S69. Chromatogram and MS^2 and MS^3 mass spectra of red bean $Man_7GlcNAC_2$.

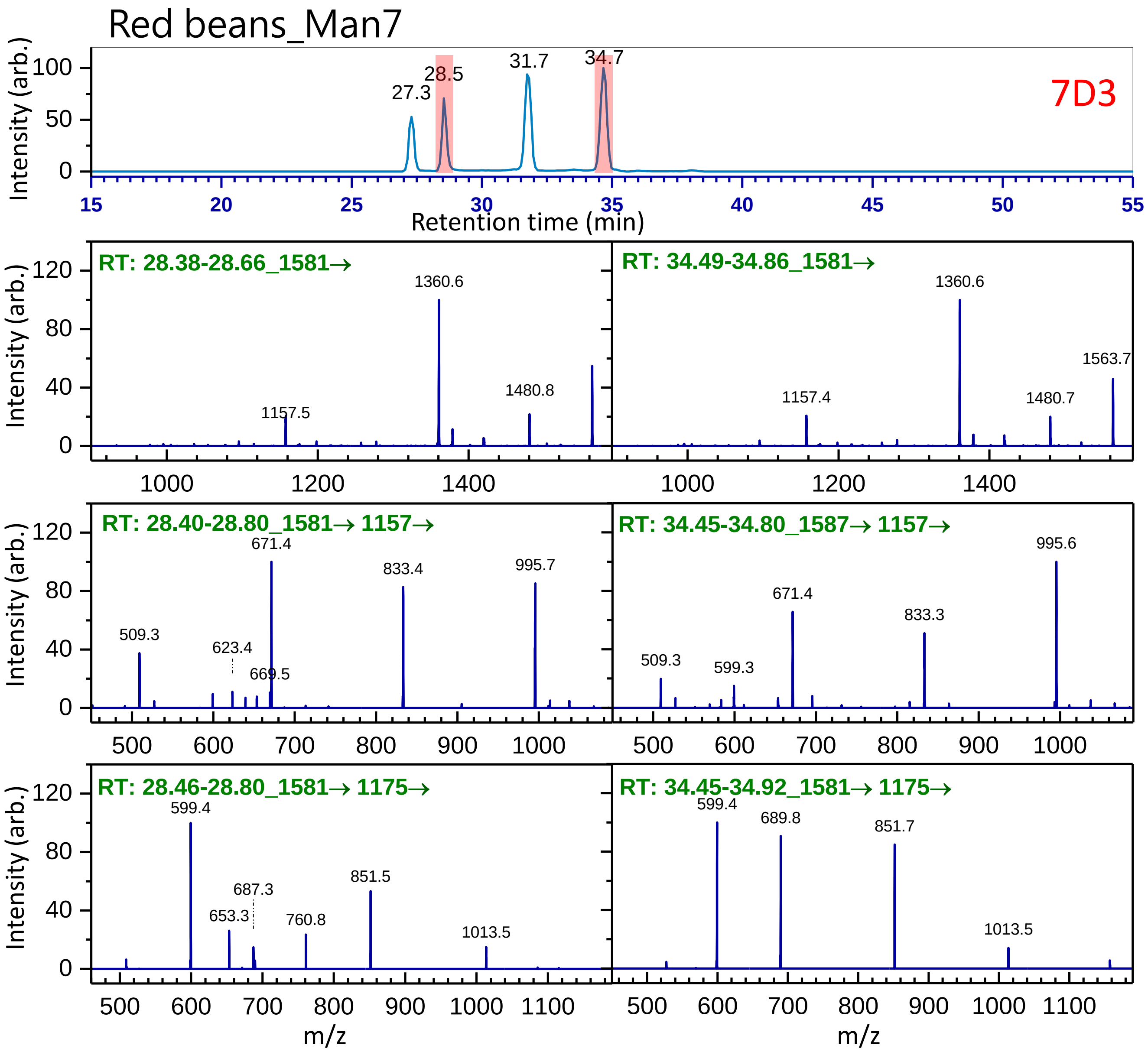

Figure S70. Chromatogram and MS^2 and MS^3 mass spectra of red bean $Man_7GlcNAC_2$.

Pea_Man7

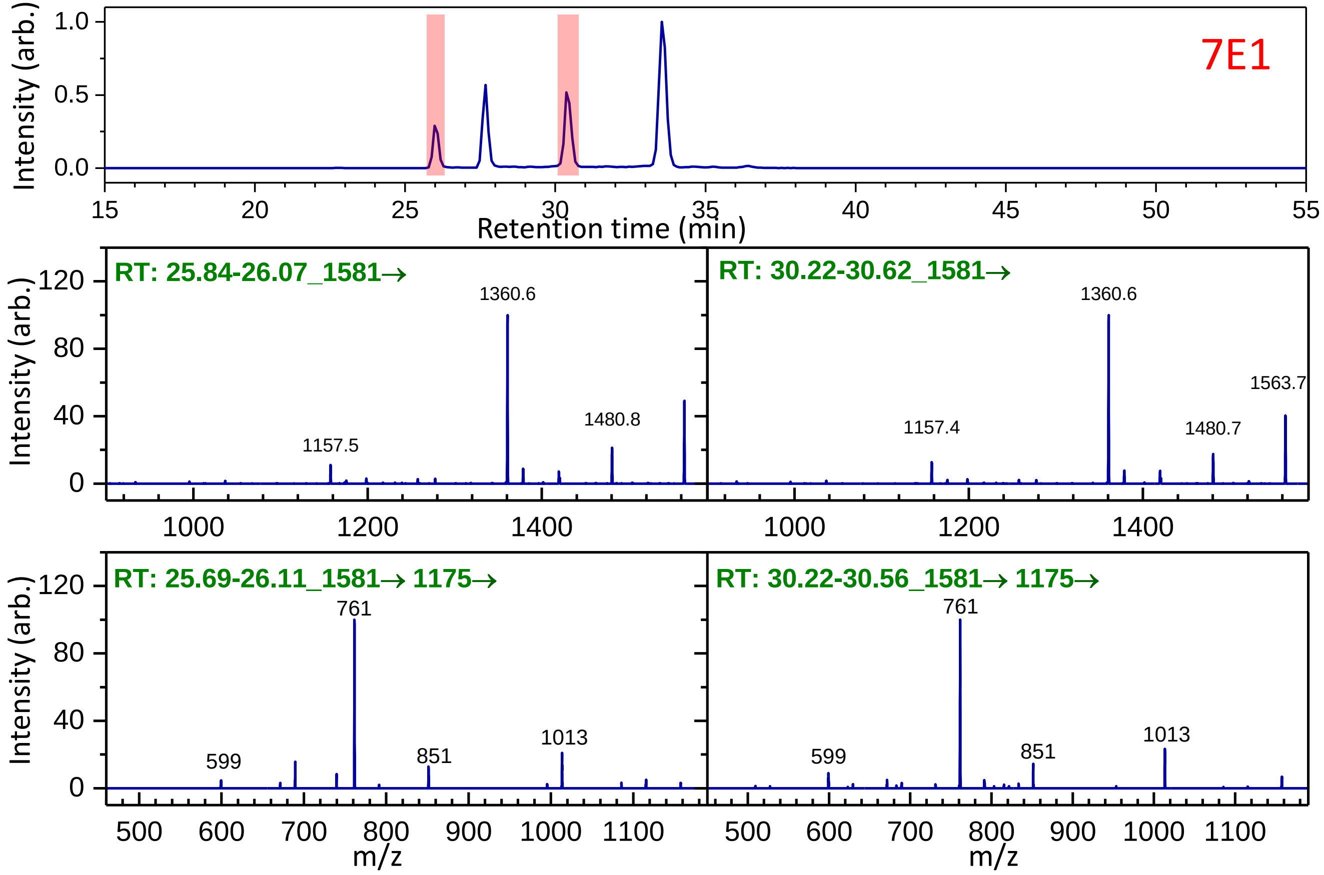

Figure S71. Chromatogram and MS^2 and MS^3 mass spectra of pea $Man_7GlcNAC_2$.

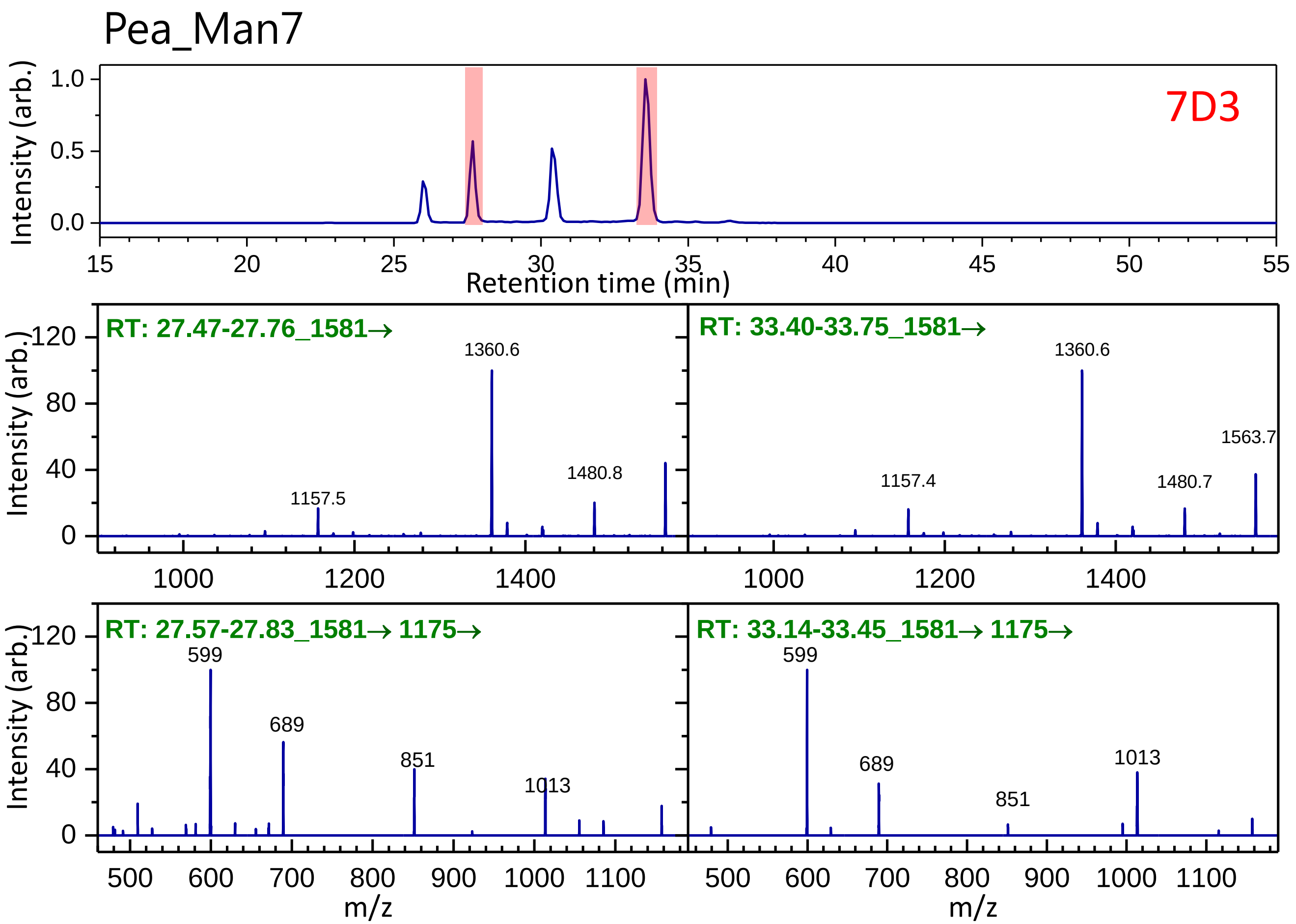

Figure S72. Chromatogram and MS^2 and MS^3 mass spectra of pea $Man_7GlcNAC_2$.

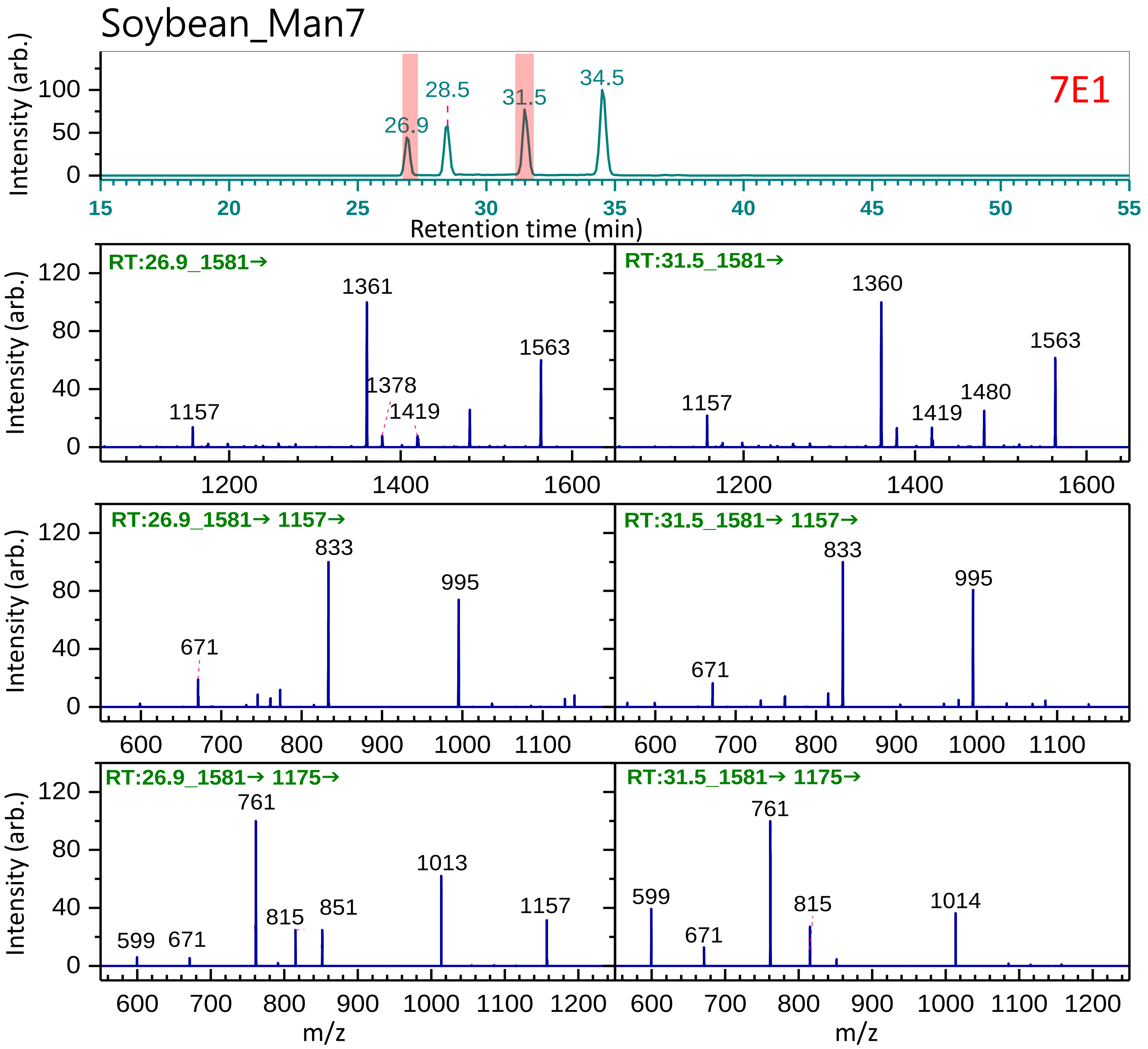

Figure S73. Chromatogram and MS^2 and MS^3 mass spectra of soybean $Man_7GlcNAC_2$.

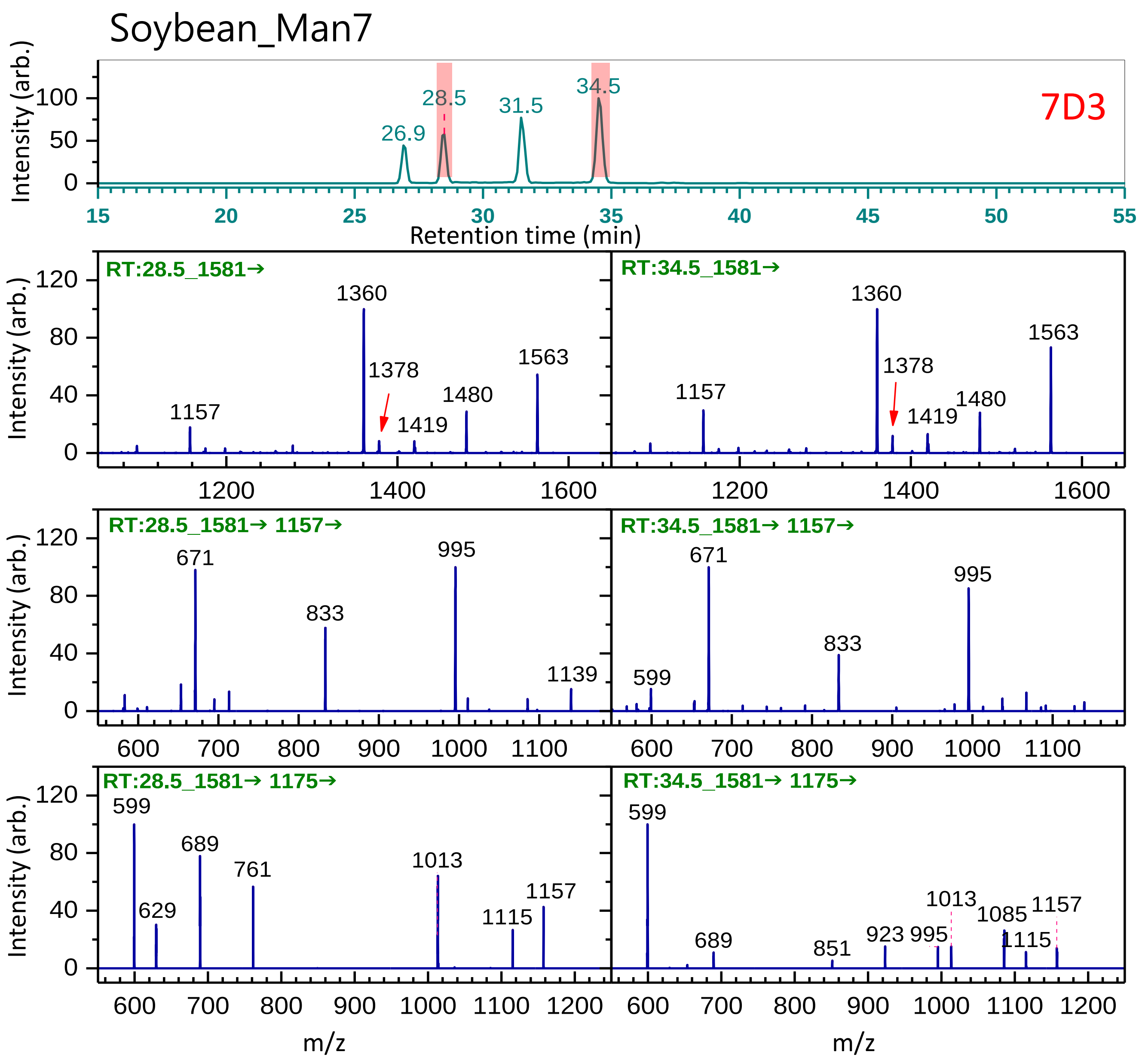

Figure S74. Chromatogram and MS^2 and MS^3 mass spectra of soybean $Man_7GlcNAC_2$.

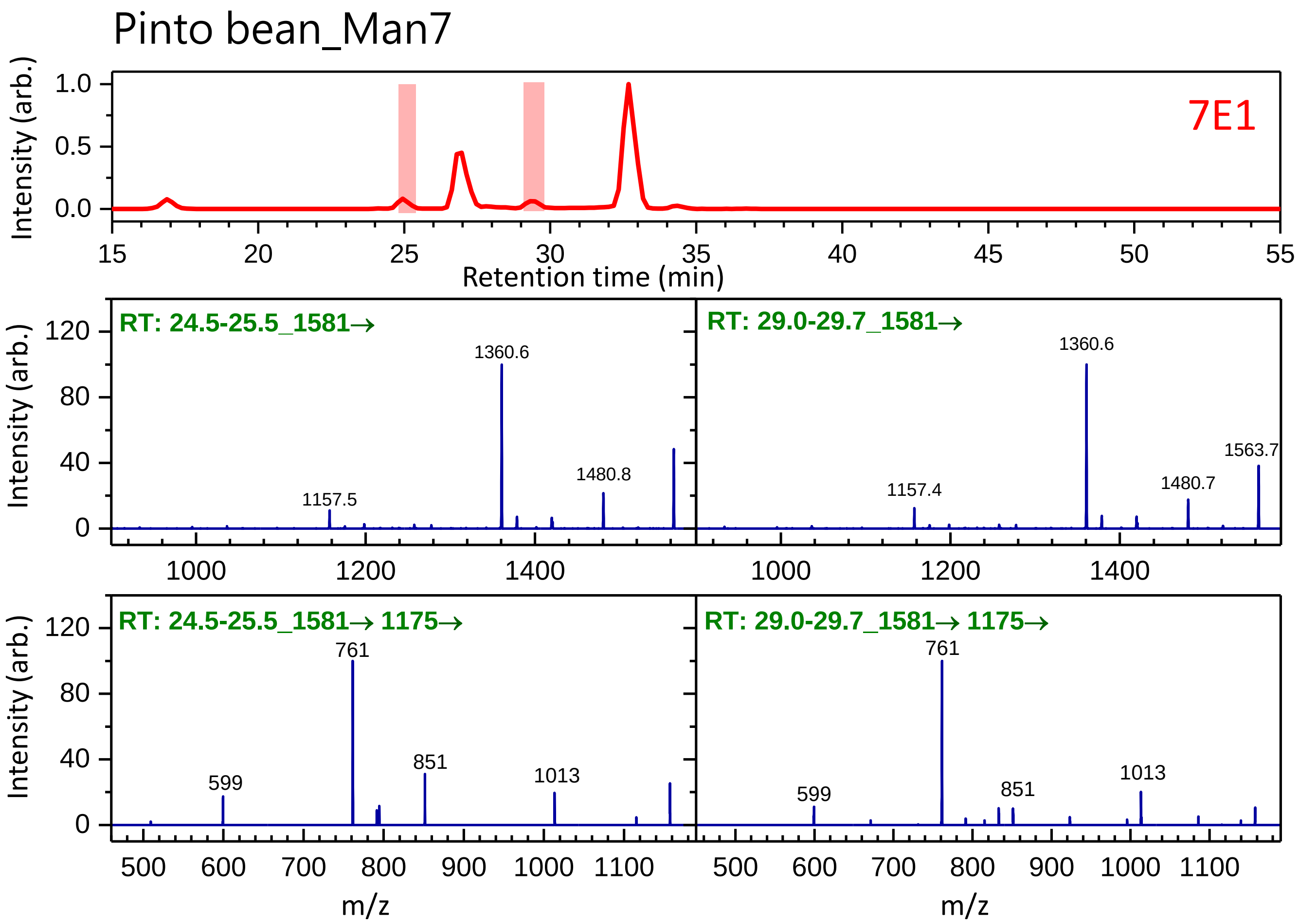

Figure S75. Chromatogram and MS^2 and MS^3 mass spectra of pinto bean $Man_7GlcNAC_2$.

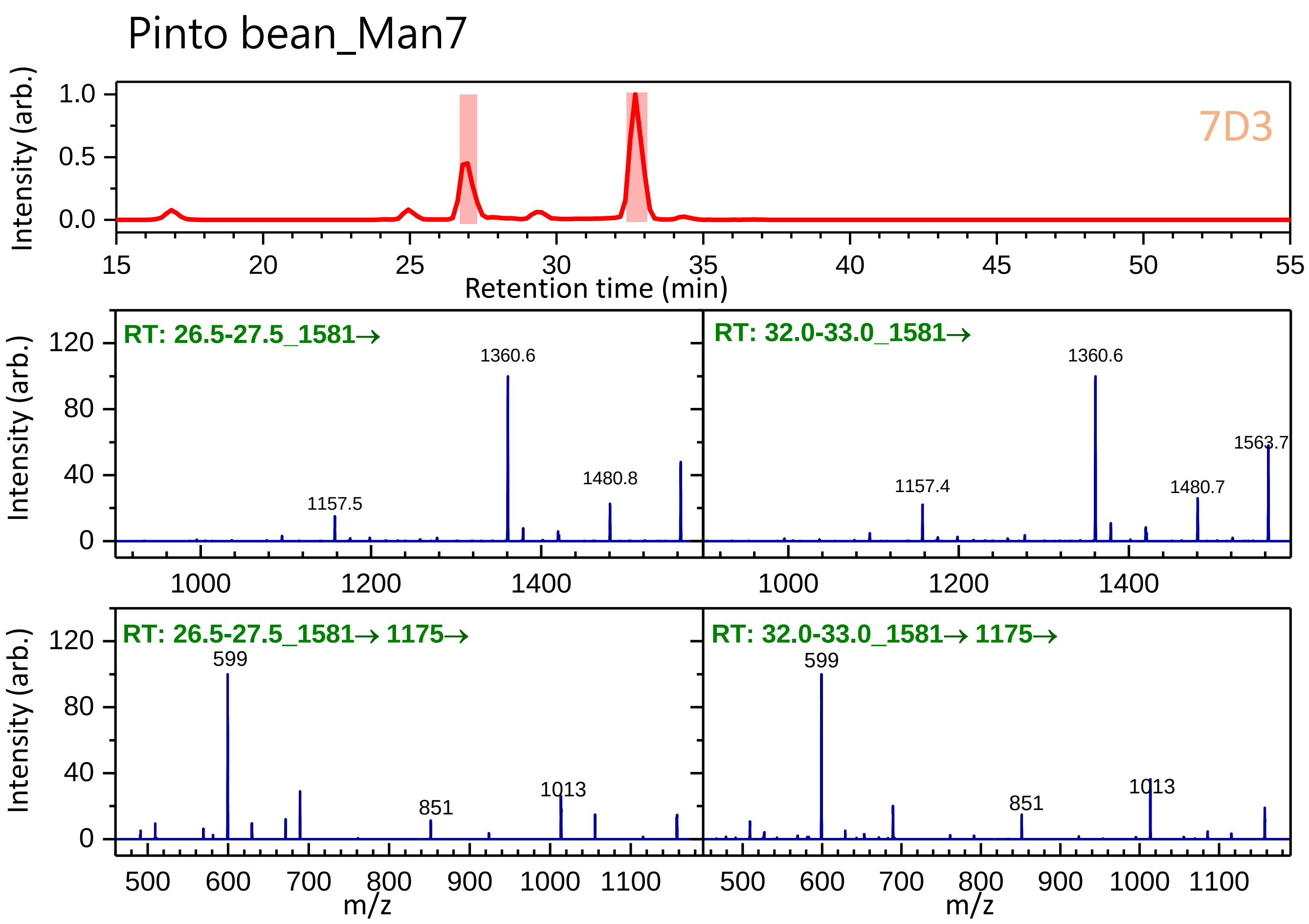

Figure S76. Chromatogram and MS^2 and MS^3 mass spectra of pinto bean $Man_7GlcNAC_2$.

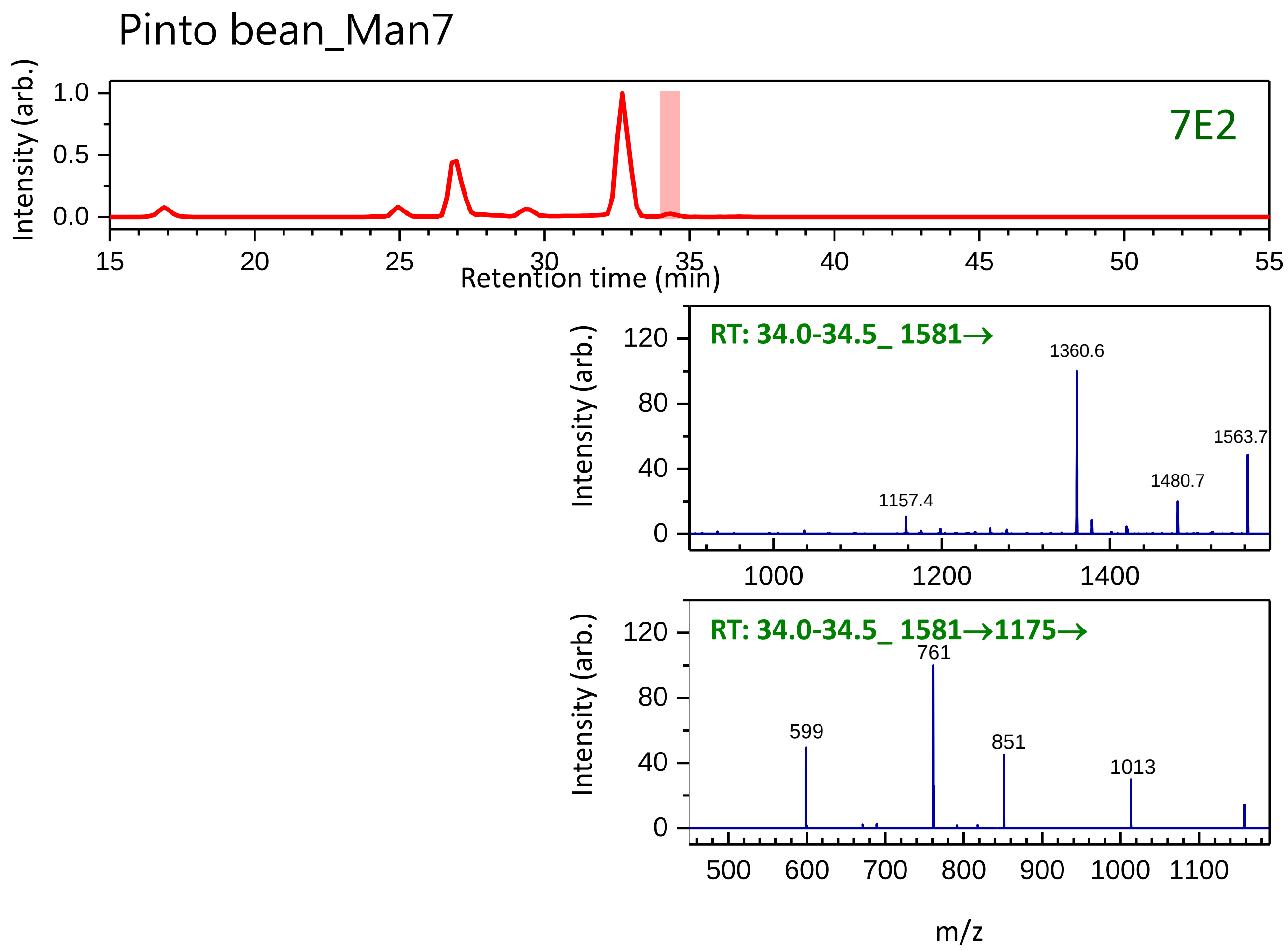

Figure S77. Chromatogram and MS^2 and MS^3 mass spectra of pinto bean $Man_7GlcNAC_2$.

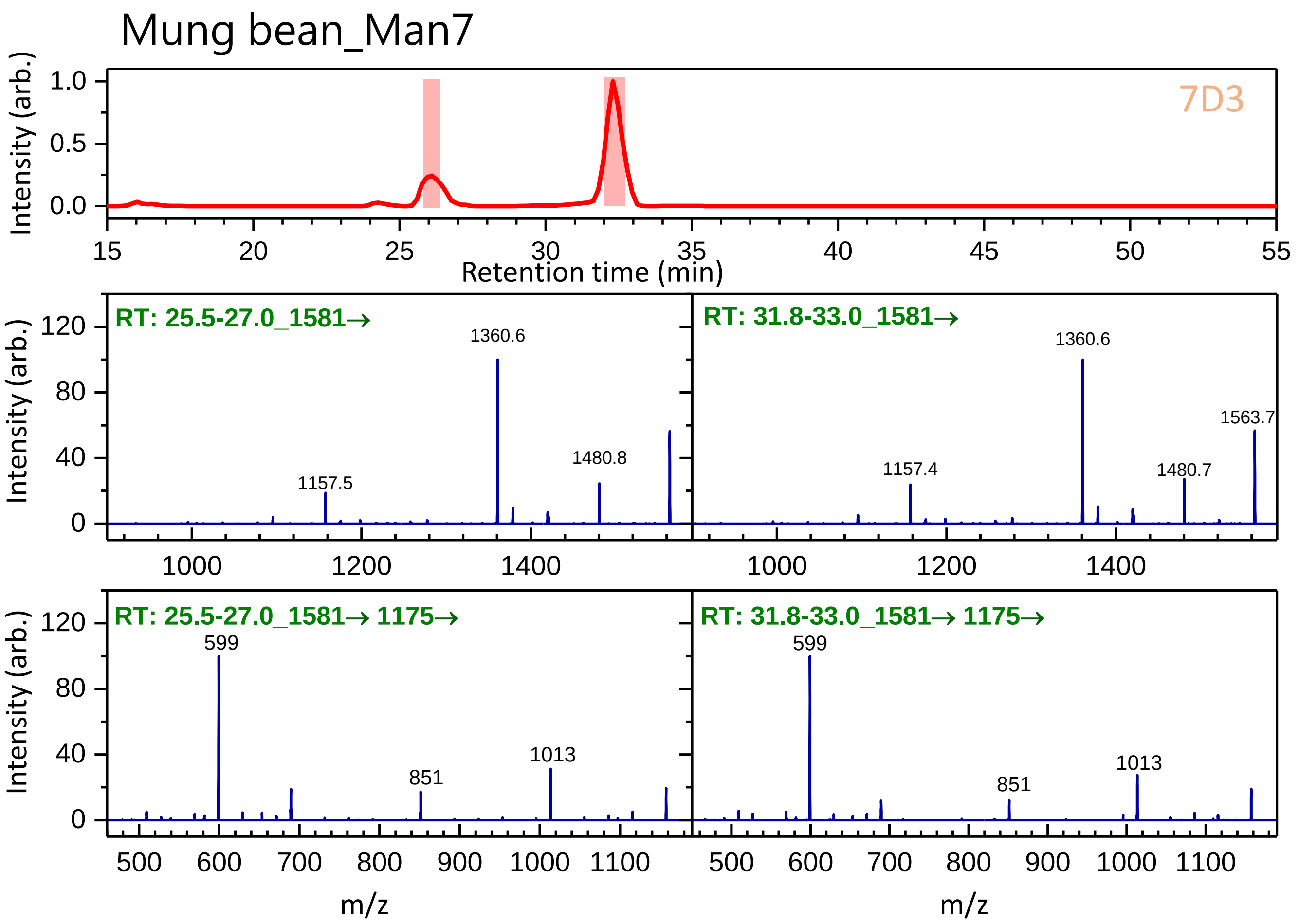

Figure S78. Chromatogram and MS^2 and MS^3 mass spectra of mung bean $Man_7GlcNAC_2$.

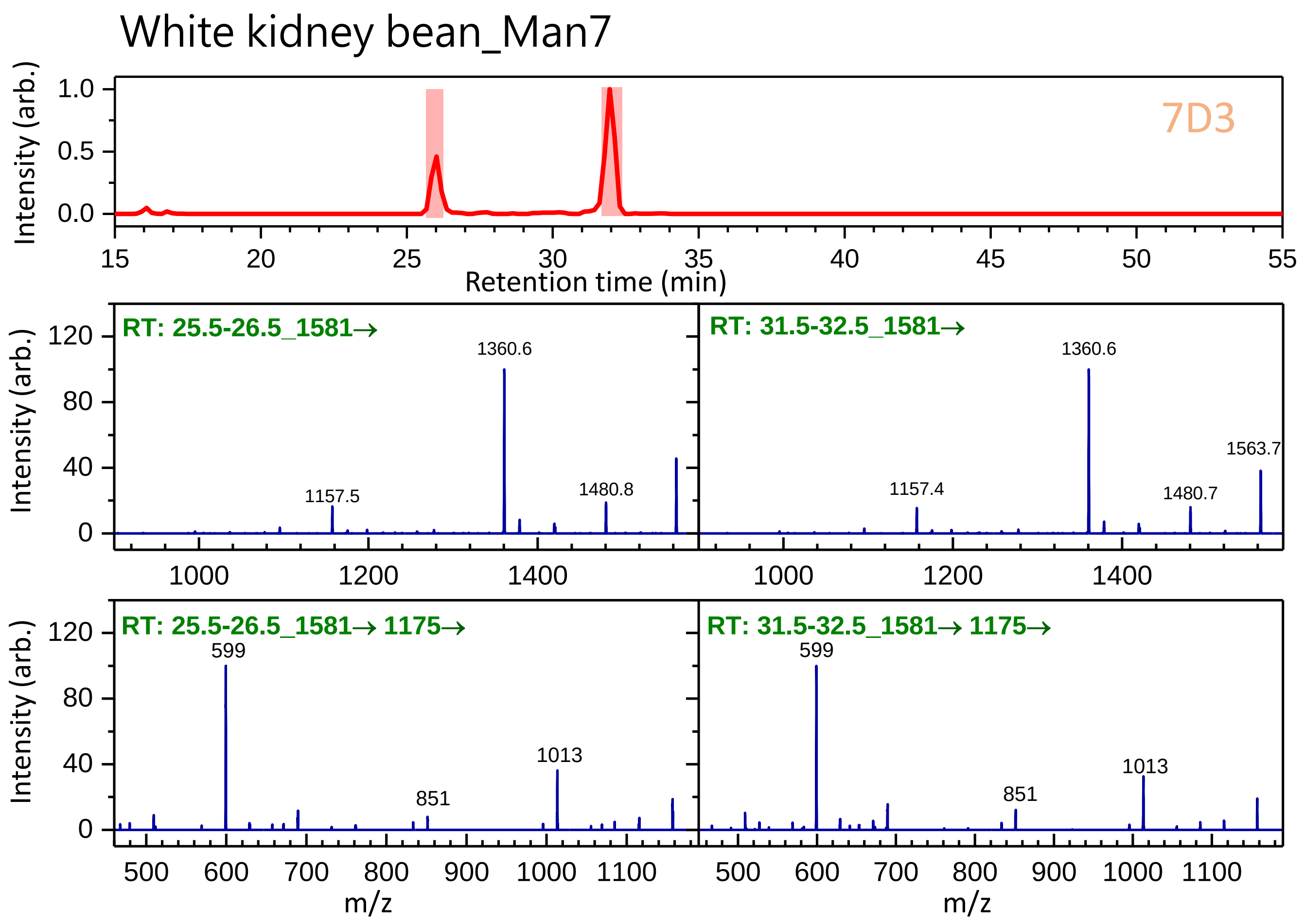

Figure S79. Chromatogram and MS^2 and MS^3 mass spectra of white kidney bean $Man_7GlcNAC_2$.

White hyacinth bean _Man 7

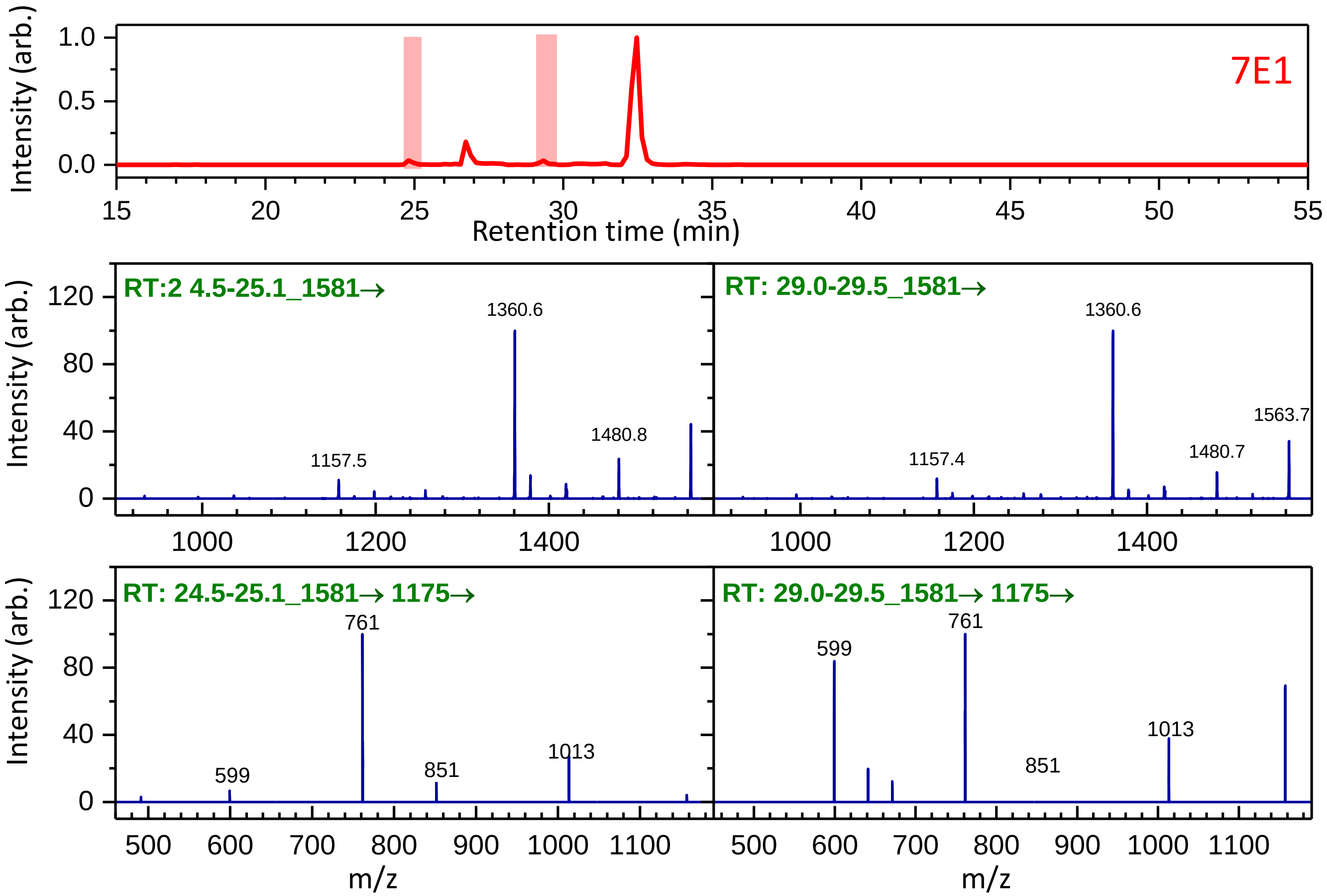

Figure S80. Chromatogram and MS^2 and MS^3 mass spectra of white hyacinth bean $Man_7GlcNAC_2$.

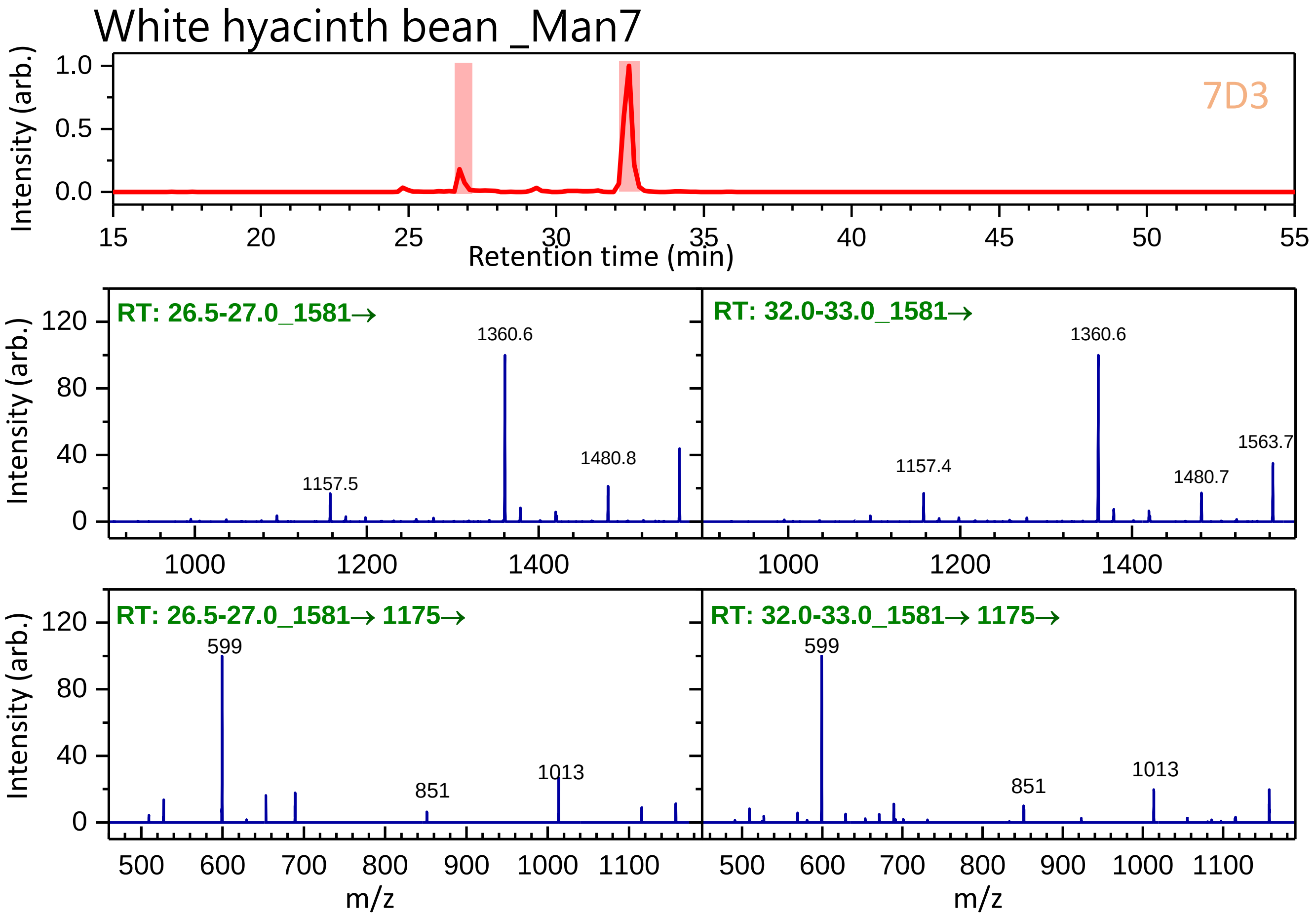

Figure S81. Chromatogram and MS^2 and MS^3 mass spectra of white hyacinth bean $Man_7GlcNAC_2$.

$Man_8GlcNAC_2$

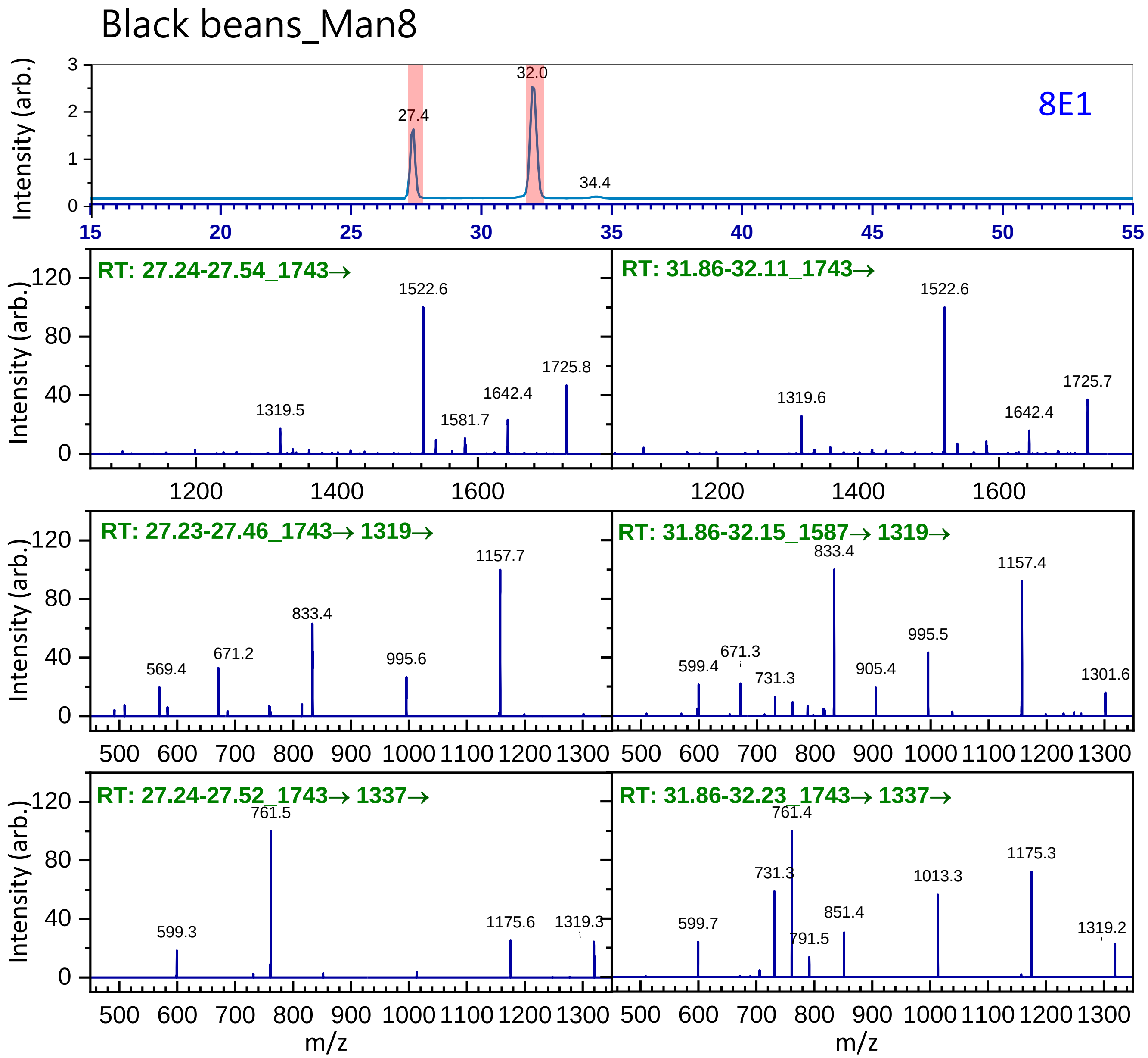

Figure S82. Chromatogram and MS^2 and MS^3 mass spectra of black bean $Man_8GlcNAC_2$.

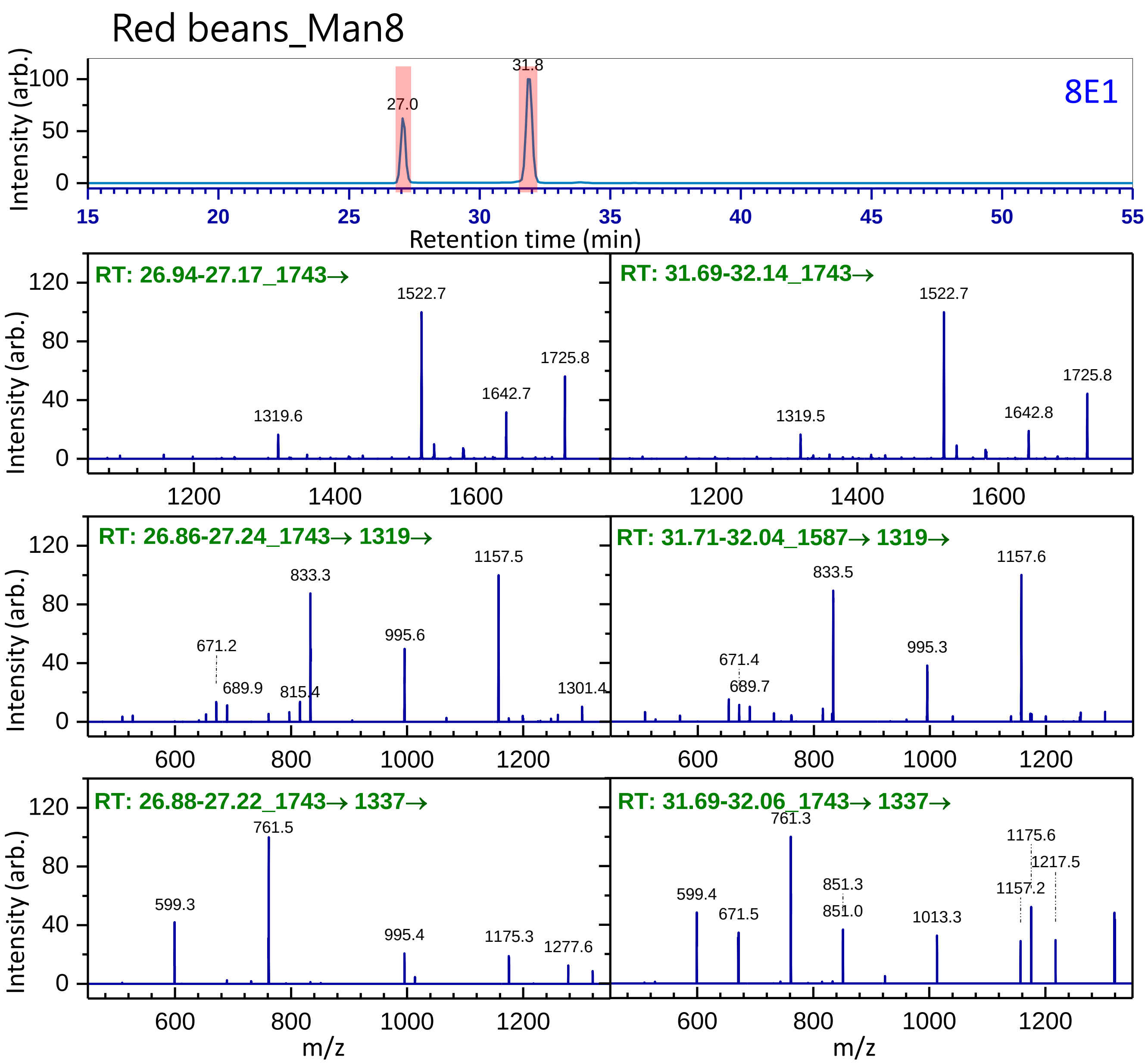

Figure S83. Chromatogram and MS^2 and MS^3 mass spectra of red bean $Man_8GlcNAC_2$.

Soybeans_Man8

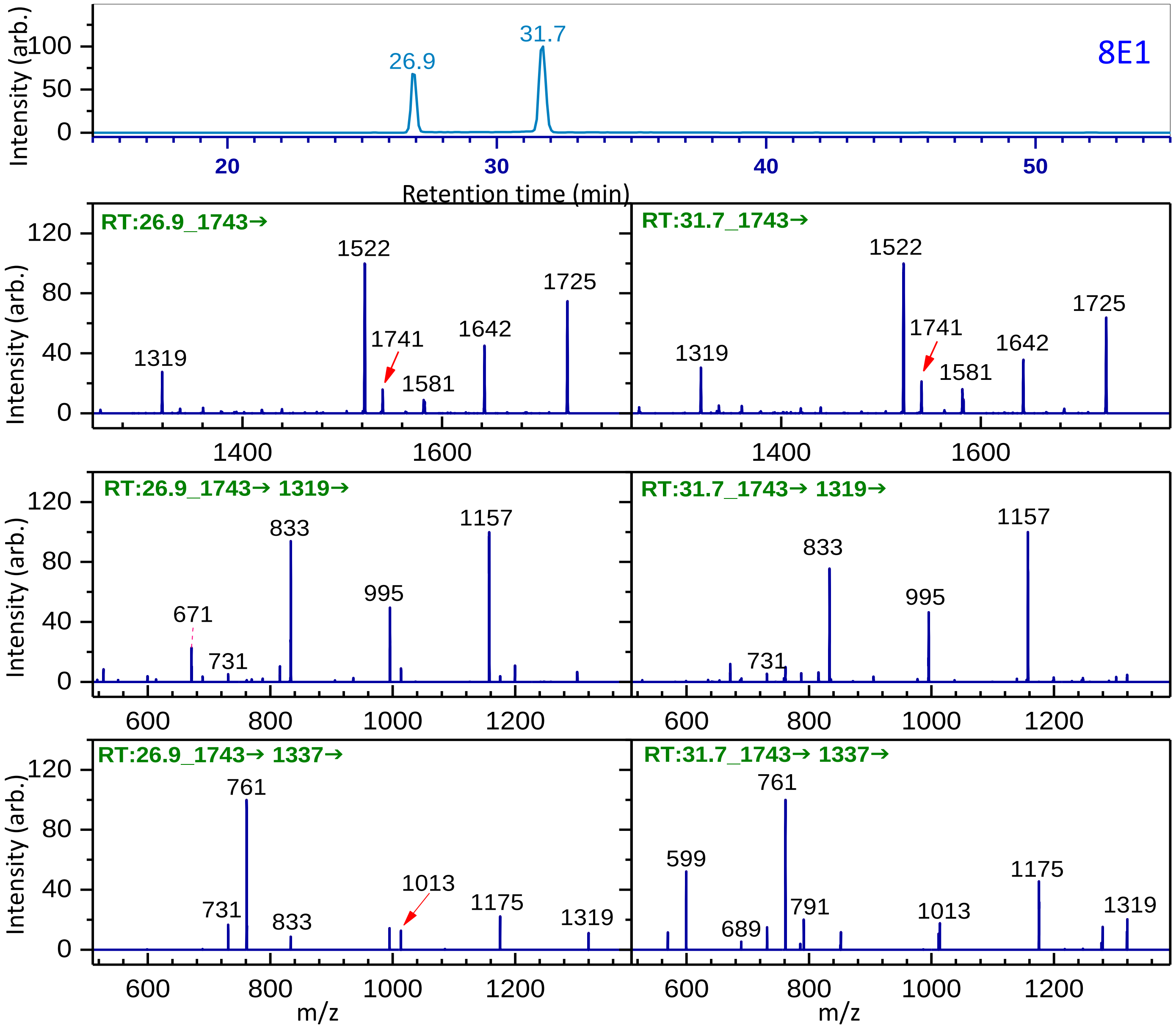

Figure S84. Chromatogram and MS^2 and MS^3 mass spectra of soybean $Man_8GlcNAC_2$.

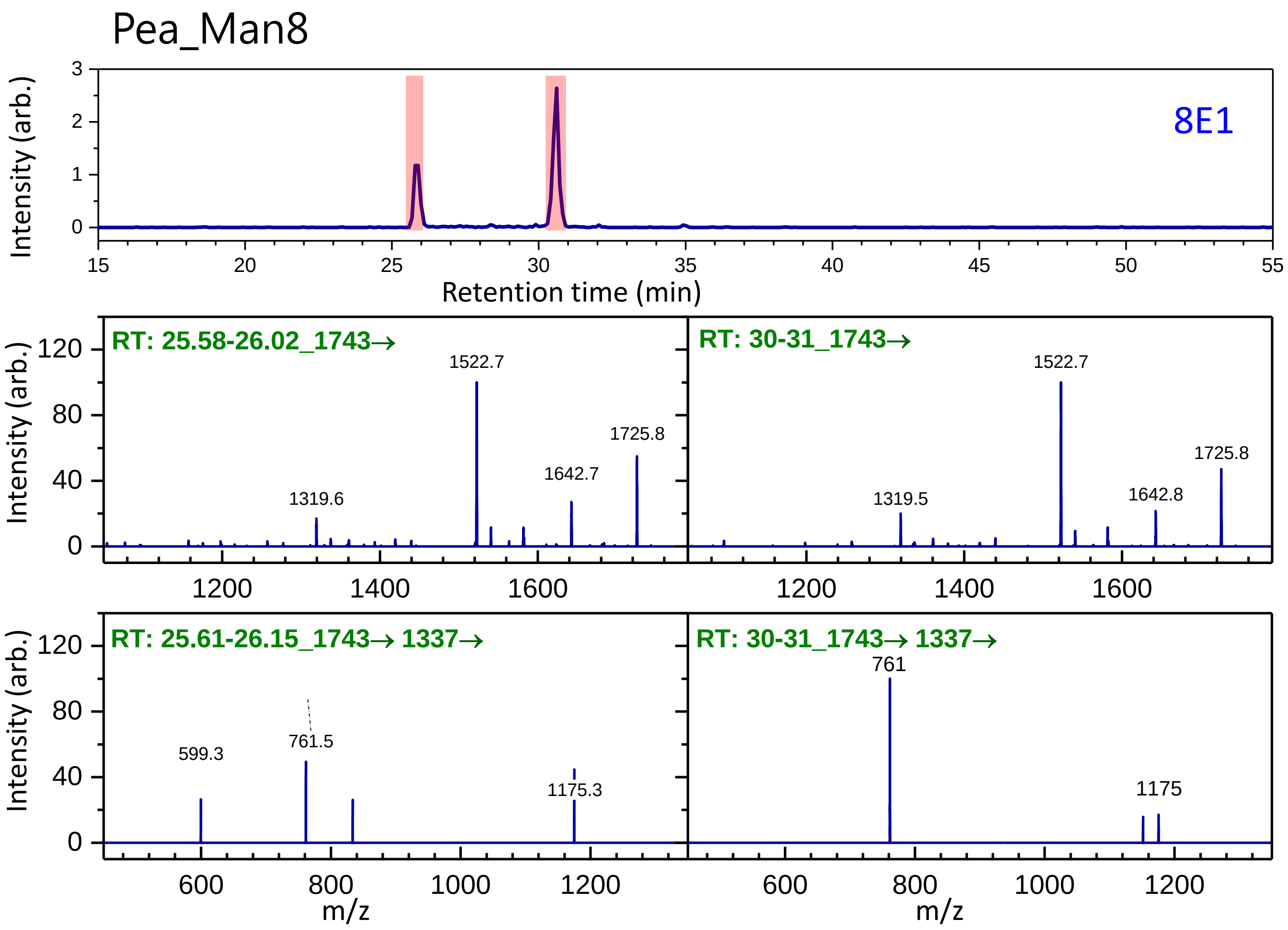

Figure S85. Chromatogram and MS^2 and MS^3 mass spectra of pea $Man_8GlcNAC_2$.

Pinto bean_Man8

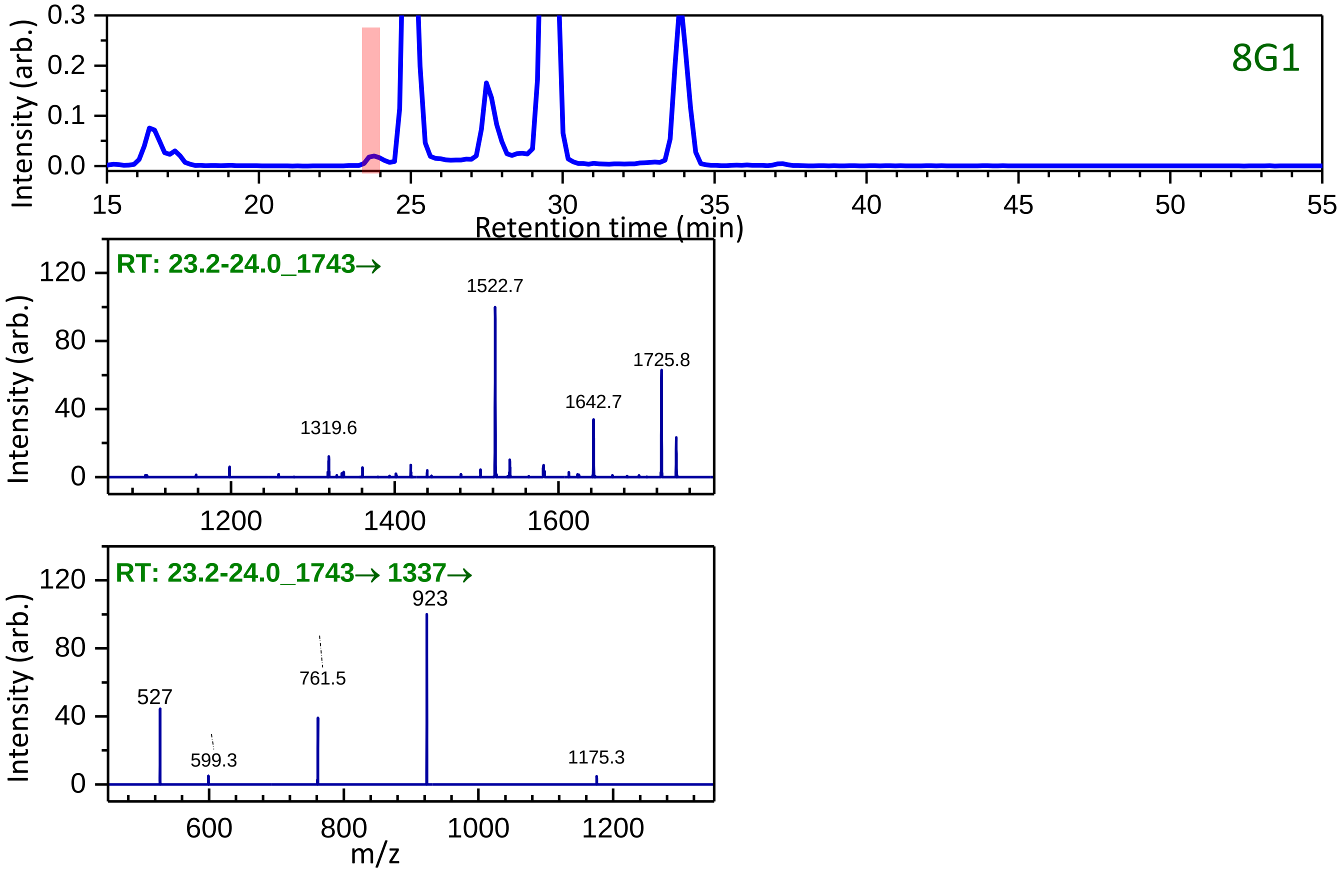

Figure S86. Chromatogram and MS^2 and MS^3 mass spectra of pinto bean $Man_8GlcNAC_2$.

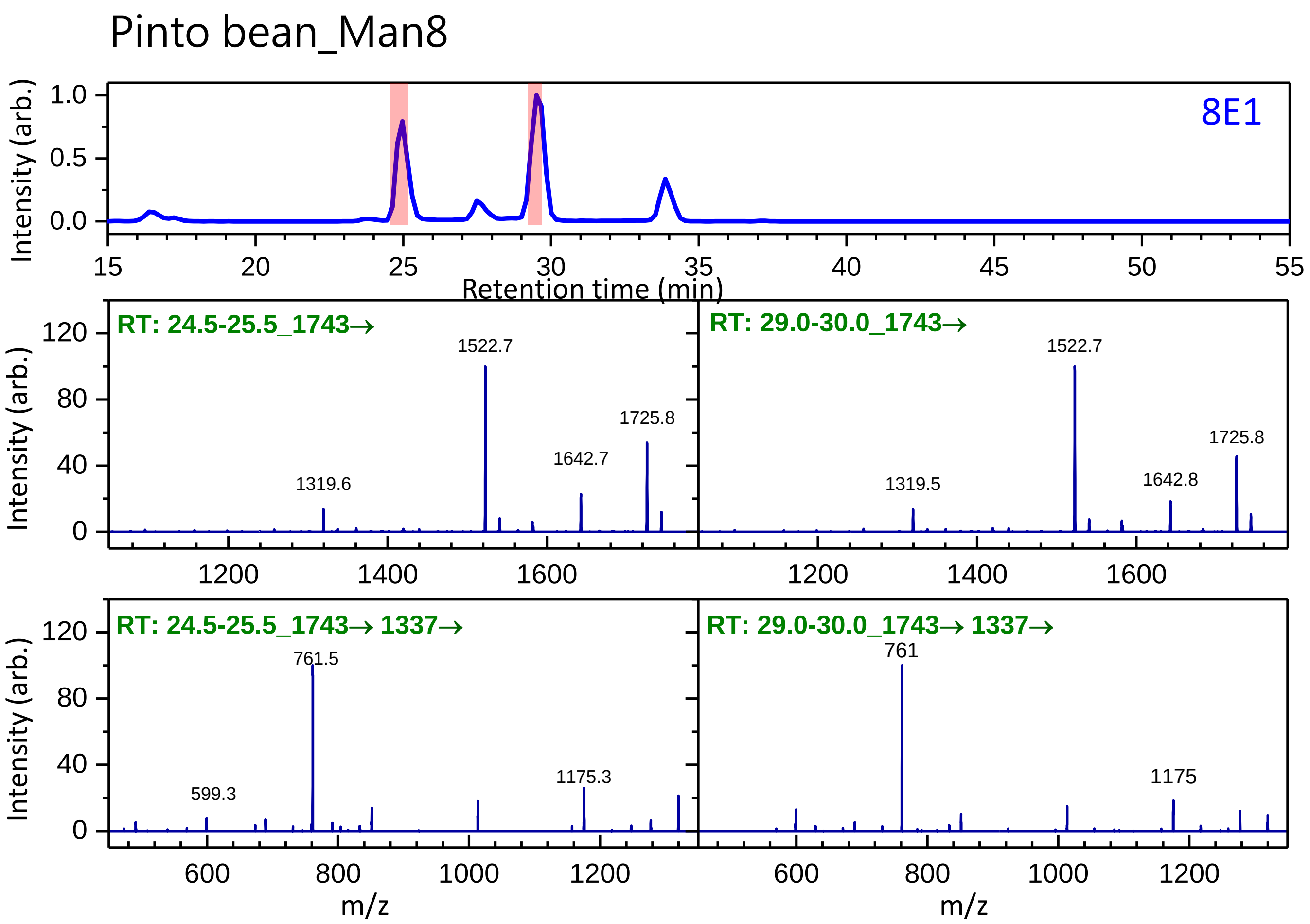

Figure S87. Chromatogram and MS^2 and MS^3 mass spectra of pinto bean $Man_8GlcNAC_2$.

Pinto bean_Man8

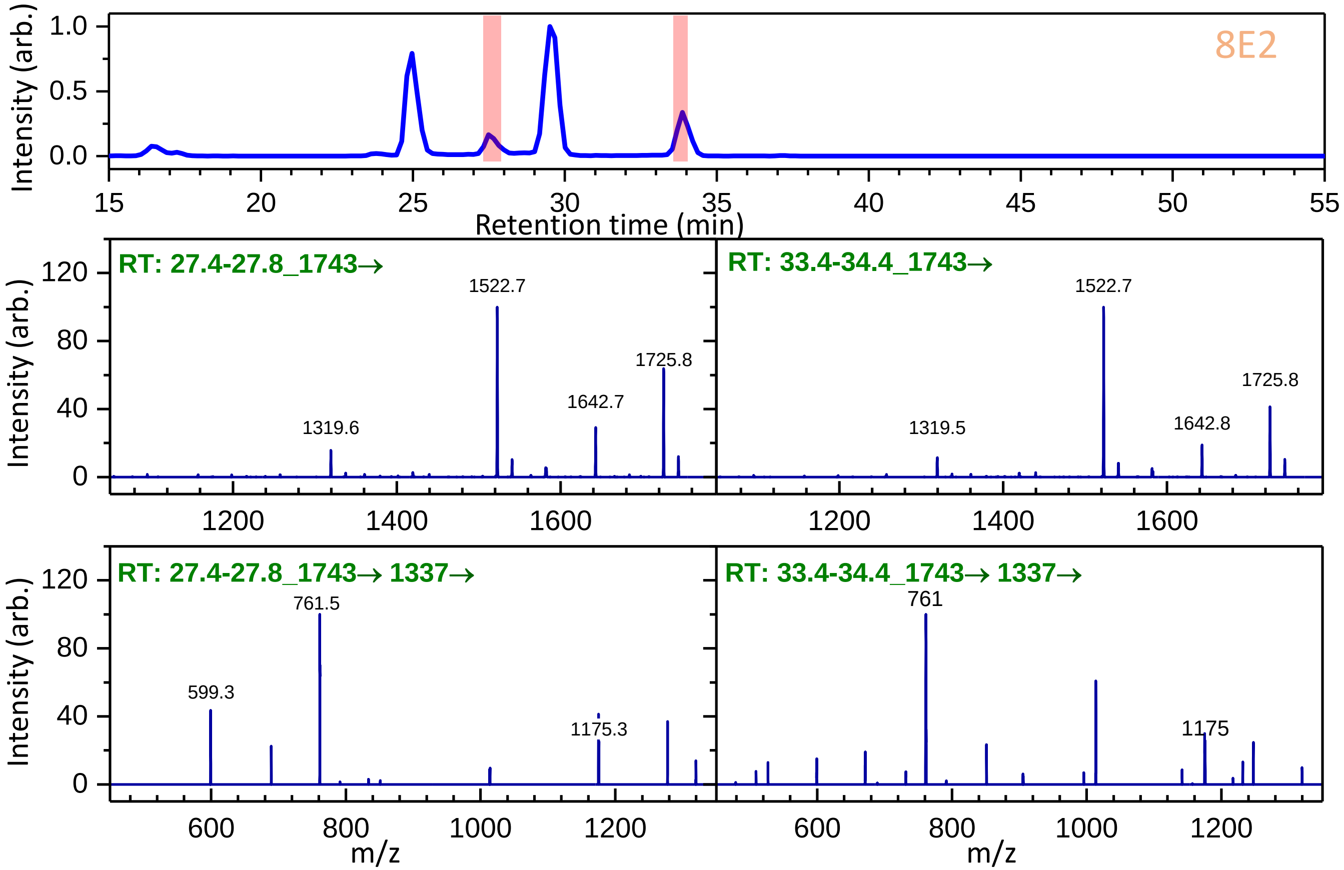

Figure S88. Chromatogram and MS^2 and MS^3 mass spectra of pinto bean $Man_8GlcNAC_2$.

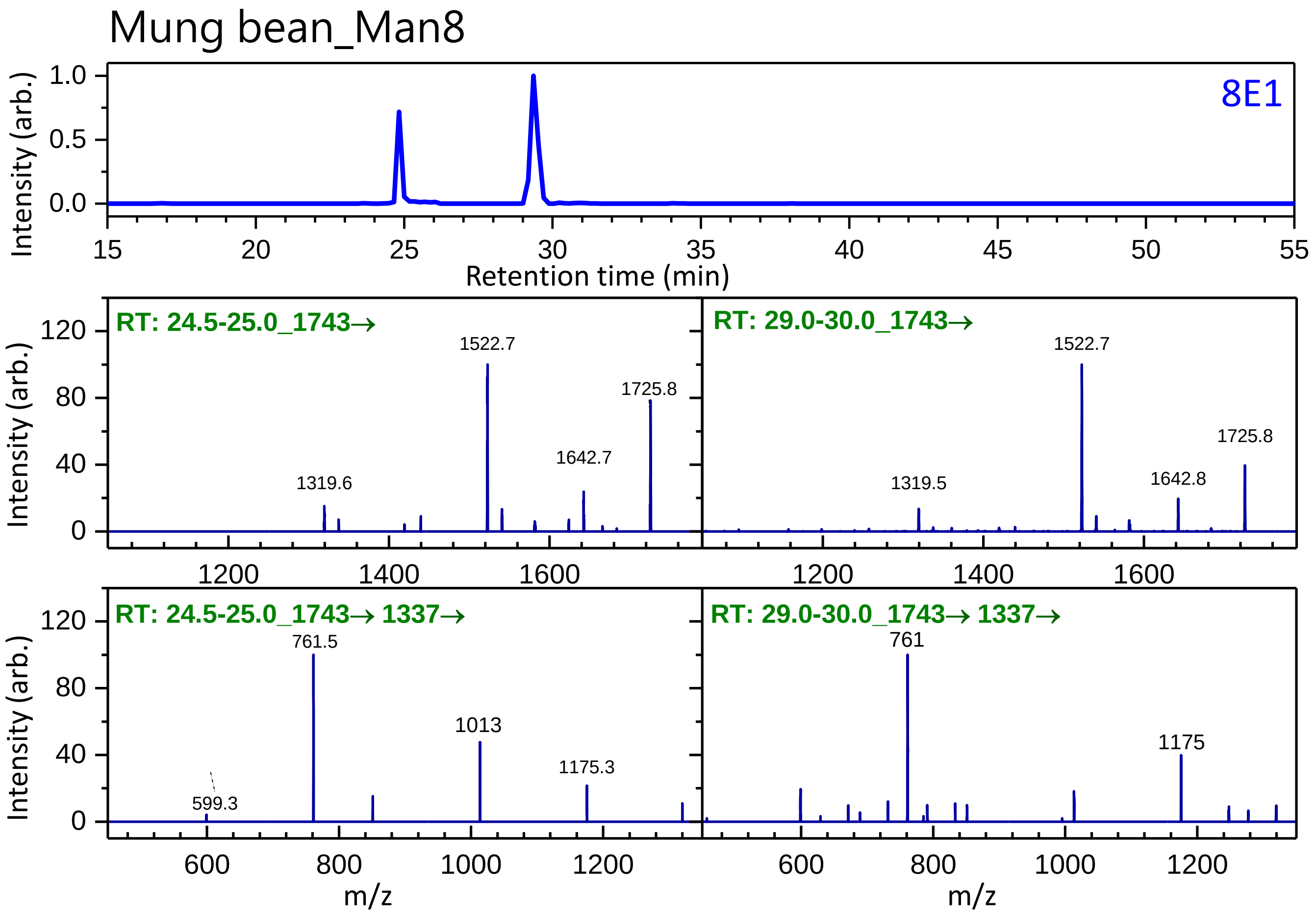

Figure S89. Chromatogram and MS^2 and MS^3 mass spectra of mung bean $Man_8GlcNAC_2$.

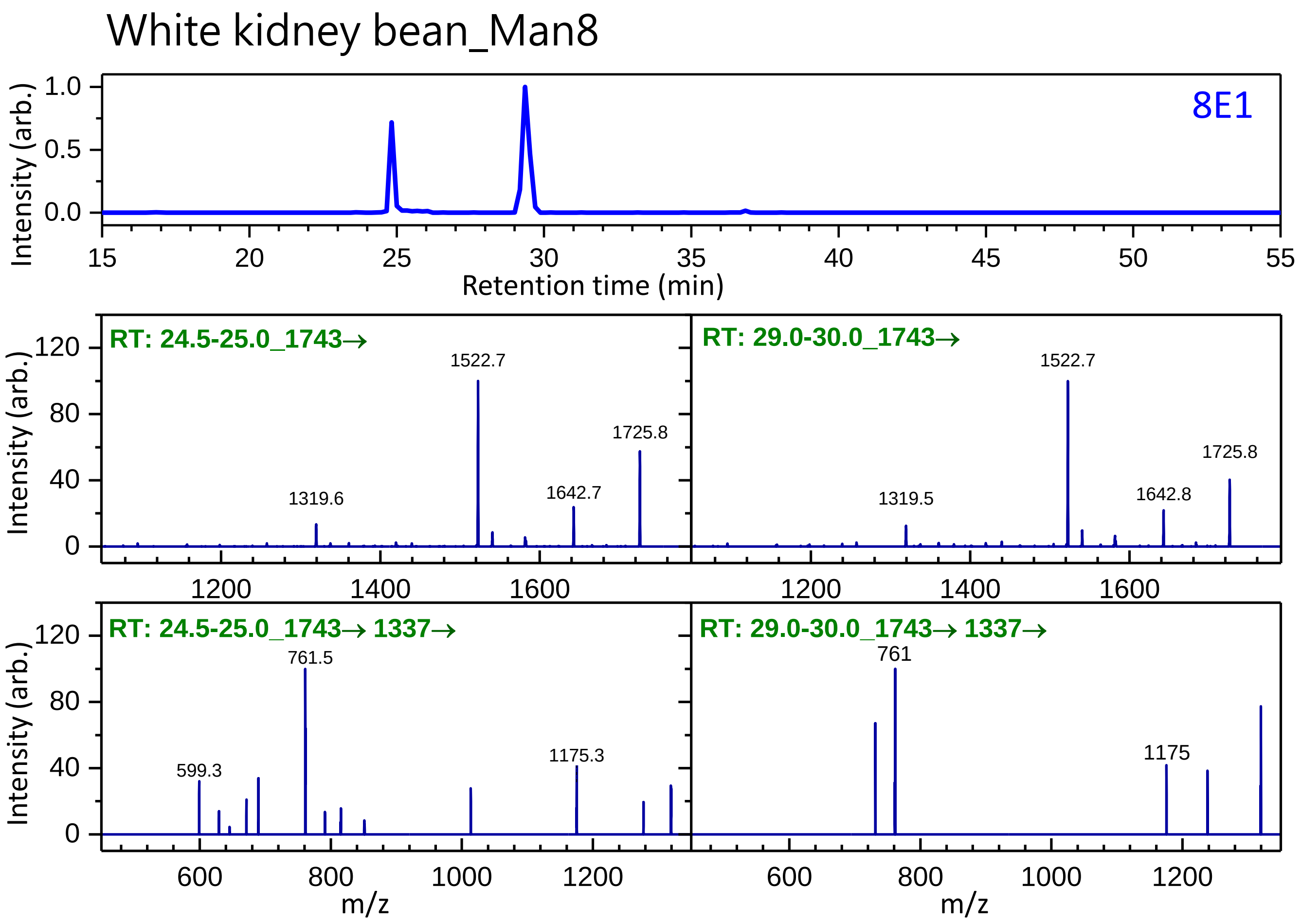

Figure S90. Chromatogram and MS^2 and MS^3 mass spectra of white kidney bean $Man_8GlcNAC_2$.

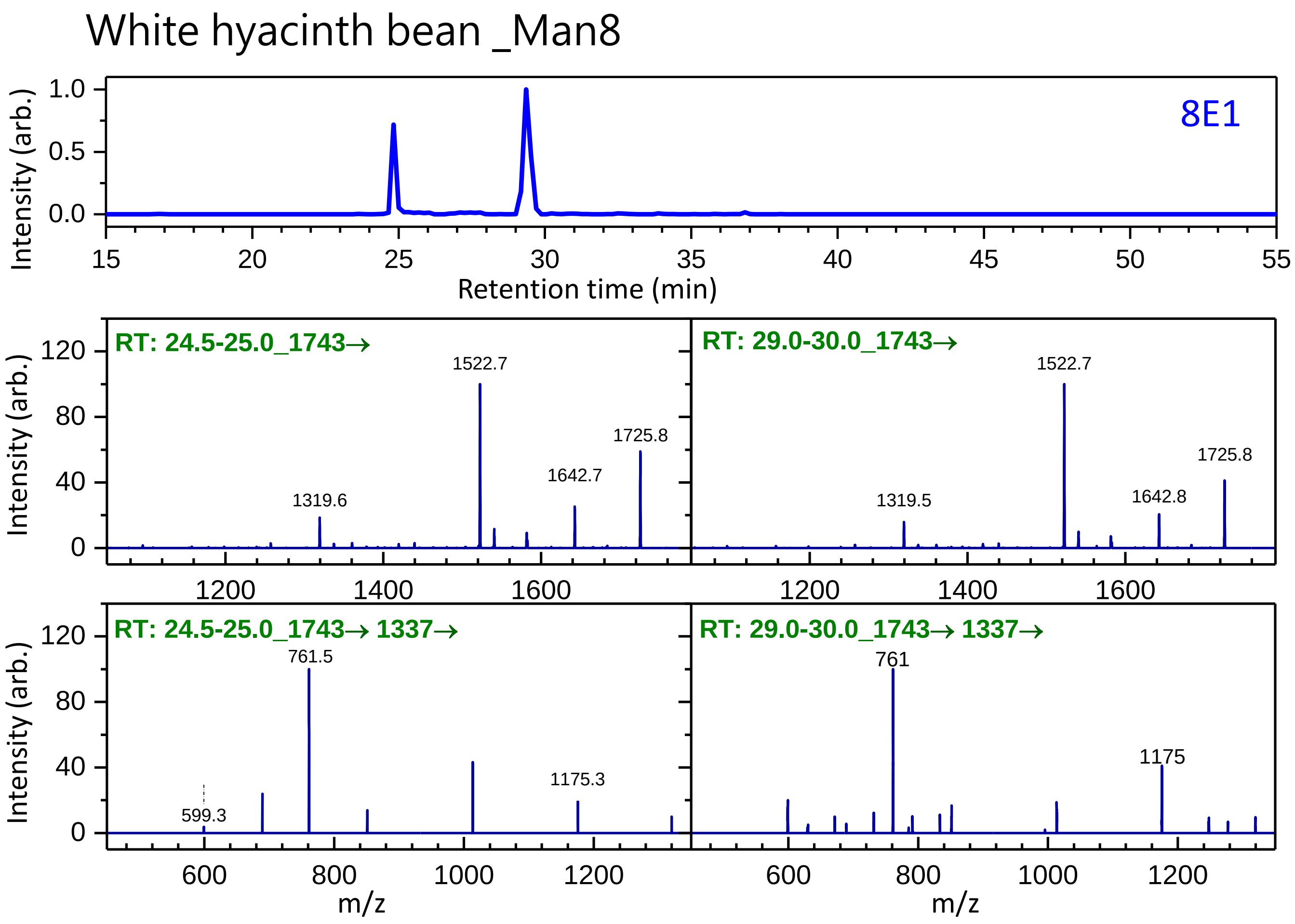

Figure S91. Chromatogram and MS^2 and MS^3 mass spectra of white hyacinth bean $Man_8GlcNAC_2$.

$Man_9GlcNAC_2$

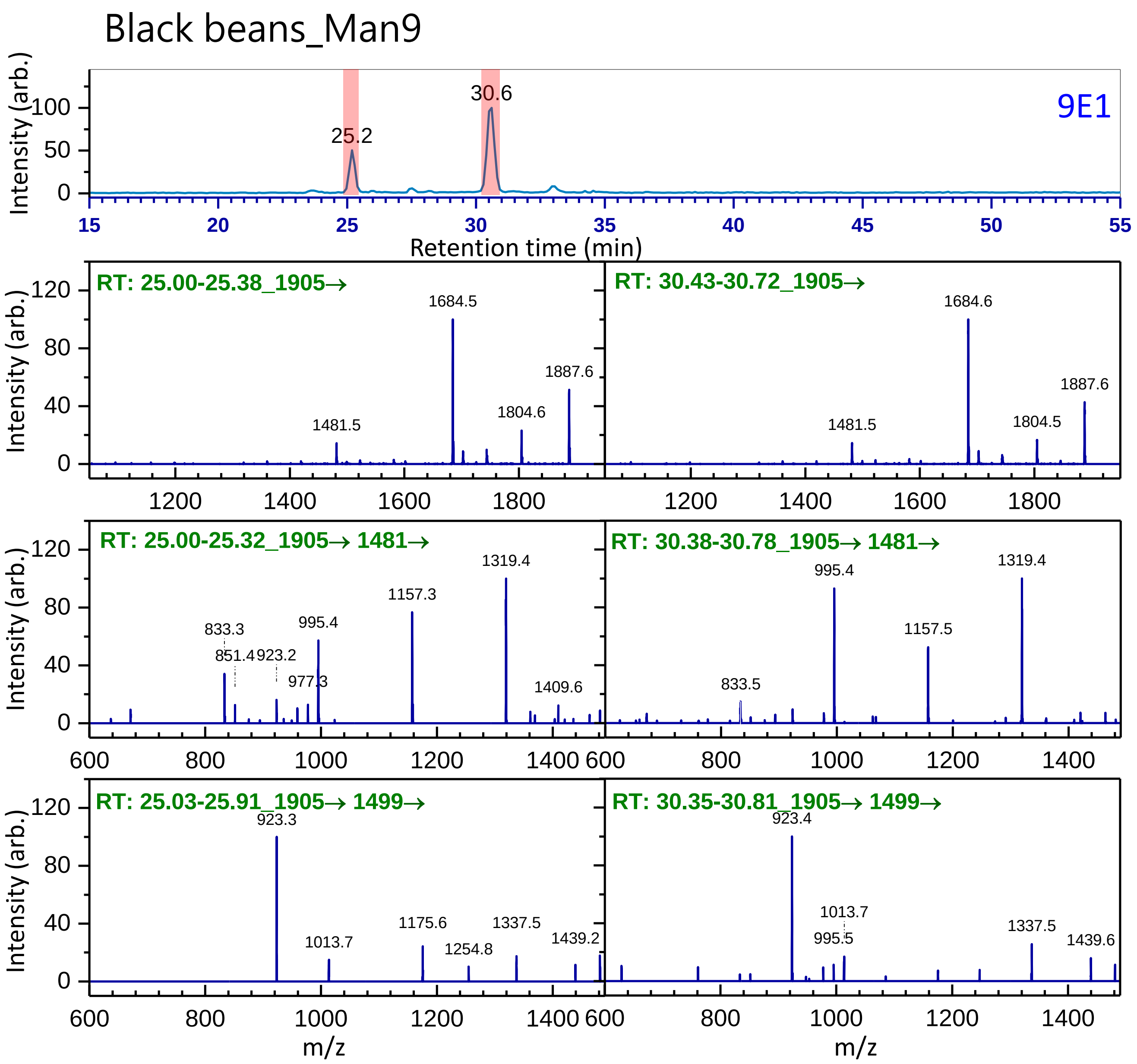

Figure S92. Chromatogram and MS^2 and MS^3 mass spectra of black bean $Man_9GlcNAC_2$.

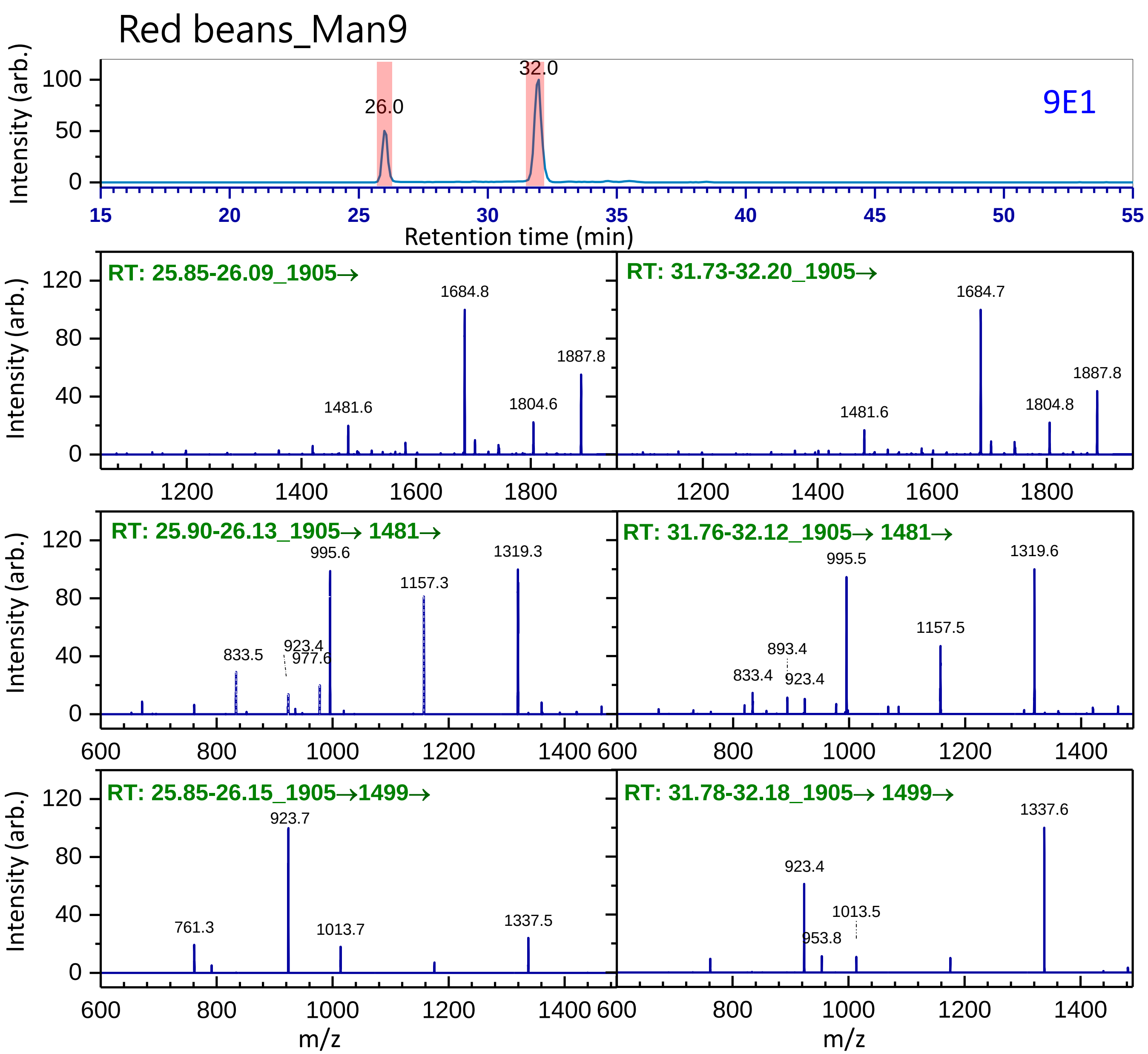

Figure S93. Chromatogram and MS^2 and MS^3 mass spectra of red bean $Man_9GlcNAC_2$.

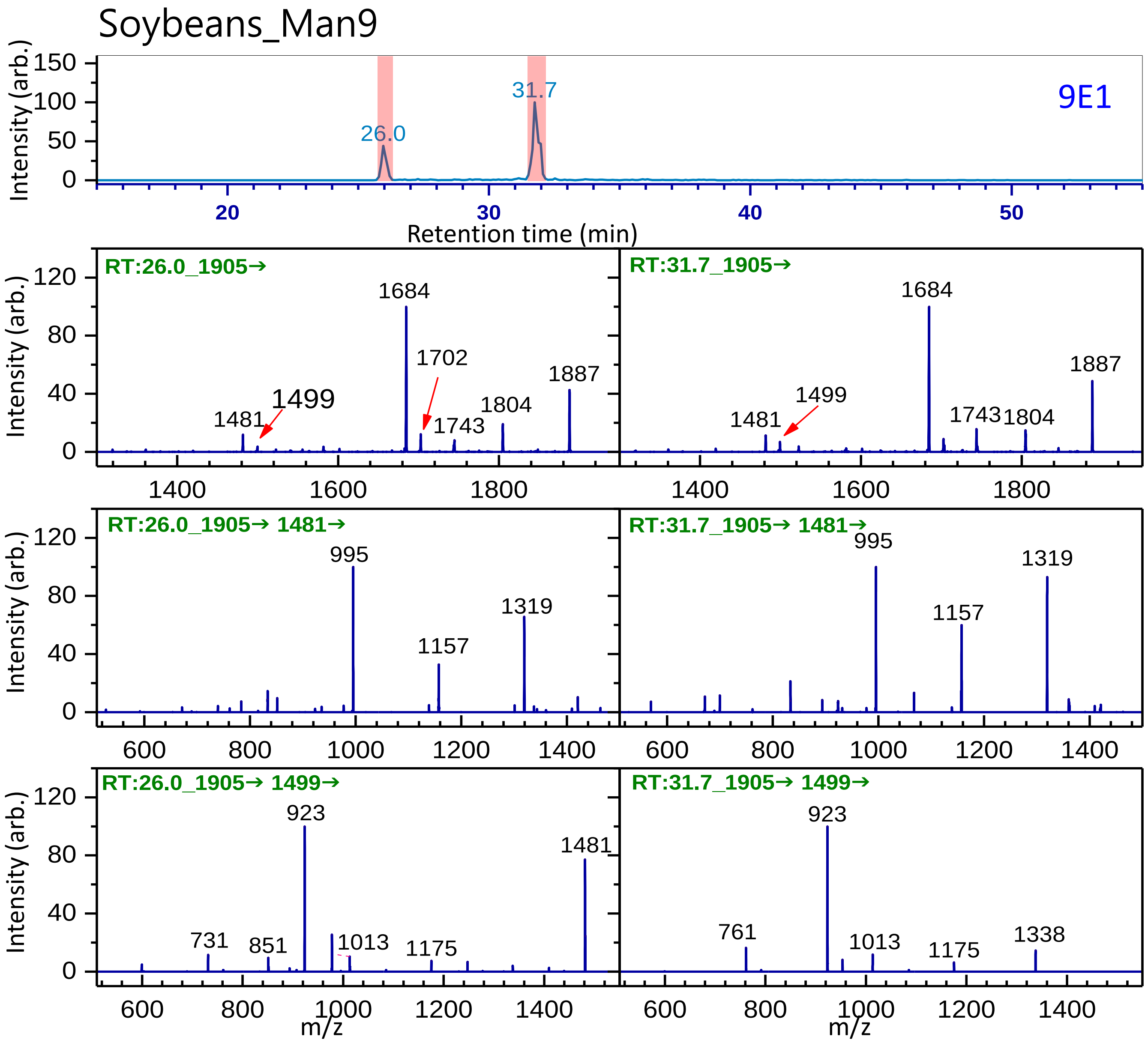

Figure S94. Chromatogram and MS^2 and MS^3 mass spectra of soybean $Man_9GlcNAC_2$.
